# A chemical probe based on the PreQ_1_ metabolite enables transcriptome-wide mapping of binding sites

**DOI:** 10.1038/s41467-021-25973-x

**Published:** 2021-10-06

**Authors:** Sumirtha Balaratnam, Curran Rhodes, Desta Doro Bume, Colleen Connelly, Christopher C. Lai, James A. Kelley, Kamyar Yazdani, Philip J. Homan, Danny Incarnato, Tomoyuki Numata, John S. Schneekloth Jr

**Affiliations:** 1 Chemical Biology Laboratory, National Cancer Institute, Frederick, MD 21702 USA; 2 Center for Cancer Research Collaborative Bioinformatics Resource, National Cancer Institute, National Institutes of Health, Bethesda, MD 20892 USA; 3 Advanced Biomedical Computational Science, Frederick National Laboratory for Cancer Research, Frederick, MD 21702 USA; 4 Department of Molecular Genetics, Groningen Biomolecular Sciences and Biotechnology Institute (GBB), University of Groningen, Groningen, The Netherlands; 5 Department of Bioscience and Biotechnology, Graduate School of Bioresource and Bioenvironmental Sciences, Kyushu University, 744 Motooka, Nishi-ku, Fukuoka-shi Fukuoka, 812-8582 Japan; 6 Biomedical Research Institute, National Institute of Advanced Industrial Science and Technology (AIST), 1-1-1 Higashi, Tsukuba-shi, Ibaraki, 305-8566 Japan

**Keywords:** Chemical tools, Nucleic acids, RNA

## Abstract

The role of metabolite-responsive riboswitches in regulating gene expression in bacteria is well known and makes them useful systems for the study of RNA-small molecule interactions. Here, we study the PreQ_1_ riboswitch system, assessing sixteen diverse PreQ_1_-derived probes for their ability to selectively modify the class-I PreQ_1_ riboswitch aptamer covalently. For the most active probe (**11**), a diazirine-based photocrosslinking analog of PreQ_1_, X-ray crystallography and gel-based competition assays demonstrated the mode of binding of the ligand to the aptamer, and functional assays demonstrated that the probe retains activity against the full riboswitch. Transcriptome-wide mapping using Chem-CLIP revealed a highly selective interaction between the bacterial aptamer and the probe. In addition, a small number of RNA targets in endogenous human transcripts were found to bind specifically to **11**, providing evidence for candidate PreQ_1_ aptamers in human RNA. This work demonstrates a stark influence of linker chemistry and structure on the ability of molecules to crosslink RNA, reveals that the PreQ_1_ aptamer/ligand pair are broadly useful for chemical biology applications, and provides insights into how PreQ_1_, which is similar in structure to guanine, interacts with human RNAs.

## Abstract (web summary)

The small modified nucleotide PreQ1 binds to the PreQ1 riboswitch and regulates gene expression by inducing RNA conformation change. Here the authors design and characterize a specific preQ1-derived probe by x-ray crystallography, mass spectral analysis and transcriptome-wide using Chem-CLIP.

## Introduction

Riboswitches are naturally occurring RNA sequences that influence bacterial gene expression by binding directly to small molecules^1–3^. As they can bind specifically and selectively to small molecules, these important functional elements provide invaluable systems to study RNA-small molecule interactions^4–6^. One such system is the PreQ_1_ riboswitch, which binds to the small, modified nucleotide 7-aminomethyl-7-deazaguanine (known as PreQ_1_). PreQ_1_ riboswitches have been divided into three classes based on differences in function and size. Upon recognition of PreQ_1_, the RNA changes conformation and alters gene expression at either transcriptional level or translational level^7,8^. This system serves as a metabolic feedback sensor for PreQ_1_ levels, allowing bacteria to control the expression of genes involved in one carbon metabolism in response to metabolite levels. While many bacteria contain diverse PreQ_1_-responsive riboswitches in mRNAs, human mRNAs appear to be devoid of analogous elements^9^. To date, the study of PreQ_1_ in human transcriptomes has mostly been limited to covalent modification of tRNAs by queuosine (of which PreQ_1_ is a metabolic precursor in bacteria)^10^. PreQ_1_ has also been utilized in a series of intriguing studies aimed at enzymatic tagging of complex RNAs^11,12^. Owing to the high selectivity and affinity of the PreQ_1_/aptamer interaction, as well as ease of chemical modification, it could find broad use in chemical biology and synthetic biology applications^13^. Additionally, PreQ_1_ is structurally very similar to guanine and thus a PreQ_1_-derived probe could provide insights into how this and other similar nucleobases recognize human RNA structures. Thus, a broad assessment of PreQ_1_ binding in human transcriptomes would not only clarify the selectivity of the PreQ_1_ aptamer interaction but also enable an unbiased examination of metabolite binding to human RNAs.

Reactive molecules that covalently modify RNAs have played a substantial role in understanding RNA biology by probing structure^14,15^, controlling gene expression^16^, imaging RNAs^17,18^, tagging RNAs with functional handles, and demonstrating target engagement for RNA-binding small molecules^19–22^. However, examples of structurally characterized RNA/ligand complexes are relatively rare. Still, highly specific RNA/ligand pairs can find broad use in diverse applications. As interest in RNA as a target for small molecule drugs has increased, so has the need for information about the design of RNA-targeting probes^23–25^. Understanding the chemical features that impact RNA crosslinking efficiency would influence probe design, as well as inform interpretation of results from experiments that use these probes. To date, there has been limited study on the factors that govern covalent crosslinking efficiency to RNA. Furthermore, the impact of tagging small ligands with reactive handles could be substantial. Aptamers can discriminate between highly similar ligands, even distinguishing fluoride from chloride^26^, potentially representing a challenge in probe design. Drawing inspiration from the approaches above, we set out to design covalent probes targeting a PreQ_1_ riboswitch, and to apply them for a broad assessment of binding in complex systems.

The class-I PreQ_1_ riboswitch aptamers (PreQ_1_-I) represent an ideal system for this study due to their relatively small size and its conserved ligand binding sites. For example, the PreQ_1_ riboswitch from *Bacillus subtilis* (*Bs*-preQ_1_-RS) consists of just 34 nucleotides. Upon binding to PreQ_1_, it folds into an H-type pseudoknot; this conformational change results in the transcriptional downregulation of *que*CDEF operon^7,27,28^. Similarly, *Staphylococcus saprophyticus* (*Ss*-preQ_1_-RS) carries a related class-I PreQ_1_ riboswitch that also functions by transcriptional termination^29^. For the PreQ_1_ riboswitch from *Thermoanaerobacter tengcongensis* (*Tt*-preQ_1_-RS) binding to PreQ_1_ also results in the formation of an H-type pseudoknot structure. However, in this case the riboswitch acts at the translational level by regulating the accessibility of the Shine-Dalgarno sequence and ribosome binding^8,30,31^. While the mechanism and sequences of these PreQ_1_-I class riboswitches differ they all have tight and selective interactions with the PreQ_1_ ligand. For example, the *Tt*-preQ_1_-RS has an equilibrium dissociation constant of 2 nM for PreQ_1_^30^ and the *Bs*-preQ_1_-RS constant has been reported as 20 nM from in-ling probing^8^. In addition to this tight binding, these aptamers have been studied extensively using both X-ray crystallography and NMR, providing detailed information about the mode of ligand binding^29–34^. Other studies on this system include how it responds to other natural metabolites, engineering to remodel ligand specificity^8,13,34^, and investigations for inhibitor design^29^. PreQ_1_ riboswitches from other organisms have been studied in detail as well, revealing multiple distinct classes of independently evolved riboswitches that function by different mechanisms^7^. In fact, PreQ_1_ riboswitches are among the most frequently occurring riboswitches in Nature^35^. This example of convergent evolution demonstrates a remarkably high diversity of RNA sequences that recognize PreQ_1_ with high affinity and selectivity. Given the extensive work reported on the PreQ_1_ riboswitches, its potential for use in chemical biology and synthetic biology applications, the role of PreQ_1_ in influencing gene expression, the frequency of natural aptamers for PreQ_1_, and the similarity of PreQ_1_ to guanine, efforts to probe the binding of this metabolite to human RNAs are warranted.

Here, we report the design and evaluation of a series of covalent probes for the PreQ_1_-I class riboswitch. In total, a series of 16 probes are reported, including both electrophilic and photochemically activated crosslinkers. Since the PreQ_1_-I class riboswitches have a small aptamer domain and have conservation of PreQ_1_ binding, we utilize multiple PreQ_1_-I class aptamers (Bs*, Ss, Tt*) from different species in this study to ensure that the best probes could be broadly recognized by these riboswitches. Gel-based and mass spectrometry experiments are conducted to quantify crosslinking efficiency and characterize the covalent adduct. We report a remarkable effect of linker length and crosslinker structure on crosslinking efficiency. None of the electrophilic probes showed evidence of covalent modification, however photocrosslinking probes had varying efficiency. One of these probes was selected for further in-depth characterization. The probe was found to photocrosslink only to the bases of the aptamer, not sugar or phosphate moieties. Additionally, the photocrosslinking probe retained activity in a transcriptional termination assay. An X-ray co-crystal structure of the probe in complex with the aptamer demonstrated that the mode of binding was unperturbed by the introduction of the crosslinker sidechain. We also demonstrate the potential utility of this approach for labeling, target engagement and enrichment studies. Transcriptome-wide mapping studies (Chem-CLIP) in the presence of the bacterial aptamer reveal an unexpectedly high specificity for the bacterial aptamer over all other human RNAs. In total human RNA (without aptamer added), the probe enriched several transcripts significantly. Validation of these genes with competitive assays revealed several specific interactions between PreQ_1_ and human RNAs.

## Results

### Synthesis and evaluation of electrophilic probes

To design covalent probes targeting the PreQ_1_-I class riboswitch aptamers, a structure-informed strategy was taken. From multiple available structures of ligand-bound riboswitches it was apparent that the pendant amine from PreQ_1_ was involved with binding but was also mostly solvent exposed^29,30,32–34^. Furthermore, proximal to the binding site were multiple different potential nucleophilic groups. For example, the 2′-OH of G11 or base side chains of G4 and G5 from the *Thermoanaerobacter tengcongensis* (*Tt*-preQ_1_-RS)(PDB: 3Q50) aptamer are all near the binding site^29–32^. We thus designed and synthesized a series of probes that contain commonly used electrophilic warheads for covalent modifications that could potentially react with these groups. As described in Table 1, these compounds included Michael acceptors (**1**–**3**), acylating reagents (**4**–**9**), and the chlorambucil derivative that has been used in Chem-CLIP experiments (**10**) and has been reported to successfully alkylate RNA in a non-specific manner^36,37^.

**Table 1:** Structures and crosslinking efficiencies for electrophilic and photoaffinity probes used for proximity induced covalent modification of the *Tt*-preQ_1_-RS aptamer (compounds **1–10**) or *Bs*-preQ_1_-RS aptamer (compounds **11–16**). Error bars indicate standard deviation, *n* = 3. (Molar ratio between the aptamer and probe is 1:50).

Compound ID	Compound structure	% Crosslink efficiency (PAGE)
1		Not observed
2		Not observed
3		Not observed
4		Not observed
5		Not observed
6		Not observed
7		Not observed
8		Not observed
9		Not observed
10		Not observed
11		31.3 ± 2.9
12		49.2 ± 3.2
13		39.9 ± 5.3
14		Trace
15		22.0 ± 2.2
16		19.4 ± 4.4
17		Not determined

The reactivity of each of these probes towards PreQ_1_-I class riboswitch aptamers was evaluated using mass spectrometry (e.g., MALDI-TOF) and denaturing PAGE experiments. Initial in vitro analysis of the reaction mixtures between the *Tt*-preQ_1_-RS aptamers and the electrophilic probes showed some evidence of higher molecular weight species corresponding to potential adducts in low yields. However, these species did not correlate to a specific product. Furthermore, when these reaction mixtures were subjected to denaturing PAGE analysis, no evidence was seen for specifically modified RNA (Supplementary information Fig. S1). Efforts to optimize the reaction conditions by altering reaction temperature or buffer conditions did not affect the outcome. Based on these findings, we speculate that these electrophiles may simply not be reactive enough to yield high percentages of covalently modified RNAs, or that the electrophiles could not achieve the proper geometry for covalent modification. Similarly, others have reported that modification of the amine at the C7 position of PreQ_1_ with various aminoalkyl chains results in decreased activity in an in vivo *E. coli* reporter system^38^. To overcome this challenge, we turned our attention towards the synthesis and evaluation of more reactive, diazirine-containing photoaffinity crosslinkers.

### Design and optimization of photocrosslinking covalent probes

**Fig. 1:**
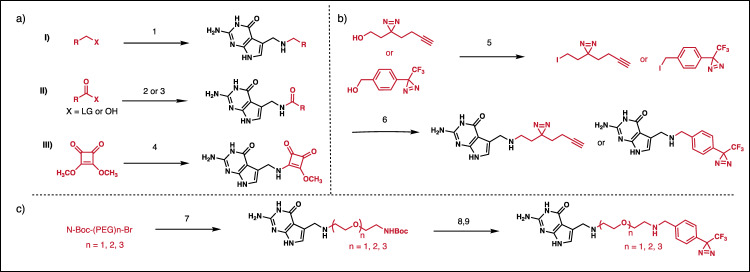
Synthetic route for the preparation of chemical probes. Synthesis of electrophilic (**a**) and photoaffinity probes (**b**, **c**). (1) PreQ_1_, K_2_CO_3_, DMF, rt, 1 h; (2) PreQ_1_,TEA, DMF, rt; (3) PreQ_1_, HATU, DIEA, DMF, 0 °C to rt 1 h; (4) PreQ_1_, TEA, DMF, rt, 18 h; (5) Ph_3_P, Imidazole, I_2_, DCM, 0 °C, 4 h; (6) PreQ_1_, K_2_CO_3_, DM rt, 2 h (in dark); (7) PreQ_1_, K_2_CO_3_, DMF, rt, 1 h; (9) Trifluoroacetic acid/DCM, 0 °C rt, 30 min; (8) PreQ_1_, K_2_CO_3_, DMF, rt, 2 h (in dark).

Similar to electrophilic probes, photoaffinity probes are commonly employed for labeling studies with proteins^39,40^. Diazirine functionalized nucleosides have been used previously to map nucleic acid-ligand interactions or conjugate dyes to aptamers^19–22^. However, typically only one probe was reported, and the effect of linker structure or crosslinking group was not broadly evaluated. In order to assess how photoaffinity linker structure influences crosslinking efficiency, a series of photoaffinity probes were designed and synthesized (Fig. 1 and Table 1). Additional details for the synthesis and characterization of these compounds are provided in the supplementary information (Section 3: synthesis and characterization). Included in this series are probes varied in crosslinker structure (stabilized and unstabilized diazirines), as well as linker length^41,42^. The *Bs*-preQ_1_-RS was treated with 50-fold excess of probe in the presence of riboswitch buffer containing 2 mM MgCl_2_, irradiated with 365 nm light sources for 15 minutes, and PAGE and MALDI MS analysis was used to assess the formation of crosslinks. Gratifyingly, the expected adducts were observed in all cases, however only trace crosslinking was observed with **14** in MALDI. Crosslinking efficiencies were above 30% for unstabilized probes **11**, **12**, and **13**, and approximately 20% with stabilized probes **15** and **16** (Supplementary information Fig. S2).

### Effect of linker length and composition on photocrosslinking efficiency

The behavior of compounds **14**–**16** highlight the importance of the linker structure and length in preserving the native interaction between PreQ_1_ and the riboswitch as well as enabling crosslinking. Crosslinking was not observed for **14** in denaturing PAGE analysis, potentially due to steric clashes that could occur between the phenyl ring in the linker and the RNA that prevent the compound from inserting fully into the binding pocket. The results for compounds **15** and **16** support this conclusion as some crosslinking can be restored when the phenyl ring is spaced further away from the PreQ_1_ core by PEG chains. Overall, we showed that both compounds **11** and **13** can modify the aptamer with high efficiency (>30%) under these conditions. We chose to proceed with compound **11** because it retained the positive charge in the natural PreQ_1_ ligand and contained an alkyne handle for further modification.

**Fig. 2:**
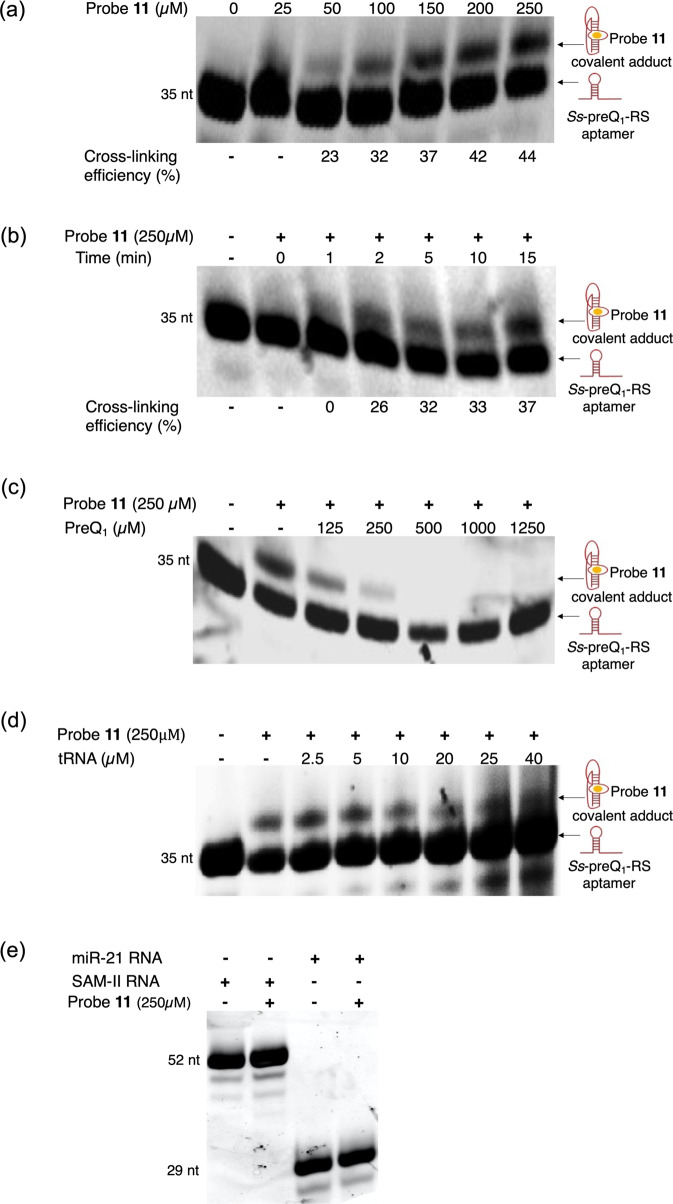
Biochemical optimization of photocrosslinking and competition experiments to evaluate the specificity of compound **11** for the *Ss*-preQ_1_-RS aptamer. **a** Dose-dependent crosslinking efficiency of compound **11**. **b** Time-dependent crosslinking efficiency of compound **11**. Competition experiments in the presence of **c** PreQ_1_ and **d** tRNA. **e** PAGE analysis to assess crossreactivity of compound **11** with other structured RNAs shows no quantifiable modification of either miR-21 RNA or SAM-II RNA. Values for crosslinking efficiency are reported as the mean ± standard deviation of three replicate experiments. Source data are provided as a Source Data file.

We next evaluated the effects of crosslinking **11** to the PreQ_1_ aptamer as a function of both time and concentration. In both denaturing PAGE and MALDI-TOF assays, clear dose and time-dependent crosslinking was observed (Figs. 2a, b and S3). Importantly, even at higher doses or longer times of irradiation, a single crosslinked species was the predominant product, indicating a highly specific crosslinking event.

### Competitive crosslinking experiments demonstrate selectivity in vitro

With an optimized probe in hand, we next explored whether the photoaffinity labeling with compound **11** achieves proximity induced site-specificity or if the observed adducts were due to indiscriminate reactivity of diazirine with the RNA. Consequently, we performed labeling experiments in the presence of increasing concentrations of unmodified PreQ_1_. PreQ_1_ showed a dose-dependent inhibition of crosslinking of **11**, with complete inhibition of crosslinking observed at higher concentrations (Fig. 2c). To further establish the selectivity of **11**, we evaluated the labeling efficiency in the presence of up to tenfold excess of tRNA with respect to the aptamer. No significant decrease in the labeling efficiency was observed under these conditions, indicating that **11** retains selectivity for the aptamer in the presence of excess tRNA (Fig. 2d). We also performed labeling experiments with other structured RNAs such as microRNA 21 (miR-21) and SAM II riboswitch with compound **11**. No detectable high molecular weight (slow migrating) crosslink product was observed in denaturing PAGE gels under these conditions (Fig. 2e).

### Mass spectrometry and crystallography establish mode of interaction with 11

**Fig. 3:**
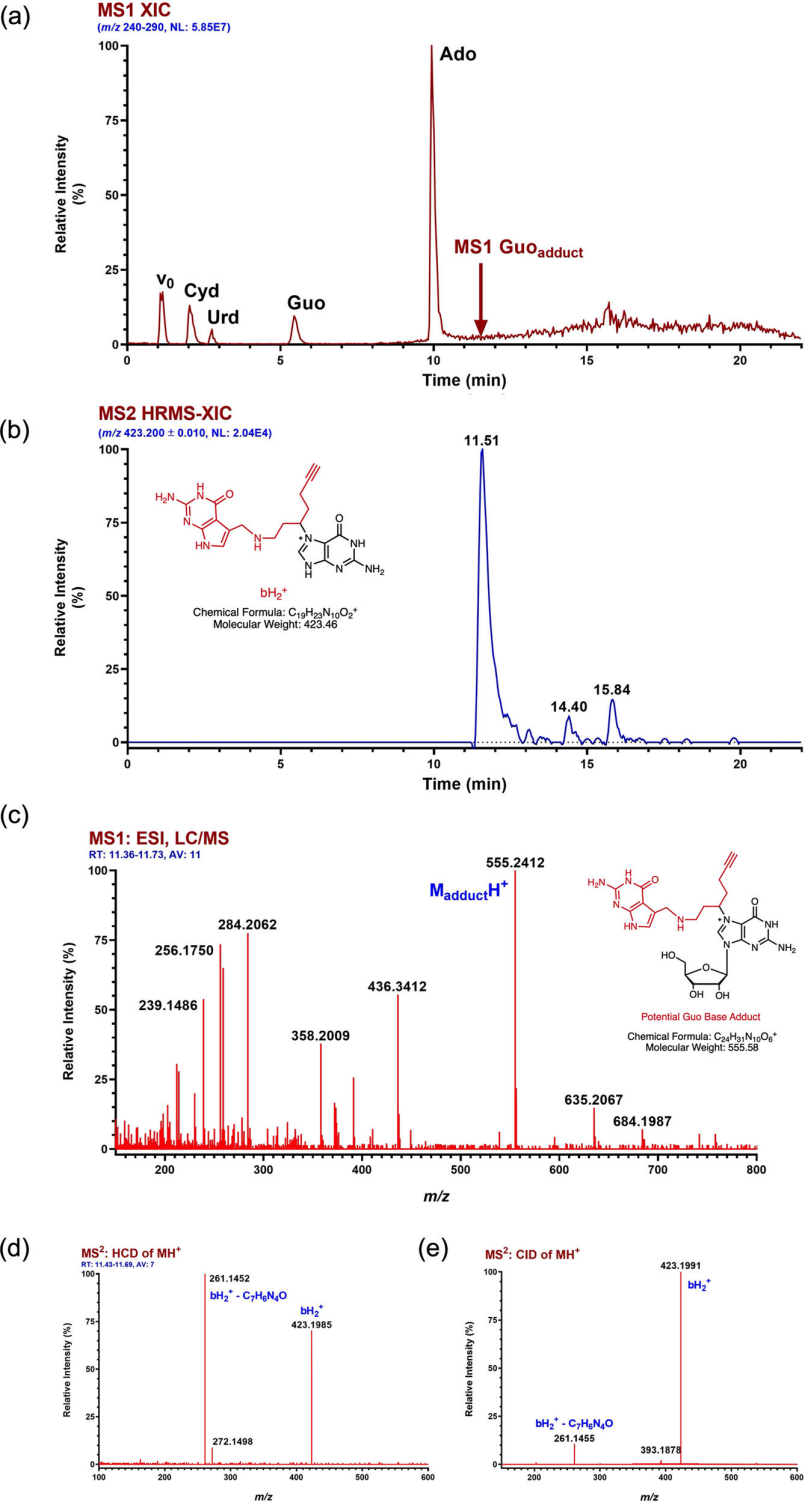
Probe **11** selectively crosslinks to guanosine bases in the PreQ_1_ aptamer. High-resolution accurate mass (HRAM) and positive ion LC/MS/MS analysis of the nucleoside digest resulting from adducted *Ss*-preQ_1_-RS (RNA 35-mer). **a** MS1 extracted ion chromatogram (XIC) for *m*/*z* 240–290 showing the response for unmodified RNA nucleosides. HPLC void volume is indicated by v0 and the elution time of the major RNA probe adduct is indicated by a red arrow. **b** MS2 (CID of Guo-adduct MH+) HRAM XIC for bH2+ , the ion indicating that adduct formation occurs on the base. Extracted ion mass tolerance is ±10 mDa. The ion structure represents one of several possibilities for adduct and charge location. **c** Averaged and background-subtracted high-resolution MS1 spectrum for the major Guo-adduct located through HRAM XIC analysis of the theoretical MH+ (*m*/*z* 555.2423) for potential adducts. A plausible structure of the adduct is illustrated. **d** Full scan high-resolution MS/MS spectrum for HCD of MH+ of the Guo-adduct eluting at 11.51 min in **a** & **b**. **e** Full-range high-resolution MS/MS spectrum for CID of the same Guo-adduct eluting at 11.51 min in **a** & **b**.

In an effort to probe the site-specificity of the crosslink further, the photocrosslinked RNA was subjected to a nucleoside digestion using NEB Nucleoside Digestion Mix®. The resulting reaction mixture was analyzed by Orbitrap-Liquid chromatography–mass spectrometry (LC/MS) and we identified guanosine-compound **11** adduct as a primary modified species (Fig. 3a, b). Moreover, further fragmentation and MS/MS analysis of guanosine adduct using a higher-energy collisional dissociation (HCD) and collision-induced dissociation (CID) techniques showed either a loss of the sugar or the PreQ_1_ portion of the probe (Fig. 3c–e). Based on these fragmentation patterns, we conclude that the photoaffinity probe exclusively reacts with guanosine on the nucleobase rather than on the sugar. These findings are consistent with proximity-induced alkylation of guanines located near the binding site of the ligand as observed in crystal structures.

**Fig. 4:**
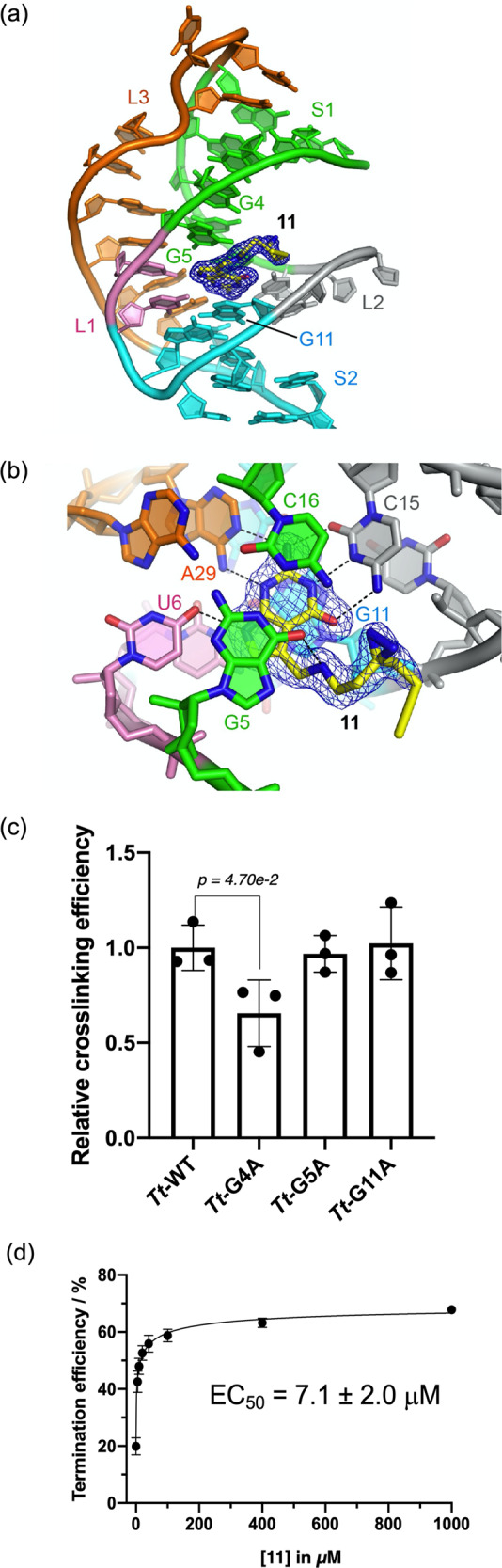
An X-ray co-crystal structure and functional assessment of **11**. **a** Co-crystal structure showing interaction of compound **11** with the *Tt-*preQ_1_-RS aptamer (ab_13-14) solved at 1.57 Å resolution. S1, S2, L1, L2, and L3 are colored green, cyan, pink, gray, and orange, respectively. The m*F*_o_ – D*F*_c_ electron density maps for the compound are colored blue and contoured at 3.0 σ. **b** Detail of ligand:RNA interaction showing conservation of contacts seen with unmodified PreQ_1_. The nucleotides that interact with the compound are labeled, and hydrogen bonds are indicated as dotted lines. Residues at the site of the interaction are labeled to show the proximity of G5 and G11 to the diazirine. The m*F*_o_ – D*F*_c_ electron density maps for the compound are also shown as in **a**. **c** Bar graph representing the photocrosslinking efficiency of wild-type *Tt*-preQ_1_-RS aptamer (*Tt*-WT) and three different mutant aptamers (*Tt*-G4A, *Tt*-G5A, and *Tt*-G11A) to Probe **11**. **d** Single-round transcription termination assay results for compound **11**. Quantification of transcription termination efficiency with increasing concentrations of **11** was calculated based on the band intensity of read-through transcription product (RT) and terminated transcription product (T). Data are presented as the mean ± SEM (*n* = 3) of three independent experiments. Statistical significance was calculated by two-tailed *t*-test analysis. Source data are provided as a Source Data file.

To further validate the binding of **11** to the PreQ_1_ aptamer, we performed X-ray crystallography with the PreQ_1_ aptamer from *T. tengcongensis* (*Tt*-preQ_1_-RS), using a previously reported method^29^. Efforts to determine the X-ray crystal structure of **11** photocrosslinked to the *Tt*-PreQ_1_-RS aptamer were unsuccessful. Co-crystals were obtained using wild type (WT) and an abasic mutant at positions 13 and 14 (ab_13-14) without photocrosslinking, and the complex structures were solved by the molecular replacement method at 2.80 and 1.57 Å resolution respectively (Fig. 4a, b and Fig. S4, and Table S1). Positions A13 and A14 of the *Tt*-preQ_1_-RS are important for stabilization of the expression platform^31^. However, when we co-crystallized the wild-type *Tt*-preQ_1_-RS with the compound **11**, only small co-crystals were obtained that diffracted to 2.8 Å resolution. In contrast, when using abasic mutant at positions 13 and 14, larger co-crystals were obtained that diffracted to 1.6 Å resolution. Importantly, the binding mode of the compound is conserved between the WT and abasic mutant crystals and the RNA structures are quite similar with the exception of the nucleotides from positions 12 to 14. No specific interaction is observed between the alkyne handle and the RNA, suggesting that positions 12 to 14 are not involved in the direct interaction for binding of the compound **11**. Based on these findings, it was concluded that a high-resolution co-crystal structure of the abasic mutant is more informative in terms of evaluating the chemical determinants of this interaction.

From inspection of the structure, it is clear that the PreQ_1_ portion of **11** binds to the *Tt*-preQ_1_-RS aptamer in an analogous mode to the native ligand, making near identical contacts^29–31^. As shown in Fig. 4b, the methylamine group hydrogen-bonds with O6 of G5. This hydrogen bonding pattern is similar to the interaction observed in our previously reported structure (PDB ID: 6E1W^29^), as well as that of the Bs riboswitch structure (PDB ID: 3K1V^28^). However, the hydrogen bond between N7 of G5 and the methylamine group was not observed in the present crystal structure. This is likely due to the impact of the extended linker on N7, which is flexible and may interfere with this interaction. The sidechain of the probe projects out of the binding pocket toward the solvent and is proximal to two guanosines (G4 and G5) that it could potentially modify upon irradiation. Thus, it is clear that modification of the exocyclic amine of PreQ_1_ does not dramatically alter its mode of binding to RNA, though it does decrease affinity. To further identify which guanosine residue in the active site is primarily modified by **11**, we designed three different mutants for *Tt*-preQ_1_-RS aptamers (G4A, G5A, and G11A) by replacing each guanine near the binding site with an adenine. When we performed photocrosslinking experiments for the wild type *Tt*-preQ_1_-RS aptamer and the three mutants, we observed approximately 40% reduction in crosslinking efficiency to the G4A mutant (*Tt*-G4A). In contrast, the other two mutants (*Tt*-G5A and *Tt*-G11A) showed similar crosslinking efficiency to the wild type *Tt*-preQ_1_-RS aptamer (*Tt*-WT) (Fig. 4c and Fig. S5). Thus, the identity of the nucleotide at position 4 is most important for photocrosslinking.

### Probe 11 retains transcriptional termination activity

Single-round transcription termination assays were performed to assess the function of **11**. We used a previously reported protocol to assess the activity of both the cognate ligand and the photocrosslinking probe^29,43^. The unmodified ligand (PreQ_1_) achieved a maximal termination efficiency of ~85% with a EC_50_ of 7.4 nM, while probe **11** achieved a maximal termination efficiency of ~65% with a EC_50_ of 7.1 µM (Fig. 4d and Fig. S6), demonstrating that probe **11** functions in a similar way to the cognate ligand, albeit requiring a higher concentration. The decreased activity in the termination assay is consistent with decreased affinity of **11** when compared to the cognate ligand (Fig. S7). In Microscale thermophoresis (MST) experiments with both the *Bs*-preQ_1_-RS and *Tt*-preQ_1_-RS aptamers, we observed that probe **11** binds approximately 50-fold weaker than the unmodified ligand. (Fig. S7). Importantly, modification of the N7 of PreQ_1_ with alkyl tethers has been shown to reduce cell activity by others as well^38^. Since the termination assays were performed without irradiation, these experiments confirm that the presence of the photocrosslinker sidechain does not ablate the inhibitory activity of the ligand or negatively impact selectivity.

### Probe 11 retains selectivity in cell lysates and total human RNA

Having demonstrated the selectivity of **11** towards the PreQ_1_ aptamer in vitro and confirmed that the photocrosslinking probe binds to the RNA like the unmodified ligand, we next explored the potential applications of ligand-based strategy for labeling applications, including fluorophore- and biotin-labeling^17,19–22^. To do this, we synthesized biotin-conjugated **11** (Bio-**11**) using copper catalyzed click chemistry with biotin-PEG_3_-azide and performed photocrosslinking experiments with the *Ss*-preQ_1_-RS under our optimized reaction conditions. MALDI-TOF analysis of the reaction mixture showed the expected molecular weight adducts (Supplementary information Fig. S8). Concurrently, the photocrosslinked *Ss*-preQ_1_-RS aptamer was also subjected to copper catalyzed click reaction with biotin-PEG_3_-azide and Cy5-picolyl azide and the expected click product was observed in both experiments by mass spectrometry and denaturing PAGE analysis (Supplementary information Figs. S9 A and B and S10).

**Fig. 5:**
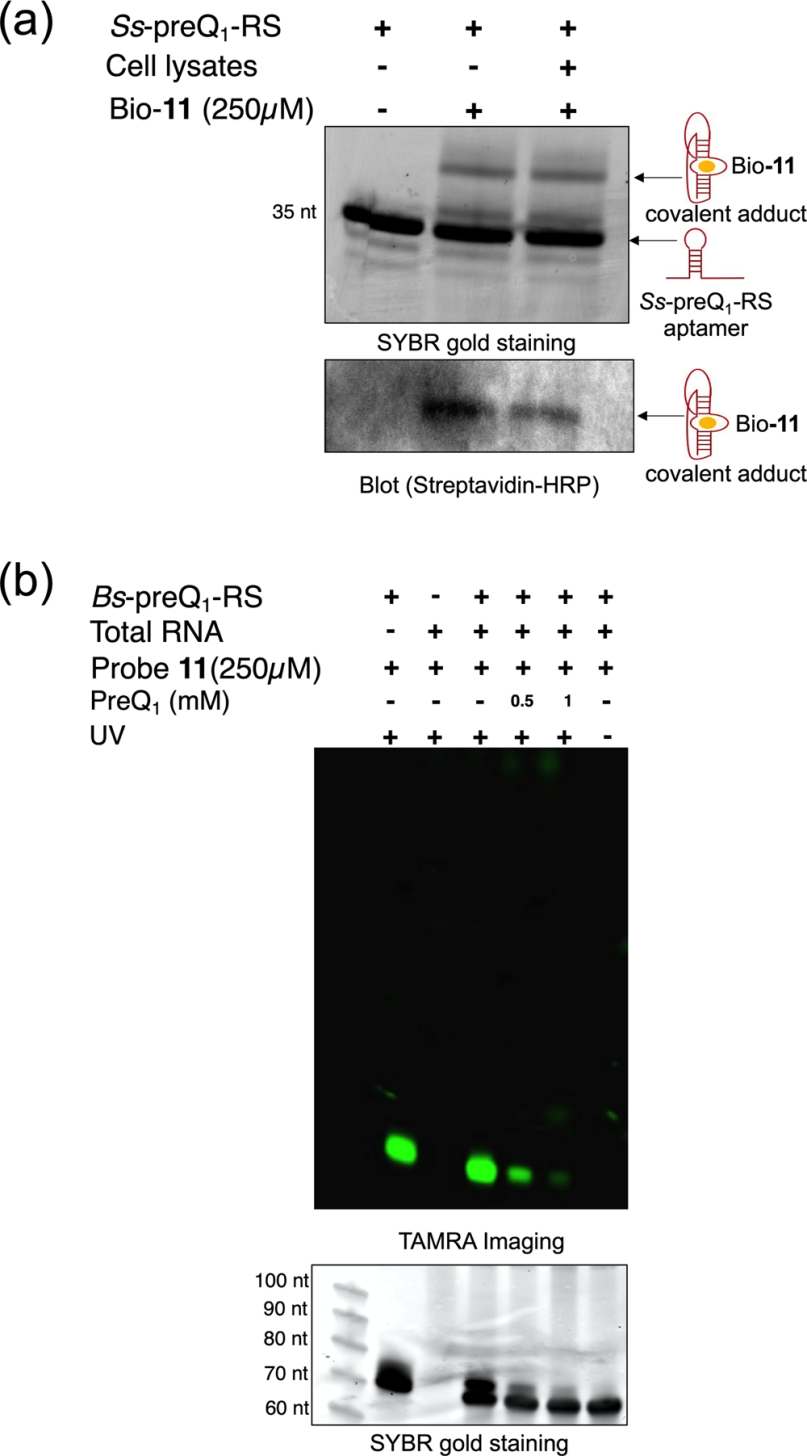
Diazirine probe **11** selectively labels the PreQ_1_ aptamer in cell lysates. **a** In denaturing PAGE experiments Bio-**11** selectively crosslinks the *Ss*-preQ_1_-RS aptamer in the presence of cell lysates. The crosslinked product is observed by SYBR gold staining and streptavidin-HRP detection. **b** Competitive photocrosslinking experiments with *Bs*-preQ_1_-RS full-length aptamer (70 nts) spiked into MCF-7 total RNA and imaged by TAMRA labeling (top) and SYBR gold stain (bottom). Source data are provided as a Source Data file.

Bio-**11** was prepared and added to MCF-7 cell lysates that had been supplemented with *Ss*-preQ_1_-RS aptamer. After irradiation, the lysates were analyzed by denaturing PAGE. As shown in Fig. 5a, when the gel was stained with SYBR gold stain, a crosslinked higher molecular weight band was observed. In order to confirm the presence of the biotinylated, crosslinked product, RNA samples from the gel were then transferred to a positively charged nylon transfer membrane and the blot was incubated with streptavidin-HRP overnight. As shown in Fig. 5a, biotin modified RNA bands were only observed when the lysates had been photoirradiated in the presence of probe Bio-**11**.

Next, we demonstrated that **11** could be used for both the photocrosslinking and biotin labeling in a complex environment. We isolated total cellular RNA from MCF-7 cells, added 1 µM of *Bs*-preQ_1_-RS (a 70 nt construct containing both the aptamer and expression platforms), and treated with compound **11** in riboswitch buffer. After incubation, samples were irradiated at 365 nm for 15 minutes. Subsequently, we performed copper catalyzed click reaction with TAMRA azide and ran the samples on denaturing PAGE. In the absence of the MCF-7 total RNA, a strong TAMRA fluorescent band was observed at the expected molecular weight indicating that the photocrosslinking and click reactions were successful (Fig. 5b, lane 2). Additionally, only weak fluorescent signals were observed when the *Bs*-preQ_1_-RS aptamer was not added into the mixture of **11** and MCF-7 total RNA (Fig. 5b, lane 3). In contrast, as shown in lane 4 of Fig. 5b, compound **11** was able to selectively modify the PreQ_1_ riboswitch even in the presence of other cellular RNAs.

Consistent with earlier observations with the *Ss*-preQ_1_-RS aptamer, photocrosslinking efficiency is significantly reduced in the presence of free PreQ_1_ competitor (Fig. 5b, lanes 5 & 6). This observed competition between **11** and PreQ_1_ demonstrates the importance of proximity induced site-specific modification within the binding pocket. Additionally, no fluorescent band was observed when the reaction mixture was not irradiated with UV, confirming that irradiation of **11** is required for detection of the aptamer with this method (Fig. 5b, lane 7). In comparison, when compound **17**, a small molecule containing alkyne-diazirine fragment that lacks the PreQ_1_ targeting portion, was used to treat total RNA under similar conditions, no detectable labeling of the riboswitch was observed, again confirming that the recognition event between the PreQ_1_ scaffold of **11** and the aptamer is critical for diazirines to be useful as photocrosslinking partners (Supplementary information Fig. S11).

### Chem-CLIP sequencing experiments confirm target engagement in total human RNA

**Fig. 6:**
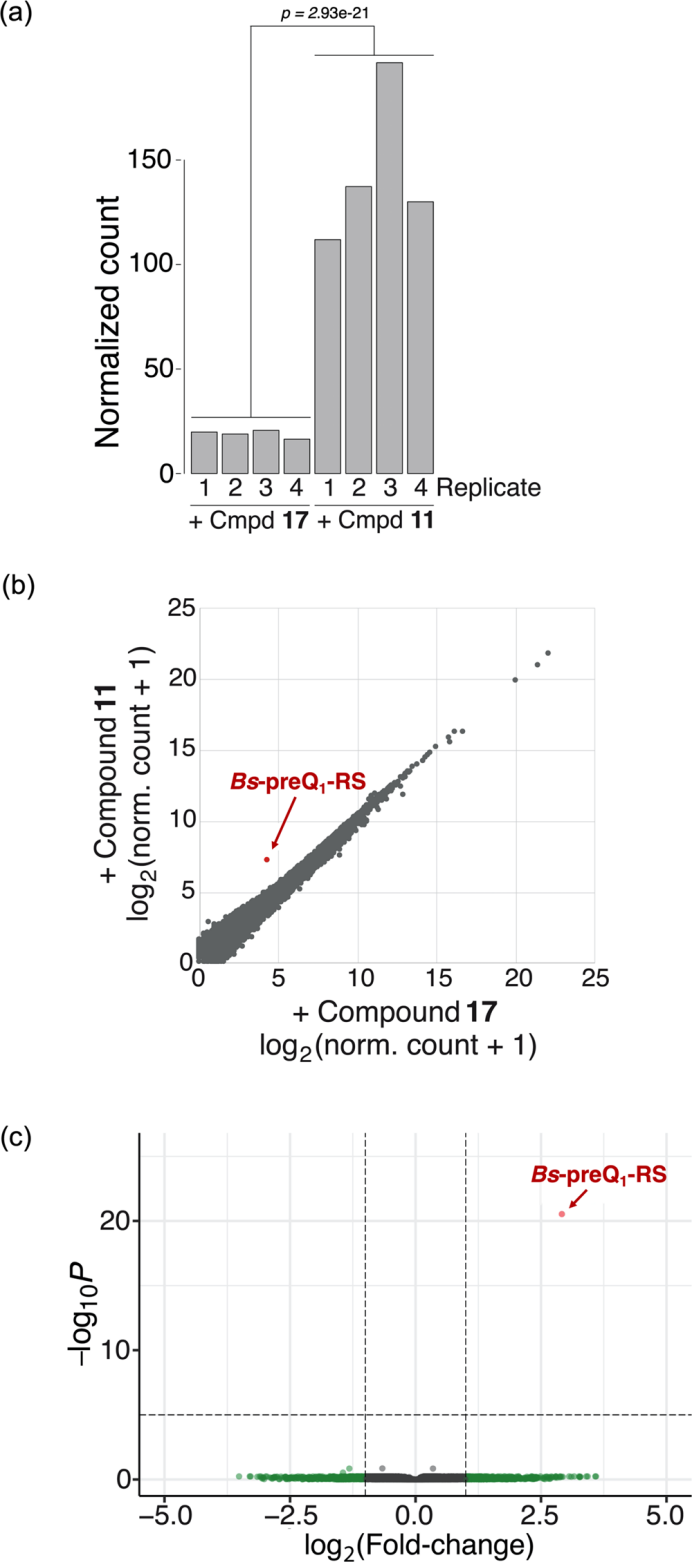
Selective enrichment of *Bs*-preQ_1_-RS aptamer by **11** in total human RNA with *Bs*-preQ_1_-RS aptamer spiked in. **a** Comparison of the normalized counts of the *Bs*-preQ_1_-RS aptamer across **11** and **17** treated samples shows significantly higher counts in all four replicates of **11** treated samples. Statistical significance was calculated by two-tailed *t*-test analysis. **b** Correlation plot showing differential gene enrichment for compounds **11** and **17**. The gene entry corresponding to the *Bs*-preQ_1_-RS is labeled in red. **c** Volcano plot from differential expression analysis between **11** and **17**. The gene entry corresponding to the *Bs*-preQ_1_-RS is labeled in red. *p* = adjusted *p*-value. All analyses performed on four independent replicate samples treated with **11** or **17**.

Chem-CLIP experiments were also performed to further probe the selectivity of **11** for the full-length *Bs*-preQ_1_-RS aptamer in the presence of MCF-7 derived total cellular RNA^36^. After UV crosslinking in the presence of **11**, total RNA was click labeled with biotin-PEG_3_-azide and enriched with streptavidin-conjugated magnetic beads. Enriched RNA was then reverse transcribed, and samples were submitted for next-generation sequencing. Compound **17**, which lacks the PreQ_1_ targeting warhead, was again used as a negative control. In samples where *Bs*-preQ_1_-RS was spiked into total RNA, differential gene expression analysis between **11** and **17** treated samples revealed that the aptamer sequence was significantly enriched by compound **11** (log_2_fold-change > 2.5, adjusted *p*-value = 2.93e^−21^ (Fig. 6a)). As evident in Fig. 6b and c, no other RNAs were significantly enriched when comparing **11** to **17** in this analysis. This high level of selectivity makes this compound-aptamer an ideal pair for chemical biology applications that require specific labeling in complex environments.

### Chem-CLIP and c-Chem-CLIP sequencing experiments to quantitatively assess selectivity of 11 in human transcriptomes

**Fig. 7:**
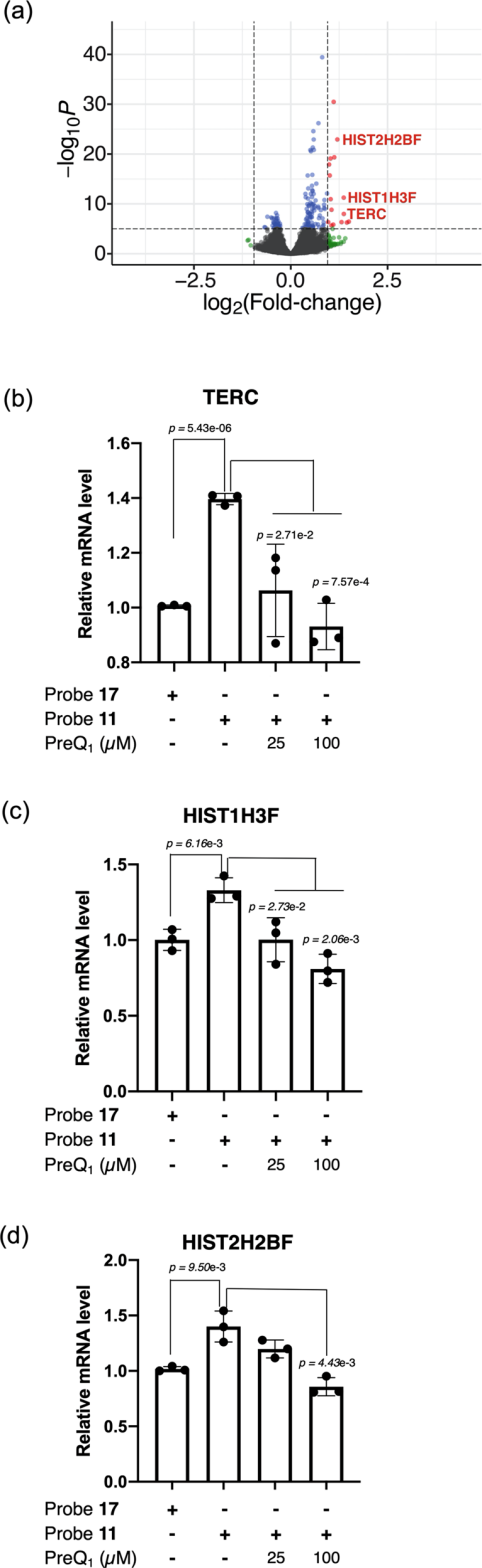
The enrichment of endogenous human transcripts with **11**. **a** Volcano plot of differential expression (DeSeq2) analysis between compound **11** and **17** treated samples (*n* = 4 for **11** and *n* = 3 for **17**). *p* = adjusted *p*-value. Histograms generated through RT-qPCR representing the relative level of **b** TERC, **c** HIST1H3F, and **d** HIST2H2BF in **11** and **17** treated samples. Data are presented as the mean ± SEM (*n* = 3) of three independent experiments. Statistical significance was calculated by two-tailed *t*-test analysis. Source data are provided as a Source Data file.

To further investigate crossreactivity of **11** with other human cellular RNAs, a Chem-CLIP experiment was also performed without exogenous *Bs*-preQ_1_-RS. In the absence of *Bs*-preQ_1_-RS, **11** selectively enriched 16 transcripts including TERC, HIST1H3F and HIST2H2BF (cutoff: log_2_fold-change > 0.95, −log_10_*P* > 4) (Fig. 7a and Supplementary Information S12). No genes were significantly depleted when the same cutoffs are applied. Interestingly, 14 out of 16 of the most significantly enriched hits belong to the histone gene family. Owing to the high level of sequence conservation between these genes, it is possible that their mRNA products share a common structure (i.e., an aptamer-like domain) that is specifically being recognized by **11**. For example, histone family mRNAs have a conserved, stable stem loop at their 3′ end in place of the typical polyadenylated tail that forms essential interactions with stem-loop proteins required for histone mRNA processing and translation^44,45^. The two other hits in this group, TERC and RMRP, both encode structured non-coding RNAs that form ribonucleoprotein complexes with the human telomerase catalytic subunit (hTERT)^46^. The lack of enrichment of these genes in the presence of *Bs*-preQ_1_-RS, which presumably sequesters the majority of **11**, suggests that these could represent specific interactions (Fig. 5a).

Competitive quantitative reverse transcription PCR (RT-qPCR) experiments (c-Chem-CLIP) were also performed with TERC, HIST1H2F and HIST2H2BF to validate individual enrichment events and assess biochemical competition with unlabeled probes^47^ (Fig. 7b–d). By RT-qPCR, TERC was enriched approximately 1.5-fold in **11** treated samples compared to the negative control (**17**), in good agreement with sequencing data. Furthermore, this enrichment was competable with free PreQ_1_ ligand, which lacks the diazirine-alkyne crosslinking warhead. Similar results were observed with HIST1H3F and HIST2H2BF. Enrichment was also observed for HIST2H2BF, however, competition with PreQ_1_ was only evident at the highest concentration of competitor (100 μM) (Fig. 7d). Together, the results of these sequencing and qPCR experiments indicate that these RNAs selectively bind to this ligand. Further investigation is underway to map these crosslinks and fully characterize these interactions. Extension of this c-Chem-CLIP crosslinking pull-down strategy to live cells in an effort to confirm the relevance of these potential interactions with **11** is currently underway.

## Discussion

Herein, we employ a structure-informed strategy to design and characterize covalent probes of the PreQ_1_ riboswitch aptamer. Initial attempts to design electrophilic probes were unsuccessful, however photocrosslinking probes reacted with efficiency approaching 50% modification. We applied multiple different biophysical and biochemical assays to assess the reactivity and site-specificity of the photoaffinity probe towards the target RNA in crosslinking experiments. Digestion of the crosslinked aptamer and analysis by mass spectrometry revealed that the photocrosslinker only reacts with guanine bases, with no crosslinks observed to the sugars. Given that there are only 3 conserved guanines in the class-I preQ_1_ aptamer (Bs, Tt and Ss), two of which are proximal to the binding site, this observation confirms a highly specific crosslinking event. The carbene-based reactivity typically associated with diazirines would be expected to predominate in stabilized diazirine probes such as compounds **14–17** and to alkylate multiple chemically diverse sites due to high reactivity^41,42^. However, here we observe unstabilized diazirines as having higher crosslinking efficiency and specific reactivity, suggesting that crosslinking may occur through an alkylation event via a rearranged diazo-intermediate (as observed elsewhere^48,49^). Based on the high reactivity of this intermediate it is likely that **11** can form adducts with multiple different atoms in multiple different nucleobases in the binding pocket, and more than one product is possible. However, we observed that mutation of G4 to adenosine resulted in a roughly 40% reduction in crosslinking, suggesting that this residue is the majority site of modification.

Importantly, binding studies, X-ray crystallography and transcription termination assays revealed that labeling PreQ_1_ with the crosslinking sidechain does not ablate binding or function and preserves the majority of the contacts that the compound makes with the aptamer. Despite a 50-fold reduction in affinity, the specificity of this interaction enables selective photocrosslinking of **11** to a PreQ_1_ riboswitch in cell lysates, which further illustrates the robustness of this approach to selectively target the RNA of interest under biologically relevant conditions. Thus, this PreQ_1_/aptamer pair may find substantial utility in chemical biology applications for targeting RNA due to the ease of manipulation of the PreQ_1_ scaffold and robustness of the proximity induced crosslinking. This straightforward approach relies exclusively on chemical recognition to achieve site-specific modification of an RNA. By fully characterizing this specific recognition event, we were additionally able to probe the transcriptome for potential PreQ_1_ binding sites on human RNAs. Through Chem-CLIP and competitive qPCR experiments in total cellular RNA, we were able to show that **11** crosslinked to the aptamer with exceptional selectivity. In the absence of aptamer, **11** selectively enriched 16 RNAs when compared to the negative control probe **17**. The high level of structural similarity between PreQ_1_ and guanine is particularly intriguing in this regard, as it is possible that guanine can also specifically interact with structures in these RNAs. In this way, evaluation of bacterial riboswitch ligands like PreQ_1_ can shed light on potential existing RNA aptamers for human metabolites.

Humans lack the biosynthetic machinery to produce PreQ_1_ and related metabolites such as queuine and queuosine, and they must be acquired from diet or commensal bacteria such as *B*. *subtilis*. However, they are important metabolites that specifically modify certain human tRNAs to promote protein folding^10,50,51^. Moreover, queuosine modification occurs at the wobble position of the tRNA with GUN anticodons and this modification regulates the translation efficiency^52^. The specific role that these metabolites play in humans remains under investigation, though there is at least some evidence that they may also have anti-cancer activity in addition to their regulatory roles in commensal bacteria^53,54^. Here, we demonstrate that **11** also has specific interactions with important regulatory human RNAs, including TERC, HIST1H3F, and HIST2H2BF, all of which have been shown previously to form stable RNA structures. 14 of the 16 enriched genes were for histone mRNAs, which is intriguing due to the previously reported shared, stable stem-loop motif in the 3’ UTR of histone mRNAs that plays an important regulatory role in their expression through interactions with stem-loop binding protein (SLBP)^45^. Additionally, the other two enriched genes, TERC and RMRP are both highly conserved short, structured non-coding RNAs that are known to play roles in disease pathogenesis^55–57^. The structure of vertebrate TERC has been studied extensively and this RNA has been shown to form multiple stable structures including a pseudoknot domain that is required for binding to hTERT^58^. Telomerase activity is low in the majority of healthy somatic cells, however, it is significantly increased in various cancers^55^, and TERC has been implicated as a potential therapeutic target in many of these cancers^59^. While the biological significance of the proposed interaction between **11** and TERC is currently unknown, small molecule probes that can selectively recognize this RNA would be valuable to researchers studying telomerase biology. Overall, the observation that simple metabolites have specific binding interactions with important regulatory RNAs such as these is intriguing and will require more in-depth investigation to evaluate potential regulatory roles.

This work highlights the importance of design considerations when generating chemical probes to crosslink RNA. We designed 16 probes, but only a small number crosslinked with sufficient efficiency to be broadly useful. Thus, care should be taken to design probes that avoid false negative events in photocrosslinking applications, particularly if measuring selectivity in a complex system is the goal. We find that unstabilized diazirine probes modify RNA with the highest efficiency, likely through an alkylation mechanism, and that linker structure has a high impact. Through in-depth biophysical analysis and Chem-CLIP studies, we demonstrate high selectivity between **11** and three class-I PreQ_1_ aptamers in cell lysates and total RNA, indicating potential uses for this system in chemical biology applications. Further, probe **11** targets both minimal aptamer domains and full riboswitches that include both transcriptional and translational expression platforms. Finally, mapping PreQ_1_ binding events in human transcriptomes revealed several specific binding events to regulatory RNAs. Further investigation of these and other interactions, including cell-based photocrosslinking approaches may prove useful to interrogate how related metabolites like guanine interact with human RNAs.

## Methods

### General information

Unless otherwise stated, all reagents and solvents were purchased from commercial vendors and used without further purification. All ^1^H, ^13^C, and ^19^F NMR spectra were collected on a 500, 126, and 471 MHz NMR spectrometers in (CD_3_)_2_SO, respectively. The ^1^H, ^13^C, and ^19^F NMR chemical shifts are given in parts per million (δ) with respect to an internal tetramethylsilane (TMS, *δ* = 0.00 ppm) standard and/or 3-chlorobenzotrifluoride (*δ* = −64.2 ppm relative to CFCl_3_). NMR data are processed, annotated, and reported in the following format: chemical shift (multiplicity (s = singlet, brs = broad singlet, d = doublet, t = triplet, q = quartet, m = multiplet), coupling constants (Hz), Integration) using MestReNova. The large-scale synthesis of 7-aminomethyl-7-deazagaunine (PreQ_1_) was carried out according to previously reported protocol^60^.

### Oligonucleotides

Deprotected and HPLC purified riboswitch aptamers or full-length riboswitch were purchased from Horizon Discovery Inc. or IDT with the following sequences and used without any further purifications.

***Tt*-preQ**_**1**_**-RS:** 5′-CUGGGUCGCAGUAACCCCAGUUAACAAAACAAG-3′

**Cy5-*Tt*-preQ**_**1**_**-RS**: 5′-Cy5-CUGGGUCGCAGUAACCCCAGUUAACAAAACAAG-3′

***Tt*-preQ**_**1**_**-RS(G4A):** 5′-CUGAGUCGCAGUAACCCCAGUUAACAAAACAAG-3′

***Tt*-preQ**_**1**_**-RS(G5A):** 5′-CUGGAUCGCAGUAACCCCAGUUAACAAAACAAG-3′

***Tt*-preQ**_**1**_**-RS(G11A):** 5′-CUGGGUCGCAAUAACCCCAGUUAACAAAACAAG-3′

***Tt*-preQ**_**1**_**-RS (ab 13_14**): 5′- CUGGGUCGCAGU-rab-rab-CCCCAGUUAACAAAACAAG-3′ (rab means abasic site)

***Bs*-preQ**_**1**_**-RS**: 5′-AGAGGUUCUAGCUACACCCUCUAUAAAAAACUAA-3′

**Cy5-*Bs*-preQ**_**1**_**-RS**: 5′-Cy5-AGAGGUUCUAGCUACACCCUCUAUAAAAAACUAA-3′

***Bs*-preQ**_**1**_**-RS full length (includes both the aptamer domain and the expression platform):** 5′-GCGGGAGAGGUUCUAGCUACACCCUCUAUAAAAAACUAGGACGAGCUGUAUCCUUGGAUACGGCCUUUU-3′

***Ss*-preQ**_**1**_**-RS:** 5′-AGAGGUUCCUAGCUGAUACCCUCUAUAAAAAACUA-3′

**Cy5-*Ss*-preQ**_**1**_**-RS**: 5′-Cy5-AGAGGUUCCUAGCUGAUACCCUCUAUAAAAAACUA-3′

**SAM-II-WT-RS**:5′-UCGCGCUGAUUUAACCGUAUUGCAAGCGCGUGAUAAAUGU AGCUAAAAAGGG

**miR21-hp**: 5′-GGGUUGACUGUUGAAUCUCAUGGCAACCC-3′

### Covalent crosslinking with electrophile containing compounds 1–10

The*Tt*-preQ_1_-RS aptamer (see above) was diluted to 10 µM in riboswitch buffer (50 mM Tris (pH 7.5), 100 mM KCl, 2 mM MgCl_2_) and was folded by heating at 75 °C in a heat block for 5 mins, and was allowed to cool to RT over the course of 1 h. For compound treatment, 10 µL of total volume was prepared by adding 9.5 µL of the refolded *Tt*-preQ_1_-RS to 0.5 µL of DMSO containing compounds **1–10** (500 µM final concentration, 50:1 molar ratio of crosslinking small molecule/RNA). The solutions were incubated at 40 °C for 2 h. After incubation, the samples were mixed with denaturing loading dye and heated at 95 °C for 5 min. Equal volumes of the samples were then subjected to 17% denaturing PAGE.

### UV photocrosslinking experiments

For the initial screening of compounds **11**–**16** binding to *Bs*-preQ_1_-RS aptamer, the *Bs*-preQ_1_-RS (5 µM, 50 µL) in riboswitch buffer (50 mM Tris (pH 7.5), 100 mM KCl, 2 mM MgCl_2_) was heated to 75 °C for 5 min and allowed to cool to room temperature over 1 h. After folding, the RNA was treated with compounds **11–16** (250 µM final concentration in 5% DMSO/riboswitch buffer) and incubated for 30 min in the dark at room temperature. Note that it is advised to keep the DMSO level at or below 5% in the reaction mixture. After pre-incubation, the reaction mixture was irradiated with 365 nm light in a SPECTROLINKER XL-1000 UV Crosslinker for 15 min. Multiple crosslinking methods were explored; however, optimal results were obtained by transferring the solution to a clear 96-well plate and placing the uncovered wells as close as possible to the UV light source in the photocrosslinker. Subsequently, the reaction mixture was analyzed by MALDI-TOF and/or denaturing PAGE.

For the dose-dependent crosslinking experiments, the *Ss*-preQ_1_-RS aptamer (5 μM) was folded in riboswitch buffer and incubated with varying concentration of probe **11** (25 μM, 50 μM, 100 μM, 150 μM, 200 μM, and 250 μM) at room temperature for 30 min. Then samples were UV photocrosslinked as described above and analyzed by MALDI-TOF and/or denaturing PAGE.

For the time-dependent crosslinking experiments, the *Ss*-preQ_1_-RS aptamer (5 μM) was folded in riboswitch buffer and incubated with probe **11 (**250 μM) at room temperature for 30 min. Then samples were UV crosslinked as described above but with different length exposure to UV light (2, 5, 10, and 15 min). After the UV crosslinking, samples were analyzed by MALDI-TOF and/or denaturing PAGE.

For the competition experiments with PreQ_1_ and tRNA, the *Ss*-preQ_1_-RS aptamer (5 μM) was folded in riboswitch buffer and incubated with probe **11** (250 μM) at room temperature for 30 min. For competition experiments with PreQ_1_, samples were prepared with free PreQ_1_ ligand with increasing concentration from 125 µM to 1250 µM and added into the solution containing compound **11** and the *Ss*-preQ_1_-RS to allow for competition before UV crosslinking. In the case of tRNA competition, samples were prepared with tRNA with increasing concentration from 2.5 µM to 40 µM concentration and titrated into the solution containing compound **11** and the *Ss*-preQ_1_-RS allow for competition before UV crosslinking. Samples were UV crosslinked for 15 min. After UV crosslinking, samples were analyzed by MALDI-TOF and/or denaturing PAGE.

Photocrosslinking experiments for other RNAs such as SAM-II and miR-21 were performed as described above. The molar ratio between the probe **11** and SAM-II or miR-21 was maintained at 50:1 ratio and samples were UV crosslinked for 15 min. Then samples were analyzed by PAGE.

For photocrosslinking experiments of **11** to *Tt*-preQ_1_-RS mutants, the same protocol was used as described above. 10 µM of *Tt*-preQ_1_-RS wild type or mutants were folded in riboswitch buffer and incubated with probe **11** (500 μM) at room temperature for 30 min. Samples were then UV crosslinked for 15 min. After photocrosslinking, samples were analyzed by PAGE.

### Denaturing PAGE analysis (general procedure)

For PAGE analysis, RNA samples were mixed with equal volume of denaturing loading dye and heated at 95 °C for 5 min. Equal volumes of the samples were then subjected to 17% PAGE in the presence of 7 M urea. The gel was stained in 1X SYBR® Gold Nucleic Acid Gel Stain (Invitrogen) for 15 min at room temperature. The gel images were obtained by scanning the gel on an AMERSHAM TYPHOON Biomolecular Imager. Image J software was used for processing. Crosslinking efficiency was calculated by quantifying the higher molecular weight band intensity (crosslink adduct band). Values reporterd are the average of three replicates.

### Orbitrap-LC/MS analysis and digestion

The *Ss*-PreQ1-RS aptamer (50 µM, 100 µL) was folded in riboswitch buffer by heating at 75 °C for 5 min and followed by cooling to room temperature over 1 h. Then the folded RNA was mixed with probe **11** (500 µM) and incubated for 30 min in the dark at room temperature. After pre-incubation, the reaction mixture was UV photocrosslinked for 15 min as describe above. Then sample mixtures (contains a mixture of photocrosslinked and unmodified RNA) were assessed for extent of crosslinking by negative ion MALDI operated in linear mode over a 5–20 kDa range. Next, the mixtures were analyzed by negative ion electrospray ionization (ESI)-LC/MS on an LTQ-Orbitrap-XL LC/MSn system at 30,000 resolutions. High-resolution deconvolution of oligonucleotide anion charge-states was carried out using the Xtract program^61^. Crosslinked RNA was subjected to enzymatic digestion to generate individual nucleosides and was analyzed by LC/MSn to determine where crosslinks were occurring and to identify possible structures. For the enzymatic digestion study, photocrosslinked RNA was transferred to a PCR tube and treated with NEB Nucleoside Digestion Mix® Reaction Buffer (10 µL, 10x) and Nucleoside Digestion Mix (5 µL) and incubated at 37 °C for 1 h. Then, the reaction mixture was analyzed by MS and MS/MS using both CID and HCD. A narrow-bore C18 column system, eluted with CH_3_OH/H_2_O gradients tailored to the compounds of interest, was used for separations. High-resolution CID and HCD MS/MS of the guanosine adduct MH + indicated that adduction occurred exclusively on the guanine base.

### Click reaction on photocrosslinked RNA for labeling with fluorophores and biotin-azides

Biotin or fluorophore-azide probes were crosslinked to aptamer RNA according to an established copper catalyzed click chemistry protocol^62^. A solution of each 10 μM *Ss*-preQ_1_-RS aptamer was folded in the riboswitch buffer. Then the aptamers were incubated with 500 μM of Probe **11** and photocrosslinked as described above. Note that this crude solution contains a mixture of photocrosslinked and unmodified RNA (approximately 1:1.5 photocrosslinked to unmodified). To the above solution, either Biotin-PEG_3_-azide or Cy5-picolyl-azide (10 µL of 10 mM solution in DMSO), THPTA/CuSO_4_ (25 µL of premixed solution of THPTA and CuSO_4_ for 10 min in 2:1 ratio using 200 mM and 100 mM solutions in H_2_O, respectively), and L-Sodium ascorbate (7.5 µL of 100 mM in H_2_O) were added and the reaction mixture was stirred for 1 h. The resulting adducts were analyzed by MALDI-TOF and denaturing PAGE.

### Single-round transcription termination assay

The transcription termination assays were carried out as previously reported^29^. The DNA plasmid containing λPR promoter and 26-nt C-less sequence followed by the *Staphylococcus saprophyticus (Ss)* preQ_1_ riboswitch and its downstream sequence cloned into pIDTSMART-AMP was purchased from Integrated DNA Technologies. The DNA template was amplified by PCR from the plasmid using forward and reverse primers, and then was gel-extracted after agarose gel electrophoresis for purification. Halted transcription complexes were prepared in a solution containing 1 µM GTP, 5 µM ATP, 5 µM UTP, 100 µM ApU, [α-^32^P] GTP, 75 nM DNA template, 0.0167 U/µL *Escherichia coli* RNA polymerase holoenzyme (New England BioLabs) in 1 × transcription buffer (20 mM Tris-HCl, pH 8.0, 2 mM NaCl, 1 mM MgCl_2_, 4% glycerol, 0.1 mM DTT, and 0.1 mM EDTA), and incubated at 37 °C for 15 min. A DNA oligonucleotide complementary to the 26-nt C-less sequence was added to the reactions at 1.1 µM final concentration in 1 × transcription buffer and incubated at room temperature for 5 min, in order to prevent undesired non-specific interactions between the 26-nt C-less sequence and riboswitch. Elongation was restarted by combining 9 µL of halted transcription complex, 3 µL of the compound either PreQ_1_ or probe **11** (0–5 mM compound and 25% DMSO in 1 × transcription buffer), and 3 µL of NTPs mix (200 µM ATP, 200 µM CTP, 200 µM GTP, 200 µM UTP, 100 µg/mL heparin, and 250 mM KCl in 1 × transcription buffer), and incubated at 37 °C for 20 min. To remove the DNA template, 0.5 U of RQ1 RNase-Free DNase (Promega) was added to the reactions and incubated at 37 °C for 10 min. The reactions were stopped by adding equal volume of loading dye (8 M urea, 20% sucrose, 0.05% bromophenol blue, and 0.05% xylene cyanol in 2 × TBE). The reaction mixture was separated by 8% denaturing PAGE and visualized by phosphorimager. Full gel images are provided as Supplementary Figures. The band intensity was analyzed by ImageQuant software (GE Healthcare). Termination efficiency was calculated with dividing the intensity for the terminated RNA band by those for the total (terminated and antiterminated) RNAs.

### Microscale thermophoresis (MST)-binding measurements for PreQ_1_ and 11

Fluorescently labeled *Bs*-preQ_1_-RS aptamer and *Tt*-preQ_1_-RS aptamer were prepared in 50 mM Tris-HCl (pH 7.5), 100 mM KCl, and 2 mM MgCl_2_. Both preQ_1_riboswitch aptamers were annealed by heating to 75 °C for 5 min and then cooled to room temperature over 1 h. A twofold dilution series of PreQ_1_ or probe **11** was prepared in 100 nM Cy5-labeled aptamer and 5% DMSO, with the final concentrations of PreQ_1_ or **11** ranging from 500 *μ*M to 0.0153 μM. Samples were incubated at room temperature for at least 15 min. Following incubation, the samples were added to premium coated capillaries (NanoTemper Technologies) and subsequently subjected to MST analysis. MST experiments were conducted in triplicate on a Monolith NT.115 system (NanoTemper Technologies). The results were analyzed by TJump analysis, and the values obtained were normalized and plotted against the PreQ_1_ or **11** concentrations. The apparent dissociation constant was then determined using a single-site model to fit the curve.

### X-ray crystallography experiments and data collection

For X-ray crystallography, we used the preQ_1_ riboswitch aptamer domain from *Thermoanaerobacter tengcongensis* (Tt). The wild-type aptamer and abasic mutant (ab13_14), in which the nucleobases at positions 13 and 14 were removed, were employed for crystallization. The crystals of *Tt*-preQ_1_-RS aptamer domain in complex with **11** were prepared at 20 °C and cryoprotected by the same methods as reported previously^4^. X-ray diffraction data were collected at the beamlines BL32XU and BL45XU of the SPring-8 (Hyogo, Japan) with the aid of an automatic data-collection system ZOO^63^ and BL-1A of the Photon Factory (Ibaraki, Japan). Diffraction data were integrated and scaled with the programs KAMO^64^ and XDS^65^. Data processing statistics are summarized in Table S1.

### Structure determination and refinement

The co-crystal structures were solved by the molecular replacement method with the program PHASER^66^, using the previously determined *Tt*-preQ_1_-RS aptamer domain structure (PDB ID: 6E1W) as the search model. The solutions were subjected to simulated annealing to uncouple the working and free *R* values in the refinement process, as well as energy minimization, restrained isotropic B-factor, and TLS refinement with PHENIX^67^. The resulting electron density maps revealed the locations of the compound. The atomic models were then improved by iterative cycles of refinement with PHENIX and manual rebuilding with COOT^68^. The current co-crystal structures of ab13_14 and the wild-type *Tt*-preQ_1_-RS aptamer domain in complex with **11** were refined to an *R*_work_/*R*_free_ of 0.190/0.208 and 0.193/0.235 at 1.57 and 2.80 Å resolution, respectively.

### Cell culture and RNA isolation

MCF-7 cells were grown in T75 flasks in Dulbecco’s modified Eagle’s medium (DMEM) with high glucose supplemented with 10% fetal bovine serum and 1% antibiotics (streptomycin and penicillin) at 37 °C in 5% CO_2_ in a humidified incubator according to ATCC’s recommendations. On the day of the experiment (~80% confluency) the cells were harvested by trypsinization, washed and resuspended in 1x PBS. Total RNA was isolated from MCF-7 cells using the RNeasy mini kit (Qiagen, Cat #: 74104) with on column DNAse digestion according to the manufacture protocol. The concentration of eluted RNA was measured using a nanodrop.

### Photocrosslinking experiments with biotinylated probe 11 (Bio-11) in the presence of cell lysates

Cell lysates were extracted from the MCF-7 cells using RIPA buffer (Santa Cruz). Then 1 µM of folded *Ss*-preQ_1_-RS aptamer (34 nucleotide length, see above) was added into the 5 µg of cell lysate and photocrosslinked to Bio-**11** (250 µM) as described above. Next, equal volumes of the samples were subjected to 17% denaturing PAGE in the presence of 7 M urea. Meanwhile, pre-cut nylon membrane, whatman filter paper and sponge were soaked in 0.5x TBE buffer. Once the gel was run, the transfer sandwich was prepared, and the sandwich was placed into the BioRad Trans-Blot Cell and filled with pre-chilled 0.5x TBE. The Trans-Blot Cell was run at 80 V for 60 min at 4 °C with continuous stirring. Then the membrane was UV crosslinked at 1200 mJ for 3 min in an UVP crosslinker. Then the membrane was transferred into blocking buffer (ThermoFisher TM pierce starting blocking buffer at room temperature for 1 h in the Shaker. Finally, the membrane was incubated with HRP-conjugated Streptavidin (ThermoFisher N100) in the 1:2000 dilution at room temperature for 2 h and then the biotinylated probe conjugated band was visualized by Western Blotting Luminol Reagent (Cell signaling Technology #7003) in the ChemiDoc imager.

### Photocrosslinking experiments with 11 in the presence of total cellular RNA

Total RNA isolated from the MCF-7 cells was split into different tubes (5 µg RNA/condition) and was diluted up to 50 µL total volume with riboswitch buffer (50 mM Tris (pH 7.5), 100 mM KCl and 2 mM MgCl_2_). One micromolar of the full-length *Bs*-preQ_1_-RS (70 nts) was added to the appropriate tubes during the dilution with riboswitch buffer. Before the addition, RNA was folded as described above, by heating at 75 °C for 5 min and allowing to cool to RT over the course of 1 h. Next, 20 mM compounds **11** and **17** were prepared in DMSO and the compounds were diluted to a final concentration of 250 µM with the RNA in riboswitch buffer (5% final DMSO). Compounds **11** or **17** were mixed with RNA samples and incubated in the dark for 30 min to establish an equilibrium. After incubation, the solutions were moved to a clear plastic 96-well microtiter plate (VWR) for UV crosslinking. The control sample (Fig. 6, lane 7), that would not be irradiated, was left in the dark in the original tube. In the case of competition experiments, samples were prepared with the free PreQ_1_ ligand (500 µM and 1000 µM concentrations) titrated into the solution containing compound **11** and the total RNA to allow for competition before UV crosslinking. Samples in the plate were irradiated for 15 min at 365 nm using a SPECTROLINKER XL-1000 UV Crosslinker. After UV irradiation the sample mixtures were conjugated to TAMRA azide using the click labeling protocols described above. Finally, RNA was ethanol precipitated after TAMRA conjugation and samples were analyzed by PAGE.

### RNA sequencing experiments

Total RNA ((5 µg) isolated from MCF-7 cells was diluted up to 50 µL total volume with riboswitch buffer (50 mM Tris (pH 7.5), 100 mM KCl and 2 mM MgCl_2_). One micromolar of the full-length *Bs*-preQ_1_-RS (70 nts) was added into the tubes during the dilution with riboswitch buffer. Before the addition, *Bs*-preQ_1_-RS was folded as described above, by heating at 75 °C for 5 min and allowing to cool to RT over the course of 1 h. 20 mM stock solutions of compounds **11** and **17** were prepared in DMSO and the compounds were diluted to a final concentration of 250 µM with the RNA in riboswitch buffer (5% final concentration of DMSO). The compound and RNA mixtures were incubated at room temperature for 1 h and samples were UV crosslinked as described above. The modified RNA was labeled with biotin-PEG_3_-azide using the click protocol described above. After labeling the RNA was subjected to ethanol precipitation to remove excess biotin-PEG_3_-azide. The RNA pellets were dissolved in 50 µL of incubation buffer (50 mM Tris pH 7.5, 100 mM KCl, 0.01% Tween-20) and 50 µL of streptavidin linked magnetic beads (NEB) was added to each tube. After 30 min, the beads were pulled down on a magnetic tower and washed three times with 500 µL incubation buffer. Finally, 50 µL of elution buffer (95% formamide/H_2_O, 10 mM EDTA) was added to each tube and the tubes were placed on a 95 °C heat block for 4 min. The supernatant containing the eluted RNA was removed on the magnetic tower and the solution was diluted with RNAse free water and ethanol precipitated overnight. The concentration of the reconstituted RNA was measured using a Qubit RNA HS Assay Kit (Invitrogen). Two-hundred nanograms of each sample was subsequently used to prepare sequencing libraries with the QiaSeq Stranded Total RNA Library Kit (Qiagen). Completed libraries were submitted to Novogene for sequencing on a HiSeq 2500 (2 × 150 reads). Four replicates of each treatment condition were prepared and sequenced. We followed the same protocol as describe above but without addition of the *Bs*-preQ_1_-RS aptamer to assess crosslinking to other RNAs in MCF7 total RNA. All sequencing experiments were performed with four independent replicates for each condition (*n* = 4). In experiments without aptamer spike-in one replicate of **17** failed QC and was not included in this analysis.

### Sequencing data analysis

For the analysis of these pulldown experiments, a reference was built by using the longest isoform for every human RefSeq transcript, plus the sequence of the *Bs*-preQ_1_-RS full-length aptamer (see above). Reads were aligned to this reference using Bowtie2 and the “--very-sensitive-local” preset^69^. Resulting BAM files were then processed to TSV count files, containing the number of reads mapping to each transcript. The DESeq2 package^70^ (adjusted *p*-value < 0.05) was used to identify transcripts significantly enriched by compound **11** in the pulldown with respect to the control (compound **17**).

For the sequencing experiments without the aptamer spiked-in, the volcano plot generated by the DESeq2 package was used to identify potential hits that were significantly enriched (FC > 1.5-fold, adjusted *p*-value < 0.05 when comparing **11** treated samples to **17** treated samples).

### Quantitative reverse transcription PCR (RT-qPCR)

Samples for these experiments were prepared using the same protocol as described above for the RNA-sequencing experiments (without spiking *Bs*-preQ_1_-RS aptamer). However, in this case extra samples were prepared with the free PreQ_1_ ligand (25 µM and 100 µM concentrations) titrated into the solution containing compound **11** and the total RNA to allow for competition before UV crosslinking. RNA concentration was determined by using a QuBit RNA HS Assay Kit (Invitrogen), then 200 ng of RNA was used for cDNA synthesis with qScript cDNA SuperMix (Quanta Biosciences) according to the manufacturer’s protocol. The 20 µL reactions were incubated in a thermocycler (Bio-rad) for 5 min at 22 °C, 30 min at 42 °C, 5 min at 85 °C, then held at 4 °C. Then 20 ng of cDNA was subjected to qPCR using a Perfecta SYBR Green Super Mix (Quanta Biosciences) on an Eppendorf Mastercycler RealPlex2 in the presence of the appropriate set of primers. The reactions were incubated at 95 °C for 10 min, followed by 40 cycles of 95 °C for 30 s, 55 °C for 30 s and 72 °C for 20 s. Threshold cycles (C_T_) is defined as the first cycle that showed a detectable increase in fluorescence due to the formation of PCR products and was used to determine the template amount in each sample. The relative fold change in expression was measured using the Livak method^71^ and were normalized to the relative level of RNAs of the control experiment (compound **17**). For example, to calculate the ΔΔ(*C*_T_) between each gene of interest and the average of the control samples: Δ(*C*_T_) = *C*_T_ (target gene) − *C*_T_(Gapdh); ΔΔ(*C*_T_) = Δ*C*_T_ (treatment or knockdown) – Δ*C*_T_ (control); fold change = 2^–ΔΔ (*C*^_T_^)^. The primers used in these experiments are shown in the Supplementary Information Table S2.

### Reporting summary

Further information on research design is available in the Nature Research Reporting Summary linked to this article.

## Supplementary information

Supplementary Information

Reporting Summary

## Data Availability

Atomic coordinates and the structure factors of the co-crystal structures of ab13_14 and the wild-type *Tt*-preQ_1_-RS aptamer domain in complex with **11** have been deposited in the Protein Data Bank, under the accession codes 7E9E and 7E9I, respectively. Previously published crystals 3Q50, 6E1W and 3B1V were accessed from the PDB and used for reference. Raw fastq files along with processed alignment files and counts files from these sequencing experiments have been deposited on the NCBI’s gene expression omnibus (GEO) database. These files can be accessed with the following GEO accession code: **GSE168353**. The source data underlying Fig. 2A–E, 4C–D, 5A–B, 7B–D and Supplementary Figs. S1, S2, S5, S6A-B, S7A–D and S10 are provided as a Source Data file. All other relevant data are available from the authors upon request Source data are provided with this paper.

## Source data

Source data
