# Supplementary material for: A chemical probe based on the PreQ1 metabolite enables transcriptome-wide mapping of binding sites
Source: Nat Commun. 2021 Oct 6;12:5856. doi: 10.1038/s41467-021-25973-x (PMC8494917; doi:10.1038/s41467-021-25973-x)
Supplement: Supplementary file 1 — Supplementary Information [file 41467_2021_25973_MOESM1_ESM.pdf]

A Chemical Probe Based on the PreQ$_1$ Metabolite Enables Transcriptome-wide Mapping of Binding Sites

Sumirtha Balaratnam[1*], Curran Rhodes[1*], Desta Doro Bume[1], Colleen Connelly[1], Christopher C. Lai[1], James A. Kelley[1], Kamyar Yazdani[1], Philip J. Homan[2,3], Danny Incarnato[4], Tomoyuki Numata[5,6] and John S. Schneekloth Jr*[1,†]

Supporting Information

Section 1: Tables and Figures

Table S1: Summary of data collection and refinement statistics

Table S1. Summary of data collection and refinement statistics.

	ab13_14-**11**	Wild type-**11**
**Data collection**		
Space group	$P6_122$	$P6_322$
Cell dimensions		
a, b, c (Å)	52.7, 52.7, 177.0	113.2, 113.2, 59.5
Wavelength (Å)	1.0	1.0
Resolution (Å)	45.6-1.57 (1.62-1.57)	41.0-2.80 (2.98-2.80)
R_{merge}[a]	0.123 (2.22)	0.177 (1.92)
$R_{p.i.m.}$[b]	0.028 (0.506)	0.061 (0.655)
$CC_{1/2}$[c]	0.999 (0.641)	0.997 (0.663)
$<I>/<\sigma I>$	16.2 (1.3)	9.4 (1.3)
Completeness (%)	100 (100)	98.1 (96.0)
Redundancy	20.5 (20.1)	8.2 (8.5)
**Refinement**		
Resolution (Å)	45.6-1.57	41.0-2.80
No. reflections	21,395	5,768
R_{work}[d]/R_{free}[e]	0.190/0.208	0.193/0.235
No. atoms		
RNA	694	686
Ligand	22	22
Ions	13	5
Water	86	2
B-factors (Å^2)		
RNA	40.2	87.1

Ligand	37.5	97.7
Ions	110.3	147.0
Water	46.3	61.1
R.m.s. deviations		
Bond lengths (Å)	0.004	0.006
Bond angles (º)	0.900	1.175
PDB ID	7E9E	7E9I

The values in parentheses are for the outermost shell.

[a] $R_{merge} = \Sigma_{hkl} \Sigma_i |I_i(hkl) - \langle I(hkl)\rangle| / \Sigma_{hkl} \Sigma_i I_i(hkl)$, where $I_i(hkl)$ is the observed intensity and $\langle I(hkl)\rangle$ is the average intensity over symmetry-equivalent measurements.

[b] Definition of $R_{p.i.m.}$ can be found in Ref. 1.

[c] Pearson correlation coefficient between intensities of random half-dataset (Ref. 2).

[d] $R_{work} = \Sigma|F_o - F_c|/\Sigma F_o$ for reflections of working set.

[e] $R_{free} = \Sigma|F_o - F_c|/\Sigma F_o$ for reflections of test set (5.0% of total reflections).

Table S2: Sequences of primers used in qPCR experiments.

Gene name		Sequence (5’-3’)
RF00024 (ENSG00000277925.1)	Forward Primers	AAC CCT AAC TGA GAA GGG CG
	Reverse Primers	AGA ATG AAC GGT GGA AGG CG
HIST2H2BF (ENSG00000203814.6)	Forward Primers	TTC GCG CAA AAA TGC CG
	Reverse Primers	CTT CAG CAC CTT GTA CAC GTA A
HIST1H3F (ENSG00000277775.1)	Forward Primers	CAG CTC GTA AGT CCA CTG GC\
	Reverse Primers	CGA TTT CTG ATA GCG GCG GA
GAPDH (ENSG00000111640.14)	Forward Primers	AGG TCG GTG TGA ACG GAT TTG
	Reverse Primers	GGG GTC GTT GAT GGC AAC A

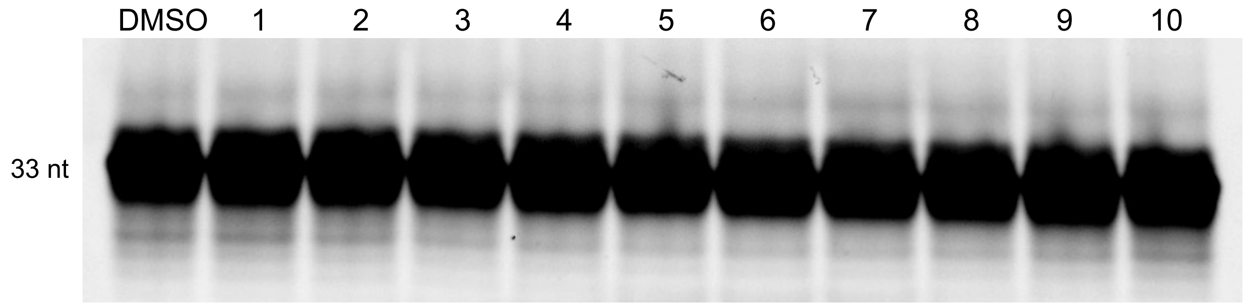

Figure S1: PAGE analysis of electrophilic probes **1-10** reactivity towards the *Tt*-preQ$_1$-RS. No evidence of higher molecular weight covalent adducts was observed in this case for any of the 10 compounds tested. For these experiments, the molar ratio between the *Tt*-preQ$_1$-RS aptamer and electrophilic probes was 1:50. The experiment was repeated three time independently. Full image was provided in the Source Data file.

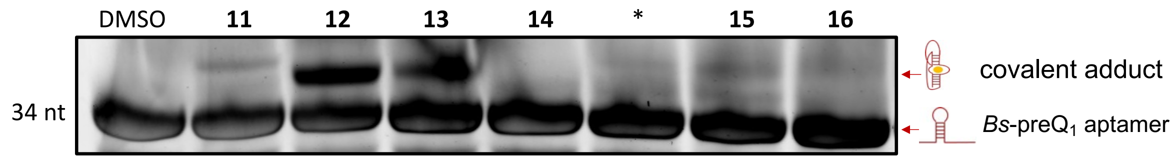

Figure S2: PAGE analysis of photoaffinity probes reactivity towards to *Bs*-preQ$_1$-RS aptamer. The unstabilized probes (**11**, **12**, and **13**) and stabilized probes (**15** and **16**) expect **14** showed formation the higher molecular weight covalent adduct. The molar ratio between the aptamer and probe was 1:50 under this experimental condition. The experiment was repeated three time independently and source data are provided as a Source Data file.

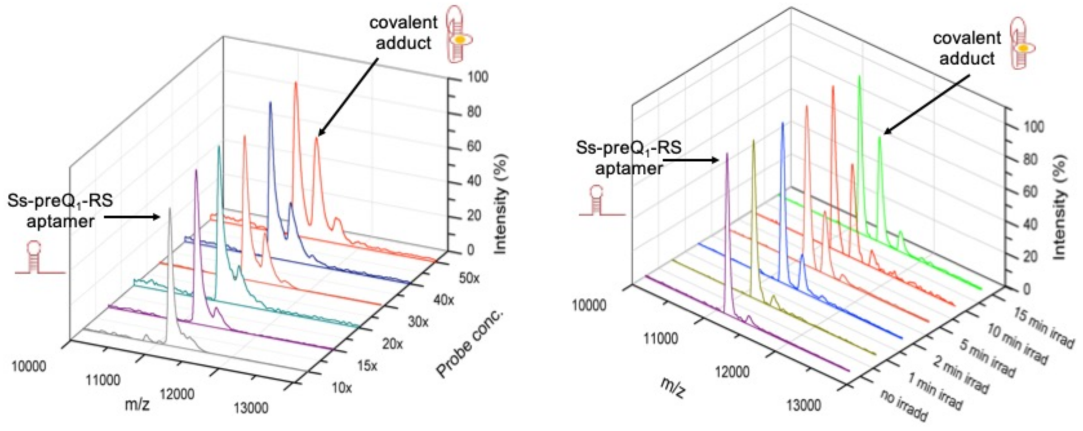

Figure S3: Orbitrap-LC/MS based characterization of dose (left) and time (right) dependent crosslinking efficiency of compound **11** towards *Ss*-preQ$_1$-RS (5 μM). In the dose dependent experiments, the "x" represents the molar ratio between probe and *Ss*-preQ$_1$-RS aptamer. The molar ratio between the aptamer and probe **11** was 1:50 in the time dependent crosslinking experiments. At molar ratios of 50x and above and/or with extended UV irradiation time, a second peak corresponding to a higher mass species consistent with a secondary covalent crosslinking event can be observed.

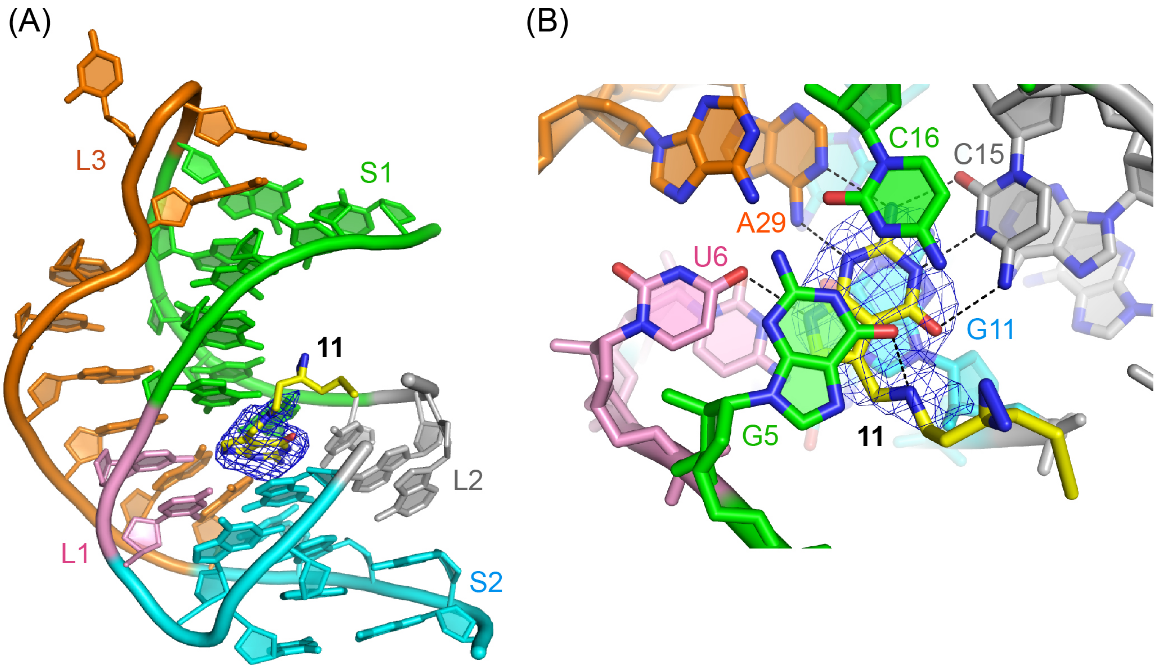

Figure S4: Crystal structures of the wild-type *Tt*-preQ$_1$-RS riboswitch aptamer complexed with **11**. **(A)** Overall structure of the complex. **(B)** Close up view of the binding site of **11**. S1, S2, L1, L2, and L3 are colored green, cyan, pink, gray, and orange, respectively. The mF_o – DF_c electron density map for **11** is colored blue and contoured at 3.0 σ. The nucleotides interact with the compound are labeled, and hydrogen bonds are indicated as dotted lines.

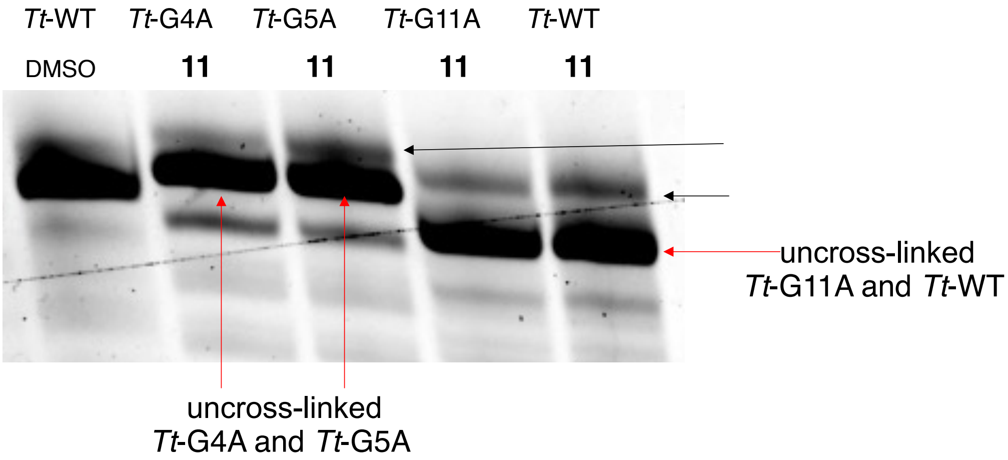

Figure S5: Representative PAGE gel image showing photocrosslinking of **11** to the WT and 3 different mutants of *Tt*-preQ$_1$-RS aptamers. The molar ratio between the aptamer and probe was 1:50 under this experimental condition. The experiment was repeated three time independently. Source data are provided as a Source Data file.

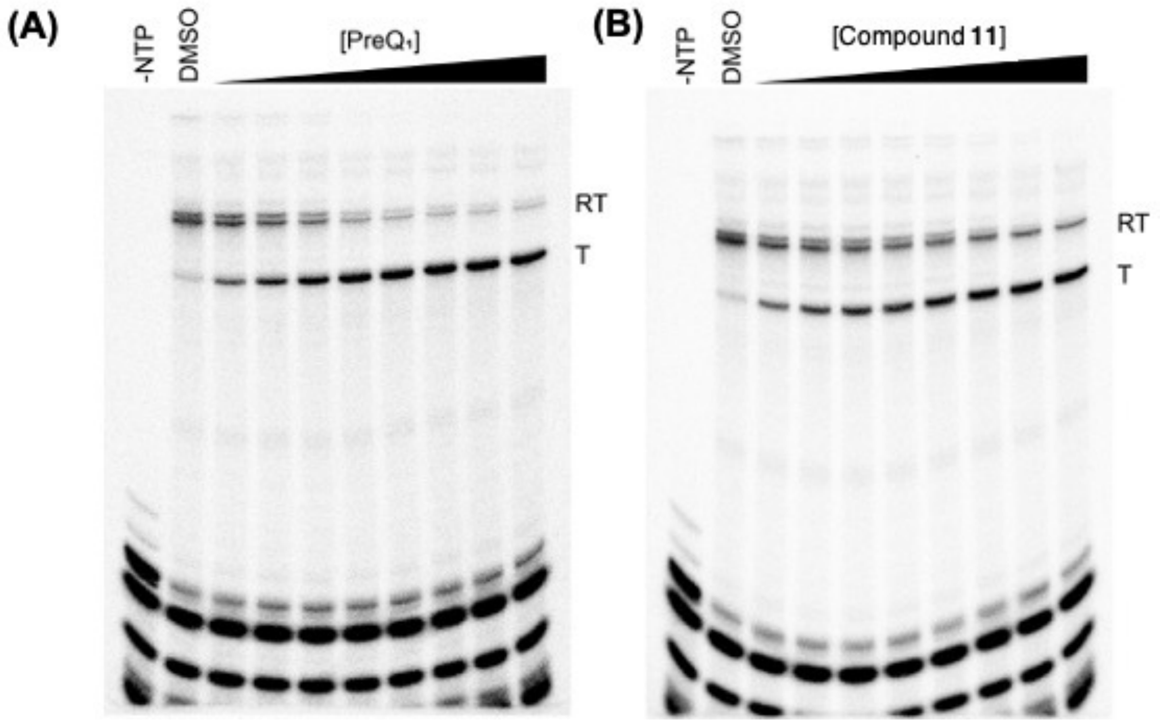

Figure S6: Representative PAGE gel images of the ^{32}P-labeled RNA products of in vitro transcription of the *Ss*-preQ1-RS template in the presence of increasing concentrations of (A) PreQ1 and (B) 11. Bands corresponding to the read-through transcription product (RT) and terminated transcription product (T) are indicated. Each experiment was repeated three time independently. Source data are provided as a Source Data file.

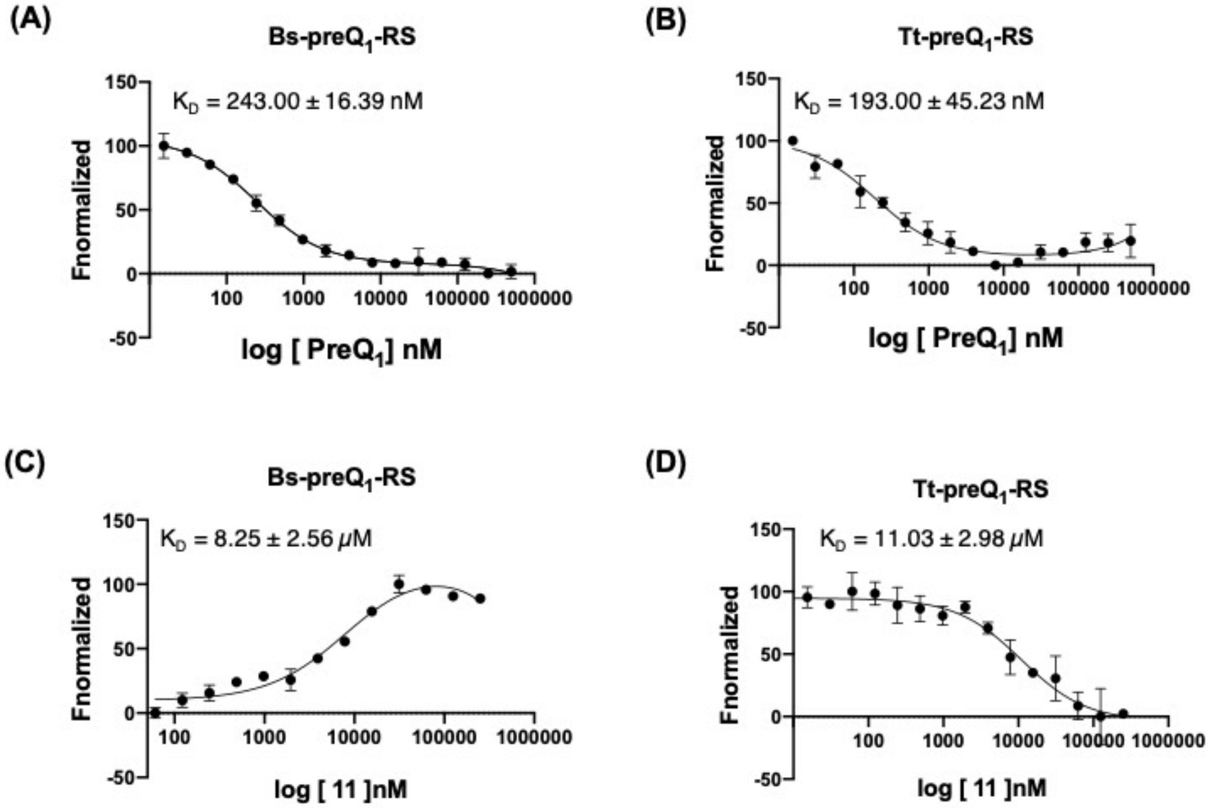

Figure S7: Binding curves generated by Microscale thermophoresis (MST) for binding of (A) PreQ$_1$ to the *Bs*-preQ$_1$-RS aptamer, (B) **11** to the *Bs*-preQ$_1$-RS aptamer, (C) PreQ$_1$ to the *Tt*-preQ$_1$-RS aptamer and (D) **11** to the *Tt*-preQ$_1$-RS aptamer. The opposite change in thermophoretic behavior suggest that aptamer undergo different conformation change upon binding to **11**. The binding constant was calculated by fitting the data in GraphPad Prism using a single-site mode of binding. The data are presented as the mean ± SEM (n=3) of three independent experiments. Each experiment was repeated three time independently. Source data are provided as a Source Data file.

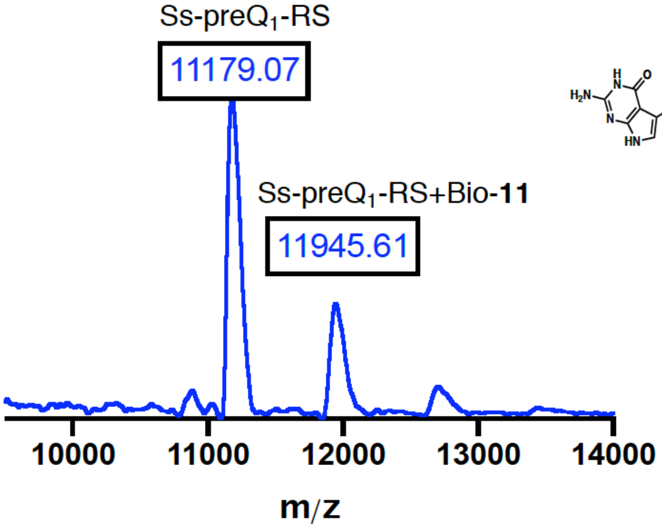

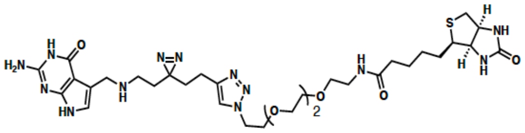

Bio-**11**
MW = 699.84 g/mol

Figure S8: MALDI-TOF analysis of reactivity of Bio-**11** towards the *Ss*-preQ$_1$-RS aptamer. The appearance of the higher molecular weight species after UV irradiation confirms the formation of a covalent adduct between Bio-**11** (500 μM) and the *Ss*-preQ$_1$-RS (10 μM). While the specific identify of this adduct is unknown (multiple products will be formed upon UV irradiation) the observed mass is consistent with the addition of 1 equivalent of Bio-**11** to the *Ss*-preQ$_1$-RS.

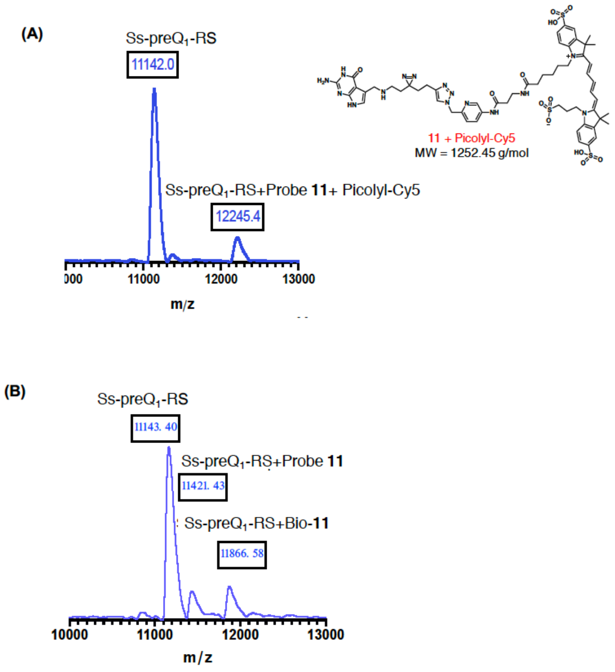

Figure S9: MALDI-TOF analysis of photocrosslinking and click catalyzed addition of **(A)** Cy5-picolyl-azide probe **(B)** biotin-(PEG)3-azide towards the *Ss*-preQ1-RS. In these experiments, the *Ss*-preQ1-RS (10 μM) was first treated with 500 μM of **11**. After incubation the sample was UV irradiated to crosslink **11** to the *Ss*-preQ1-RS. Finally, this mixture was treated with Cy5-picolyl-azide **(A)** and biotin-(PEG)3-azide **(B)** using the copper catalyzed click protocol described above. The higher molecular weight species indicated here is consistent with potential covalent adducts between the **11**-crosslinked-*Ss*-preQ1-RS and **(A)** Cy5-picolyl azide or **(B)** biotin-(PEG)2-azide. A peak corresponding to the adduct of *Ss*-preQ1-RS and **11** (no Bio-**11** click product) is visible in panel B due to incomplete reaction with the biotin-(PEG)3-azide under these conditions.

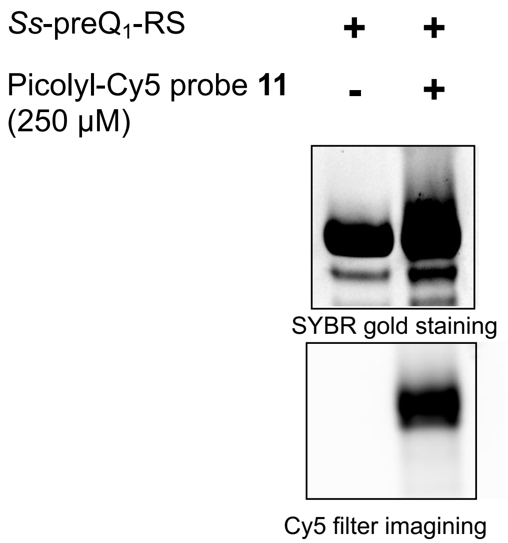

Figure S10: PAGE characterization of Cy5-picolyl probe cross-linked *Ss*-preQ$_1$-RS by SYBR gold staining (top) and Cy5 filter imaging (bottom). The molar ratio between the *Ss*-PreQ$_1$-RS (10μM) and Cy5-picolyl probe **11** (500 μM) is 1:50. The experiment was repeated three time independently and full image was provided in the Source Data file.

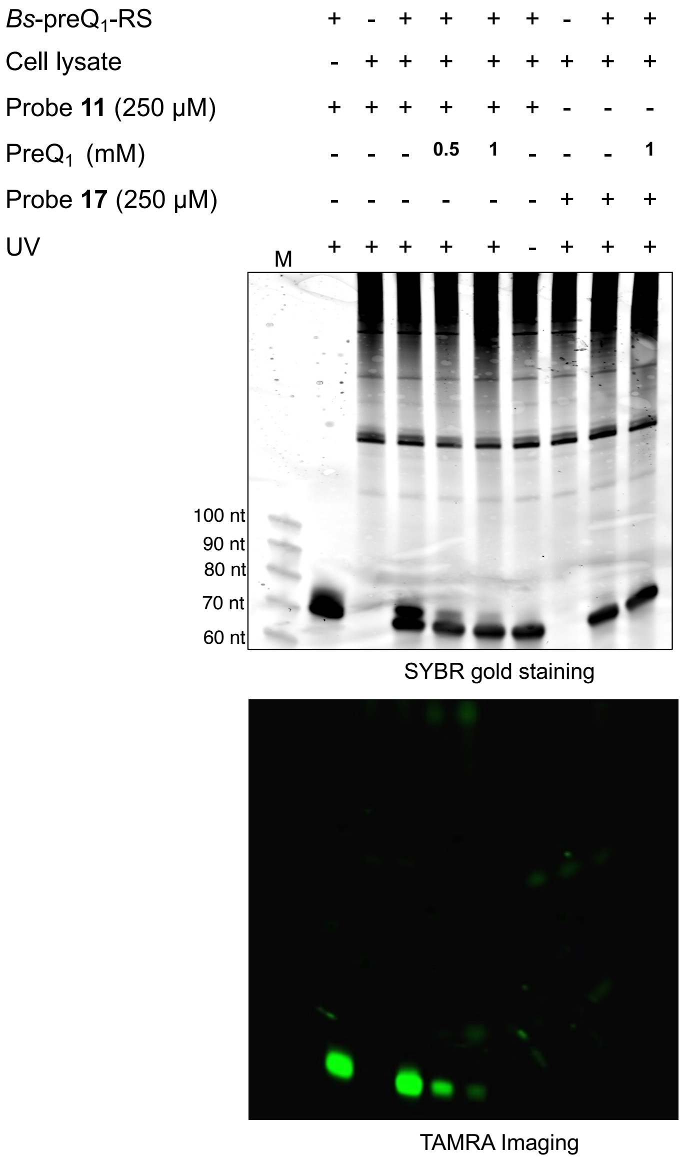

Figure S11: Competitive photocrosslinking experiments in MCF-7 total RNA imaged by SYBR gold stain (top) and TAMRA labeling (bottom). Compound **11** (250 μM) selectively labeled the *Bs*-preQ$_1$-RS full length (1 μM, or 1.1 μg) in the presence of cellular total RNA (5 μg) while no detectable crosslinked product was observed with compound **17** (250 μM). Co-incubation of **11** (250 μM) with excess PreQ$_1$ (500 μM and 1000 μM concentrations) resulted in competitive decrease of TAMRA signal. The experiment was repeated three time independently.

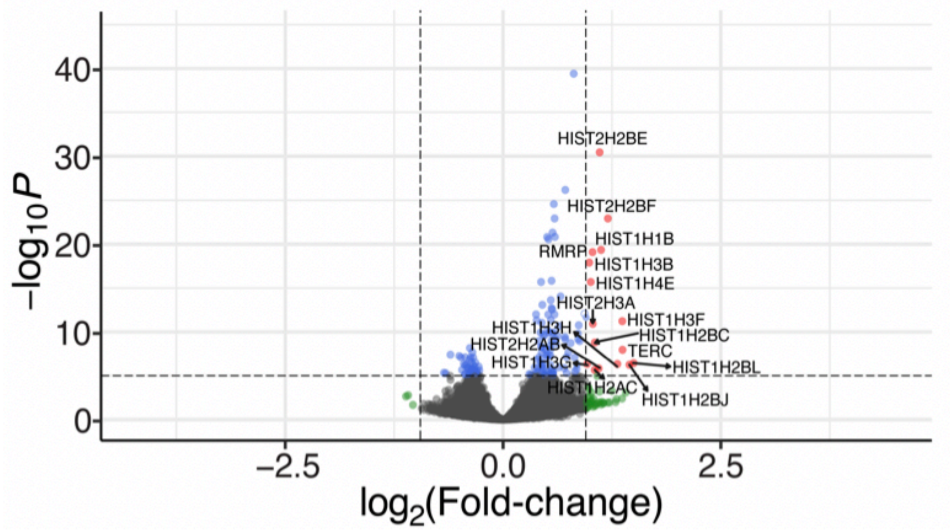

Gene Symbol	log_2(Fold-change)	Adj. P-value
HIST2H2BE	1.11	3.5e-31
HIST2H2BF	1.20	1.2e-23
HIST1H1B	1.12	4.6e-20
HIST1H3B	1.13	4.6e-20
RMRP	0.98	1.3e-18
HIST1H4E	1.01	2.0e-16
HIST2H3A	1.03	1.2e-11
HIST1H3F	1.37	5.9e-12
HIST1H2BC	1.05	1.6e-9
TERC	1.37	1.1e-8
HIST1H3H	1.31	4.7e-7
HIST1H3G	1.10	1.5e-6
HIST1H2BL	1.50	3.8e-7
HIST1H2BJ	1.45	5.8e-7
HIST1H2AC	1.05	2.3e-6
HIST2H2AB	1.40	8.4e-4

Figure S12: Volcano Plot showing differential expression analysis (DeSeq2) between **11** and **17** treated samples. The points in red are significantly enriched (log_2(Fold-change) > 0.95, adjusted p-value < 10^{-3}). These hits are labelled with the gene name on the plot. Corresponding values for enrichment and significance are provided in the accompanying table.

Section 2:

Synthesis and characterization of electrophoretic and photoaffinity probes

A) Substitution reaction between PreQ1 and alkyl halides

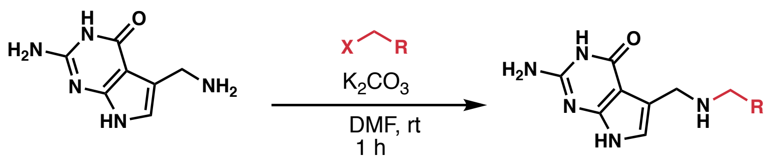

To a solution of PreQ1•2TFA (20 mg, 49 μmol, 1.0 equiv.) in DMF, K_2CO_3 (21 mg, 15 μmol, 3.1 equiv.) and alkyl halide (49 μmol, 1.0 equiv.) were added, and the reaction mixture was stirred at room temperature for 1 h. Then, the reaction mixture was concentrated, and purified via column chromatography on a silica gel using gradient based MeOH:DCM (0-20 % MeOH) on CombiFlash® Rf system.

B) Coupling reaction between PreQ1 and carboxylic acid-based substrates

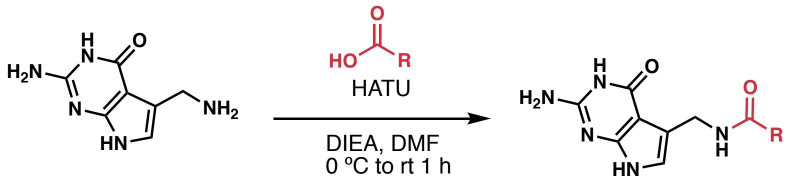

To a solution of a carboxylic acid substrate (37 μmol, 1.0 equiv.) in DMF (1 mL) cooled to 0 °C, HATU (14 mg, 37 μmol, 1.0 equiv.) and diisopropylethylamine (20 μL, 118 μmol, 3.2 equiv.) were added and stirred for 10 min. Then, PreQ1•2TFA (15 mg, 37 μmol, 1.0 equiv.) was added, warmed the reaction mixture to room temperature and stirred for 1 hr. Then, the reaction mixture was concentrated and purified via column chromatography on a silica gel eluting with gradient based MeOH:DCM on CombiFlash® Rf system or preparative HPLC eluting with 10-90 % gradient of H_2O:MeCN containing 0.1% TFA.

Mass Spectral Analysis

LC/MS analysis of accurate mass measurements was carried out on the Orbitrap LTQ-XL system. A 2.0-μL aliquot of an analytical solution (about 58 μg/ml in LC/MS-grade 1:1 CH_3CN/H_2O) was injected onto a 2.1 X 100 mm, 3.5-μm Zorbax SB-C18 narrow-bore HPLC column and eluted at flow rate of 250 μL/min with a 15-min combination of both isocratic and linear gradient elution using mobile phase varying from 2-90% CH_3CN/H_2O and containing 0.1% formic acid.

Compound 1 (Methyl (*E*)-4-(((2-amino-4-oxo-4,7-dihydro-3*H*-pyrrolo[2,3-*d*]pyrimidin-5-yl)methyl)amino)but-2-enoate).

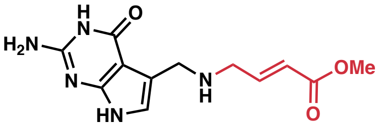

Prepared according to the general procedure **A** isolated as TFA salt (5.0 mg, 35% yield) . White solid. ^{1}H NMR (500 MHz, DMSO): δ 11.32 (s, 1H), 10.97 (s, 1H), 9.37 (brs, 2H), 6.92-6.83 (m, 1H), 6.84 (s, 1H), 6.42 (brs, 2H), 6.24 (d, *J* = 15.4 Hz, 1H), 4.20 (t, *J* = 5.2 Hz, 2H), 3.86 (m, 2H), 3.71 (s, 1H); ^{13}C{^{1}H} NMR (126 MHz, DMSO): 165.69, 160.81, 158.54 (q, *J* = 35.4 Hz), 153.18, 152.58, 139.05, 125.68, 118.12, 115.4 (q, *J* = 294.0 Hz), 108.54, 98.71, 52.23, 46.28, 43.18; HRMS m/z: calcd. for $C_{12}H_{16}N_5O_3$ [M+H]$^+$ 278.1248, found 278.1240.

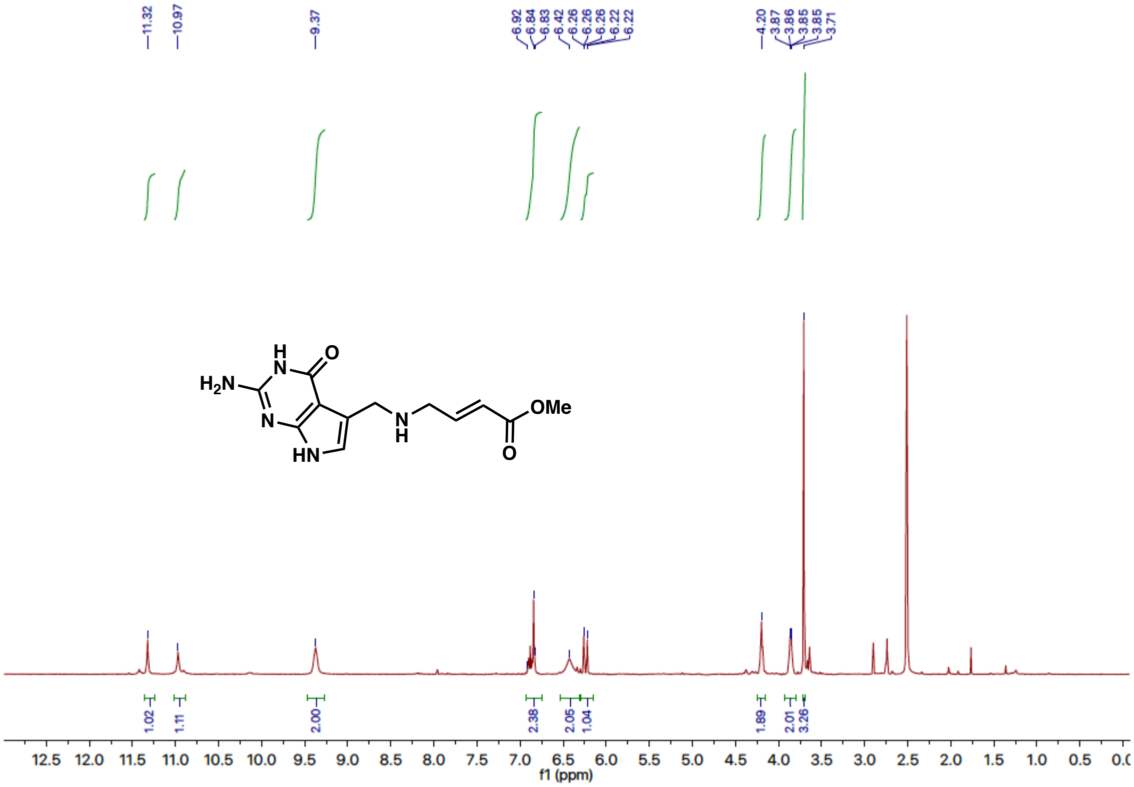

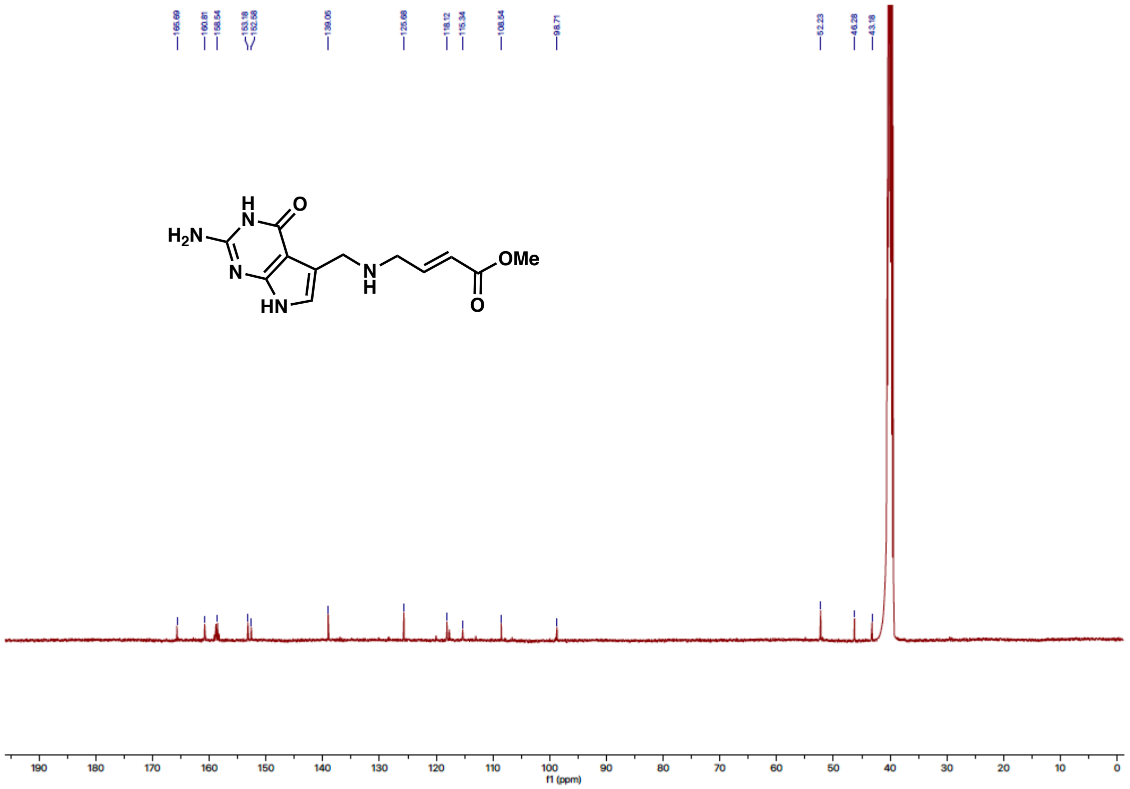

Compound 2 (*N*-((2-amino-4-oxo-4,7-dihydro-3*H*-pyrrolo[2,3-*d*]pyrimidin-5-yl)methyl)methacrylamide).

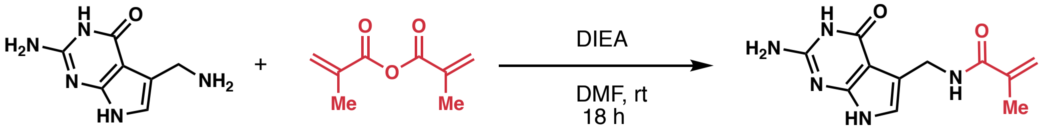

To a solution of PreQ$_1$•2HCl (20 mg, 80 μmol, 1.0 equiv.) and DIEA (30 μL, 160 μmol, 2.0 equiv.) in DMF (1.0 mL), methacrylic anhydride (15 μL, 96 μmol, 1.2 equiv.) was added and the reaction mixture was stirred at room temperature for 18 h. Then, the reaction mixture was concentrated and via column chromatography on a silica gel using MeOH:DCM. White solid. ^{1}H NMR (500 MHz, DMSO): δ 10.89 (s, 1H), 10.49 (s, 1H), 8.74 (t, J = 5.4 Hz, 1H) , 6.54 (s, 1H), 6.14 (s, 2H), 5.70 (s, 1H), 5.33 (t, J = 1.6 Hz, 1H), 4.35 (d, J = 5.4 Hz, 2H),1.89 (s, 3H); ^{13}C{^{1}H} NMR (126 MHz, DMSO): 166.9, 160.4, 152.7, 152.3, 140.5, 119.5, 115.6, 114.2, 99.0, 35.8, 19.0; HRMS m/z: calcd. for $C_{11}H_{14}N_5O_2$ $[M+H]^+$ 248.1142, found 248.1137.

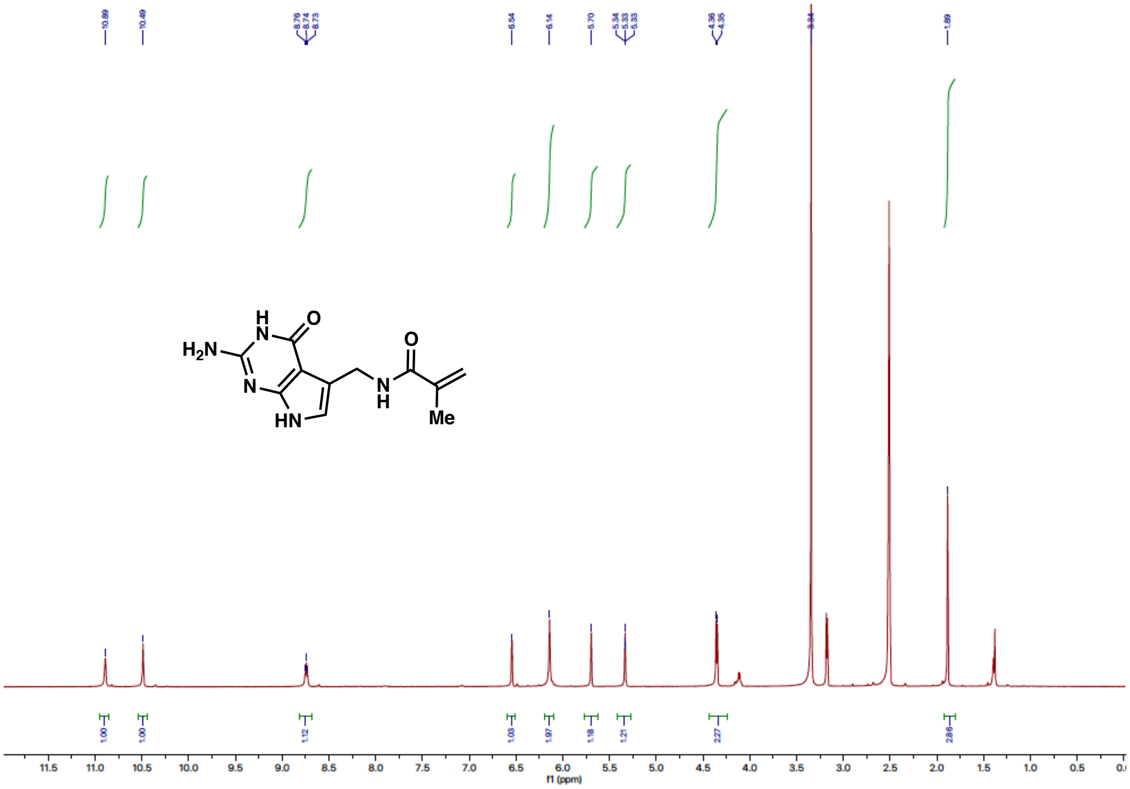

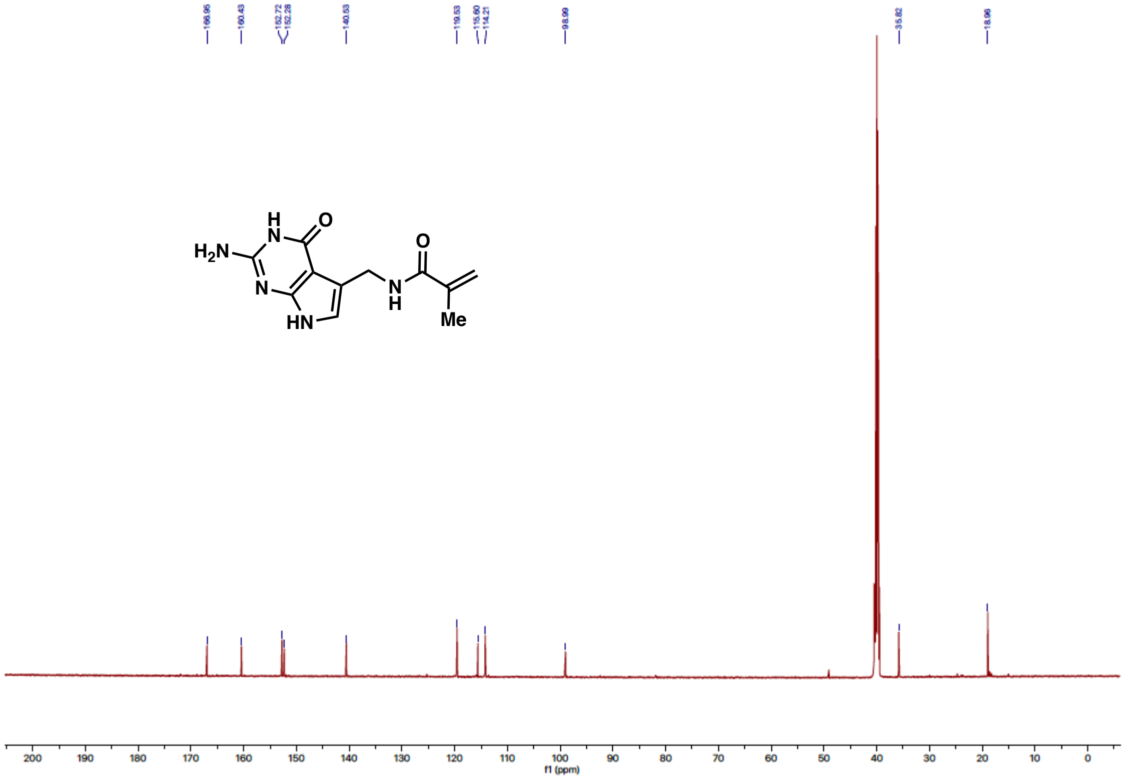

Compound 3 ((*E*)-*N*-((2-amino-4-oxo-4,7-dihydro-3*H*-pyrrolo[2,3-*d*]pyrimidin-5-yl)methyl)-2-phenylethene-1-sulfonamide).

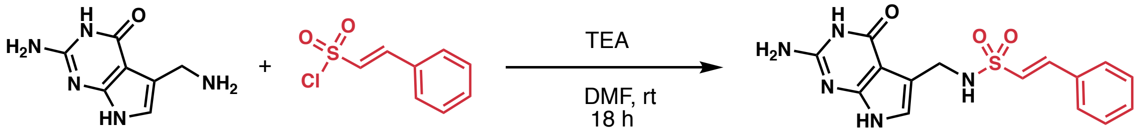

The reaction is run according the procedure for compound **6**. White solid. ^{1}H NMR (500 MHz, DMSO): δ 10.88 (s, 1H), 10.67 (brs, 1H), 7.73 (brs, 1H), 7.56-7.54 (m, 2H), 7.41-7.38 (m, 3H), 7.30 (d, *J* = 15.6 Hz, 1H) , 7.03 (d, *J* = 15.6 Hz, 1H) , 6.59 (s, 1H), 6.21 (s, 2H), 4.15 (d, *J* = 3.8 Hz, 2H); $^{13}C\{^{1}H\}$ NMR (126 MHz, DMSO): 160.4, 152.9, 152.3, 139.4, 133.4, 130.8, 129.3, 128.6, 127.5, 114.8, 98.8; HRMS m/z: calcd. for $C_{15}H_{16}N_{5}O_{3}S$ $[M+H]^{+}$ 346.0968, found 346.0955.

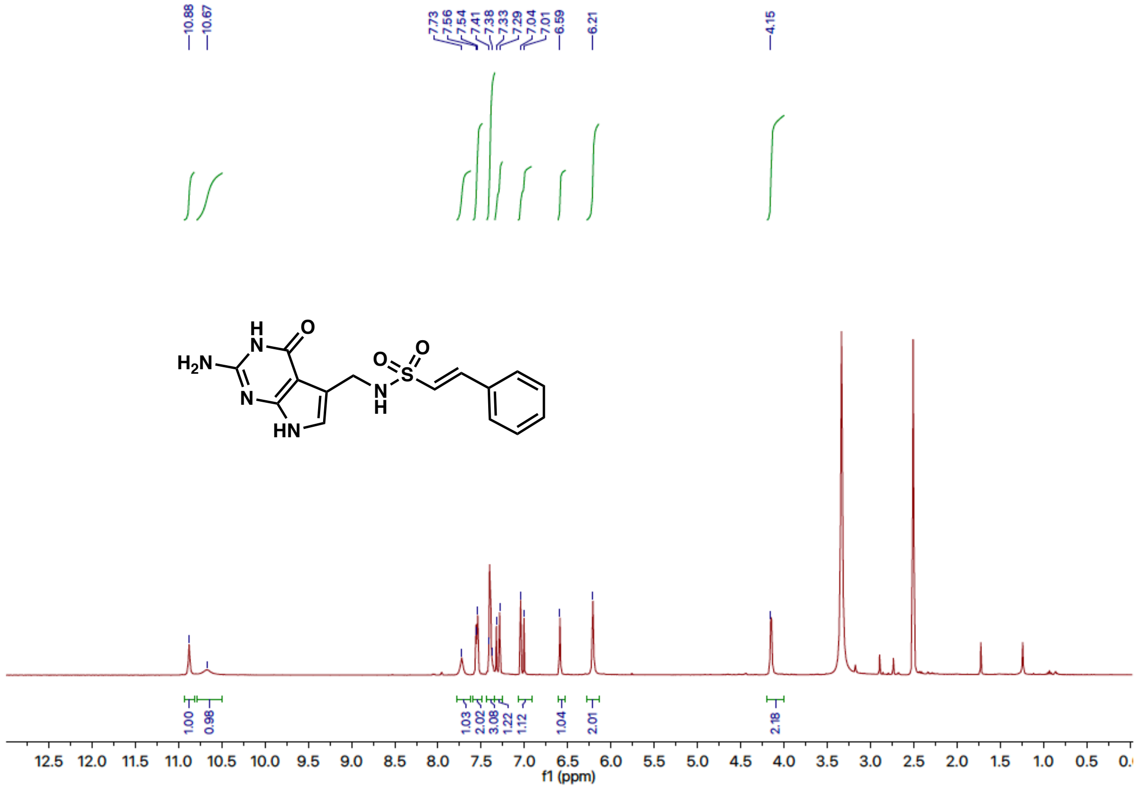

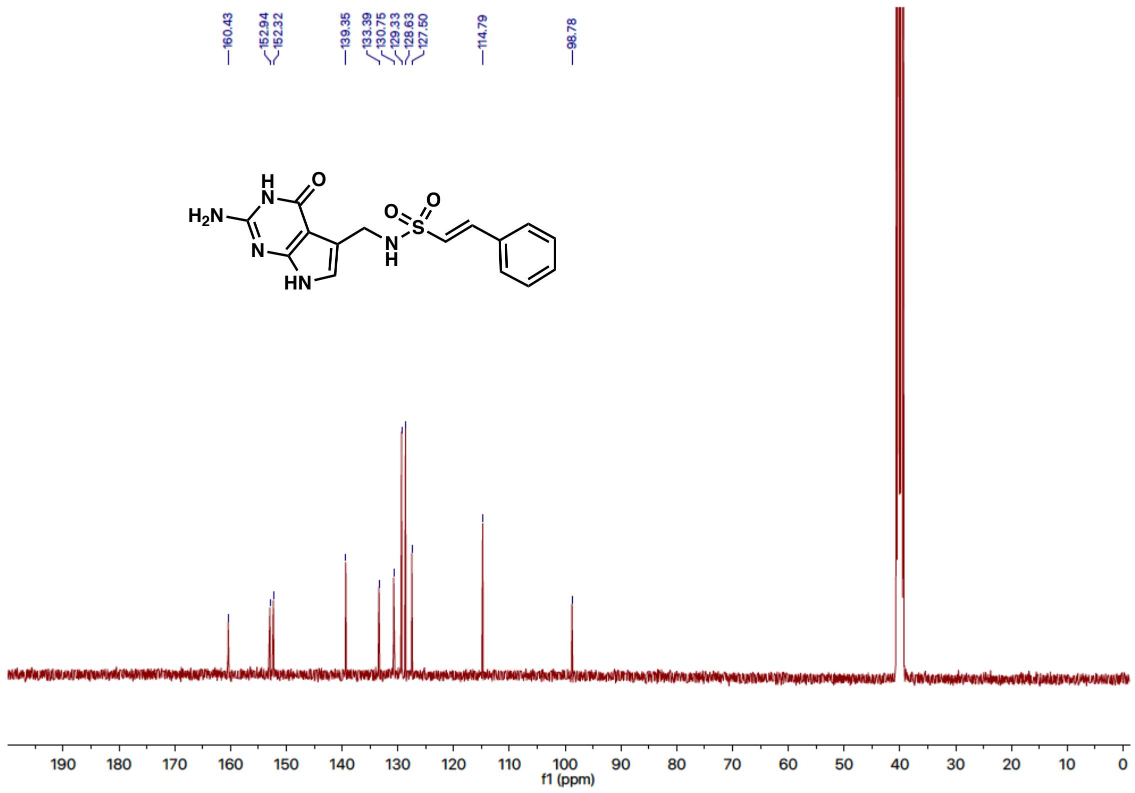

Compound 4 (*N*-((2-amino-4-oxo-4,7-dihydro-3*H*-pyrrolo[2,3-*d*]pyrimidin-5-yl)methyl)-2-(2,5-dioxo-2,5-dihydro-1*H*-pyrrol-1-yl)acetamide).

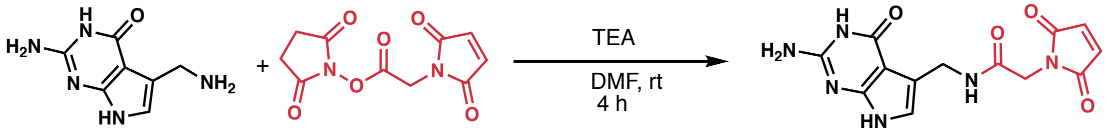

To a solution of PreQ$_1$•2TFA (20 mg, 49 μmol, 1.0 equiv.) and triethylamine (15 μL, in DMF (1.0 mL) at room temperature, maleimidoacetic acid *N*-hydroxysuccinimide ester (12 mg, 49 μmol, 1.0 equiv.) was added and stirred for 4 h. Then, the reaction mixture was concentrated, and purified via column chromatography using a silica gel and eluting with gradient based MeOH:DCM to obtain the titled compound as a white solid. ^{1}H NMR (500 MHz, DMSO): δ 10.86 (s, 1H), 10.34 (s, 1H), 8.57 (t, J = 5.4 Hz, 1H), 7.11 (s, 2H), 6.49 (s, 1H), 6.08 (s, 2H), 4.30 (d, J = 5.4 Hz, 2H) , 4.04 (s, 2H); ^{13}C{^{1}H} NMR (126 MHz, DMSO): 171.18, 165.99, 159.95, 152.73, 151.96, 135.33, 115.36, 114.34, 98.78, 35.67; HRMS m/z: calcd. for $C_{13}H_{13}N_6O_4$ [M+H]$^+$ 317.0993, found 317.0982.

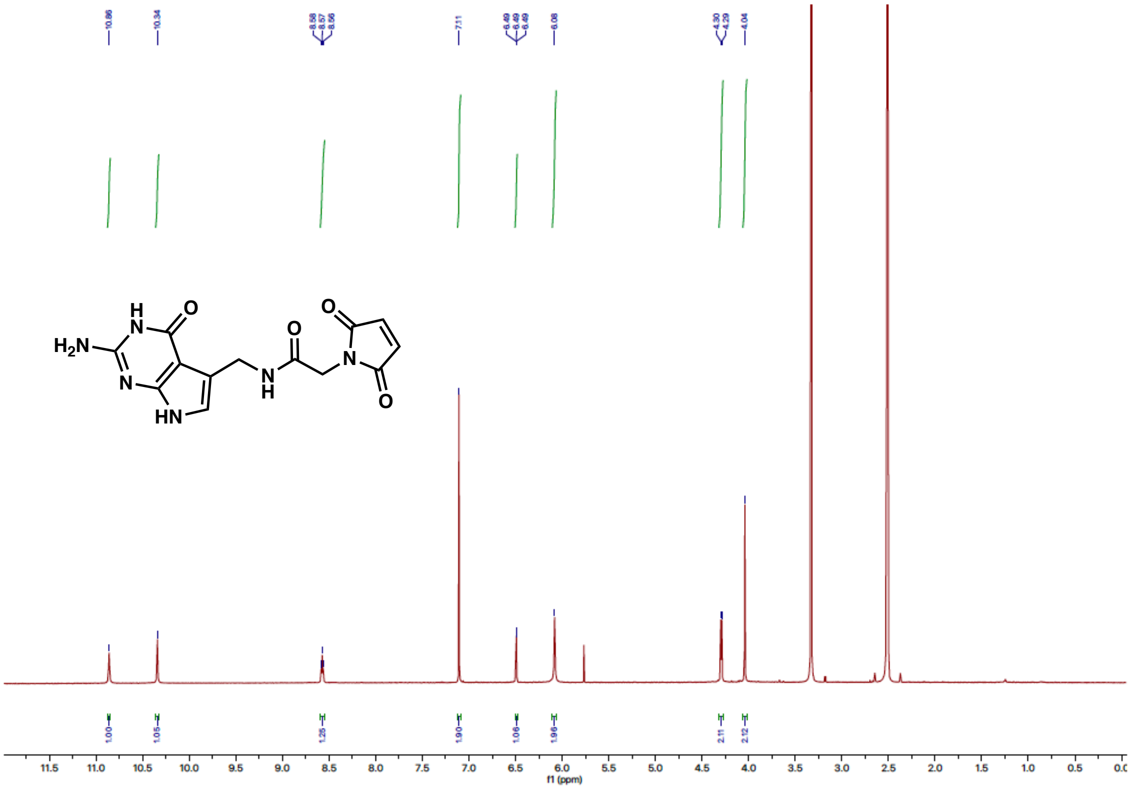

f1 (ppm)

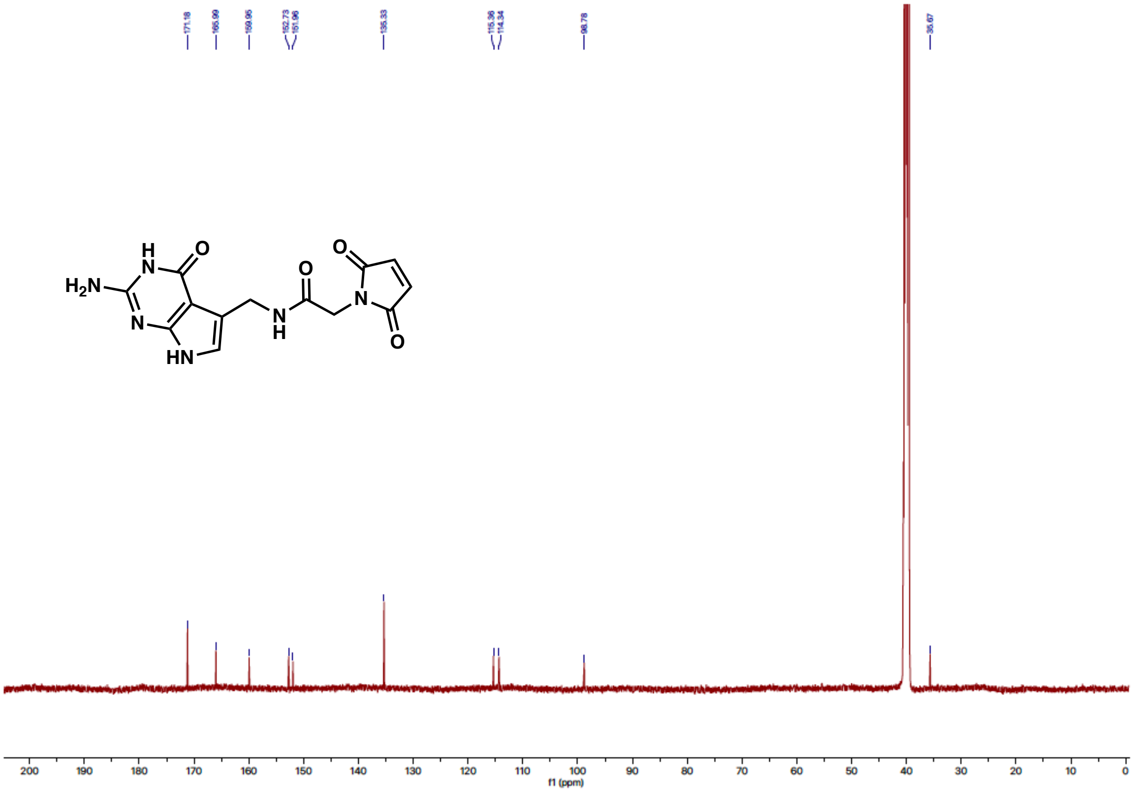

f1 (ppm)

Compound 5 (*N*-((2-amino-4-oxo-4,7-dihydro-3*H*-pyrrolo[2,3-*d*]pyrimidin-5-yl)methyl)-3-(2,5-dioxo-2,5-dihydro-1*H*-pyrrol-1-yl)propenamide).

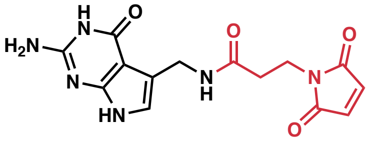

Prepared according to the procedure described for Compound **4**. White solid. ^{1}H NMR (500 MHz, DMSO): δ 10.82 (s, 1H), 10.31 (s, 1H), 8.29 (t, *J* = 5.4 Hz, 1H), 6.97 (s, 2H), 6.46 (brs, 1H), 6.07 (s, 2H), 4.24 (d, *J* = 5.1 Hz, 2H), 3.62 (m, 2H), 2.37 (m, 2H); ^{13}C{^{1}H} NMR (126 MHz, DMSO): 171.2, 169.2, 160.0, 152.7, 151.9, 135.0, 115.7, 114.3, 98.8, 46.2, 35.5, 34.5; HRMS m/z: calcd. for $C_{14}H_{15}N_6O_4$ $[M+H]^+$ 331.1149, found 331.1138.

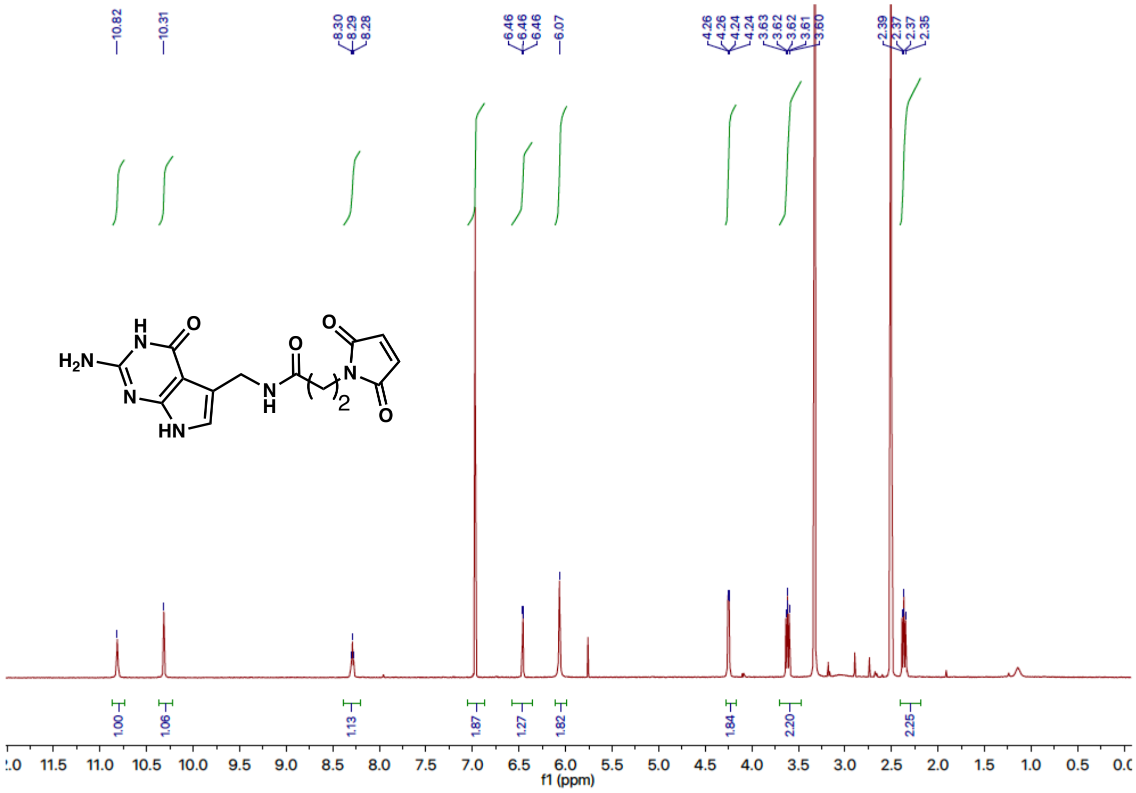

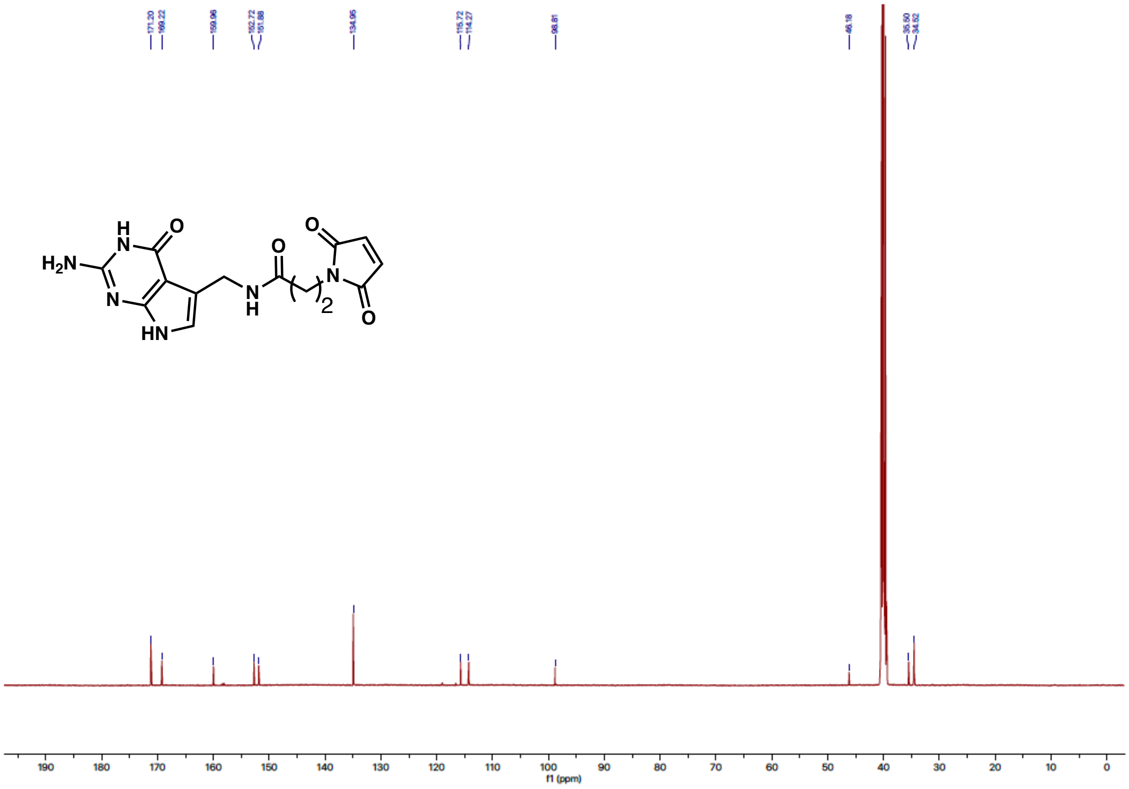

Compound 6 (Ethyl ((2-amino-4-oxo-4,7-dihydro-3*H*-pyrrolo[2,3-*d*]pyrimidin-5-yl)methyl)carbamate).

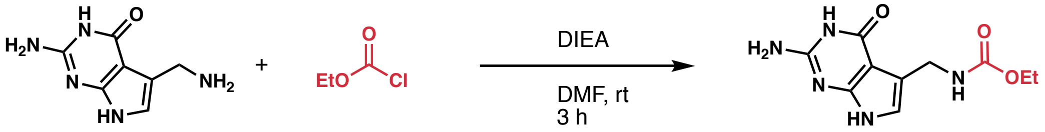

A solution of PreQ$_1$•2HCl (20 mg, 80 μmol, 1.0 equiv.) in DMF (1.0 mL) and cooled to 0 ºC was treated with diisopropylethylamine (27 μL, 160 μmol, 2.0 equiv.). Then, ethyl chloroformate (8 μL, 88 μmol, 1.1 equiv.) was added, the reaction mixture was allowed to warm to room temperature and stirred for 3 h. Consequently, the reaction mixture was concentrated, triturated with 20% MeOH/DCM, and the resulting precipitate was collected to obtain the titled compound as a white solid. ^{1}H NMR (500 MHz, DMSO): δ 11.31 (s, 1H), 11.23 (s, 1H), 7.31 (s, 1H), 6.58 (s, 1H), 4.23 (s, 2H), 3.99 (q, J = 7.1 Hz, 2H),1.16 (t, J = 7.1 Hz, 3H); ^{13}C{^{1}H} NMR (126 MHz, DMSO): 158.2, 156.1, 151.5, 116.8, 114.5, 98.4, 59.7, 36.5, 14.7; HRMS m/z: calcd. for $C_{10}H_{14}N_5O_3$ [M+H]$^+$ 252.1091, found 252.1084.

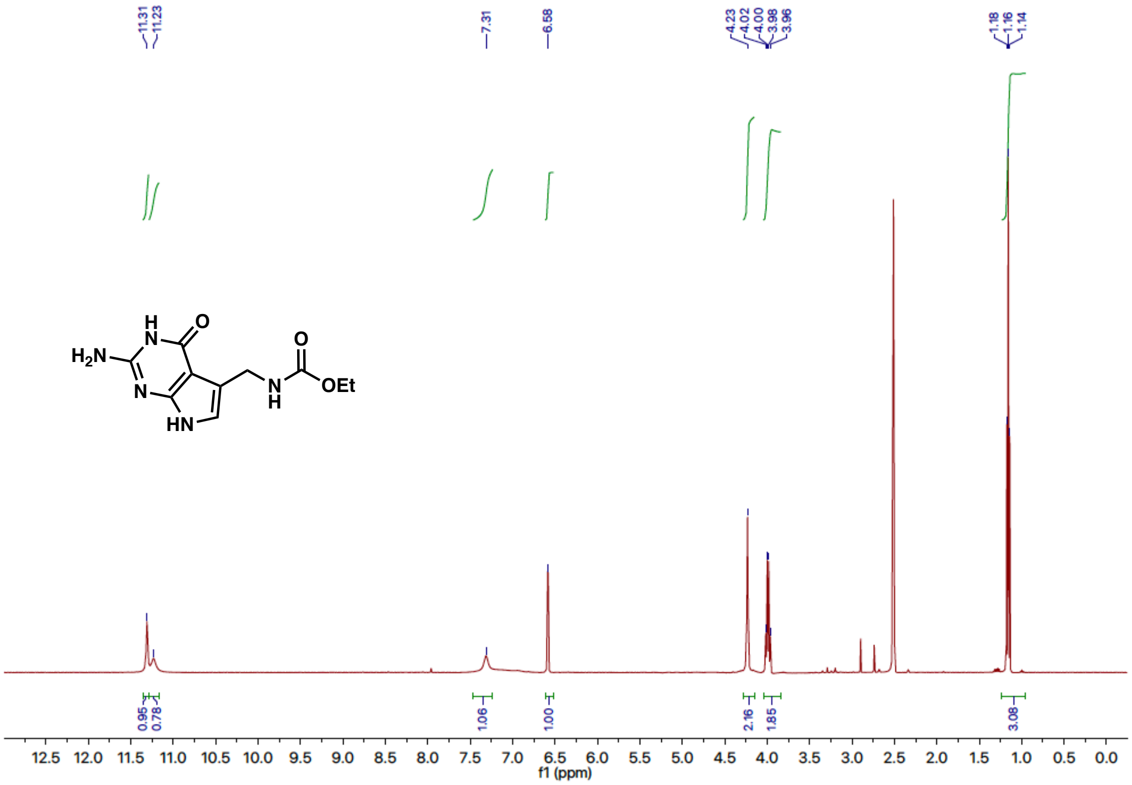
11.31
11.23
7.31
6.58
4.23
4.02
4.00
3.98
3.96
1.18
1.16
1.14
H
N
O
H2N
N
HN
N
H
O
OEt
0.95
0.78
1.06
1.00
2.16
1.85
3.08
12.5 12.0 11.5 11.0 10.5 10.0 9.5 9.0 8.5 8.0 7.5 7.0 6.5 6.0 5.5 5.0 4.5 4.0 3.5 3.0 2.5 2.0 1.5 1.0 0.5 0.0
f1 (ppm)

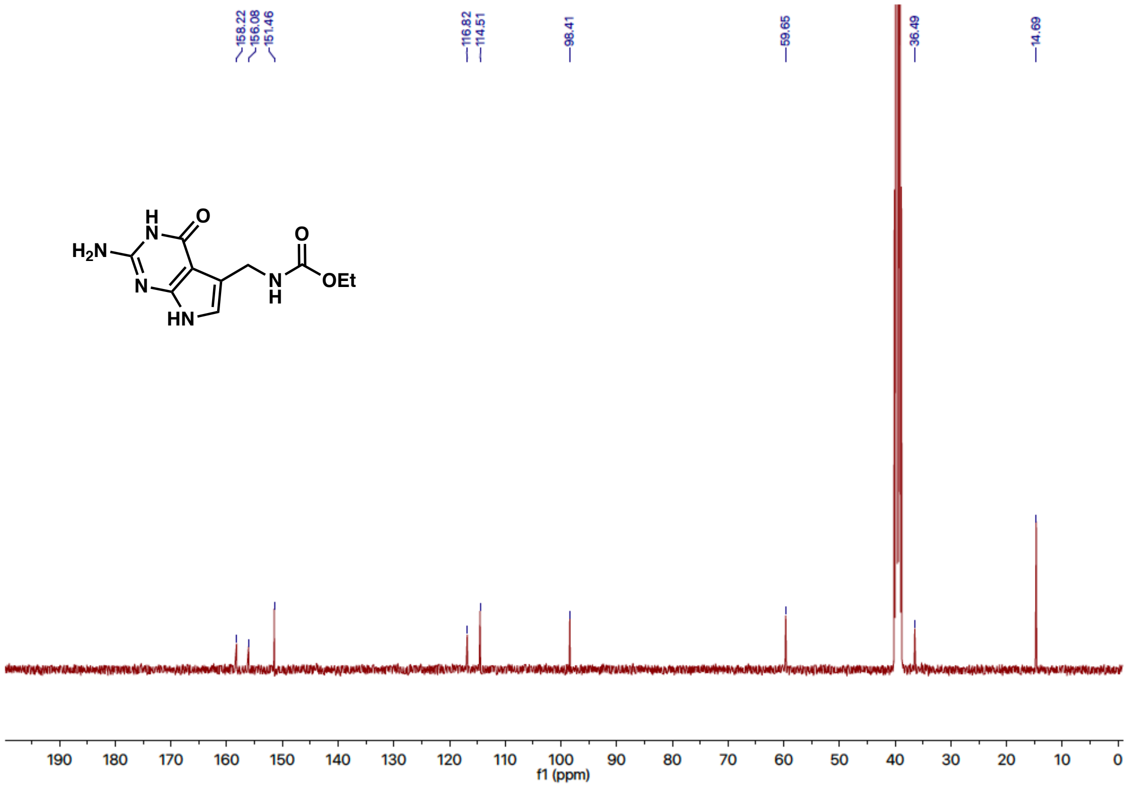
158.22
156.08
151.46
116.82
114.51
98.41
59.65
36.49
14.69
H
N
O
H2N
N
HN
N
H
O
OEt
190 180 170 160 150 140 130 120 110 100 90 80 70 60 50 40 30 20 10 0
f1 (ppm)

Compound 7 (4-nitrophenyl ((2-amino-4-oxo-4,7-dihydro-3*H*-pyrrolo[2,3-*d*]pyrimidin-5-yl)methyl)carbamate).

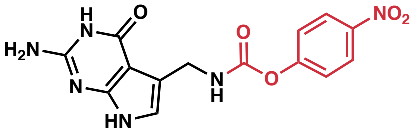

Prepared according the procedure for compound **9**. White solid. ^{1}H NMR (500 MHz, DMSO): δ 10.92 (s,1H), 10.43 (s, 1H), 8.37 (s, 1H), 8.27 (d, *J* = 8.2 Hz, 2H) , 7.43 (d, *J* = 8.2 Hz, 2H), 6.61 (s, 1H), 6.13 (s, 2H), 4.36 (brs, 2H); ^{13}C{^{1}H} NMR (125 MHz, DMSO): 160.10, 153.36, 152.81, 152.14, 144.58, 125.58, 125.58, 123.00, 115.12, 114.50, 98.80, 37.76; HRMS m/z: calcd. for $C_{14}H_{13}N_6O_5$ $[M+H]^+$ 345.0942, found 345.0933.

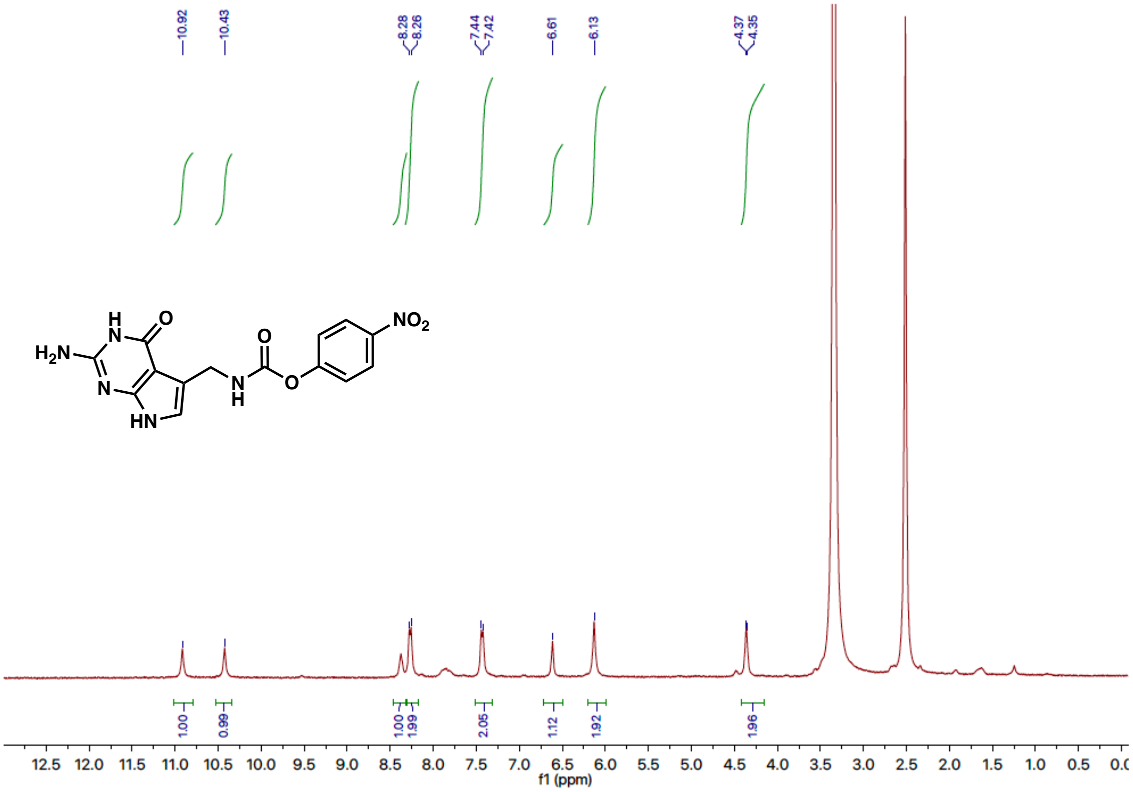

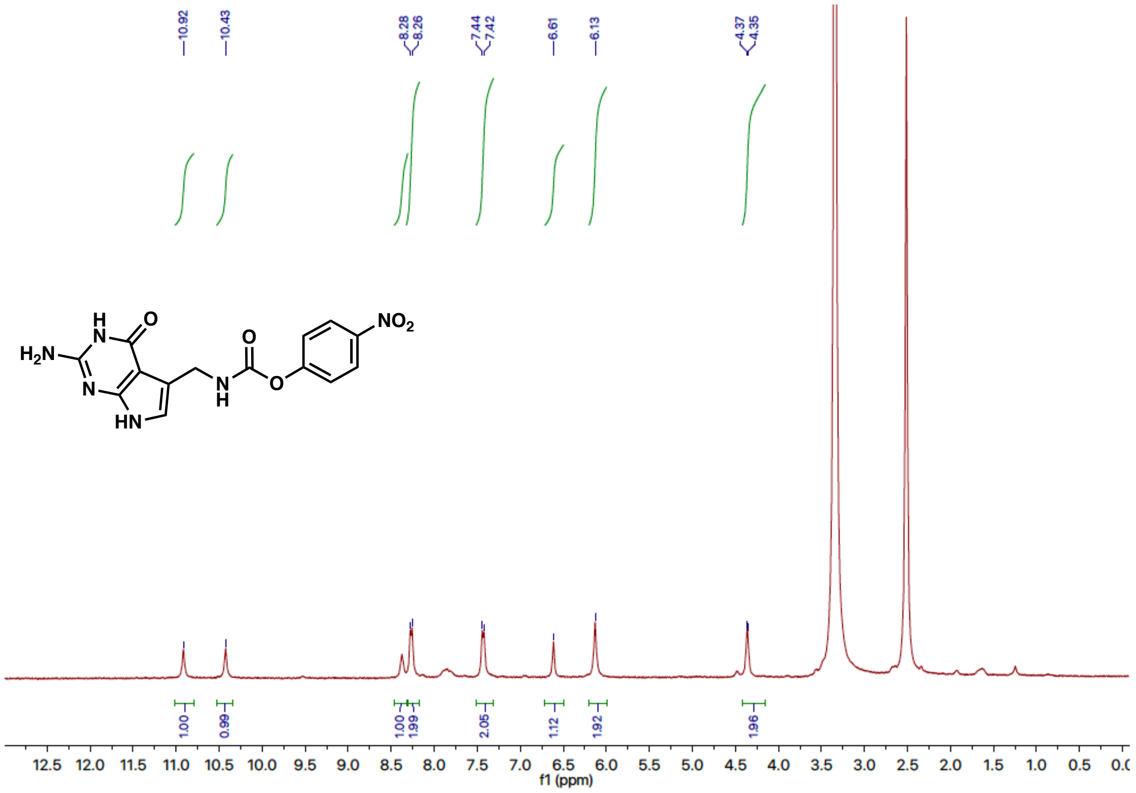

Compound 8 (*N*-((2-amino-4-oxo-4,7-dihydro-3*H*-pyrrolo[2,3-*d*]pyrimidin-5-yl)methyl)-1*H*-imidazole-1-carboxamide).

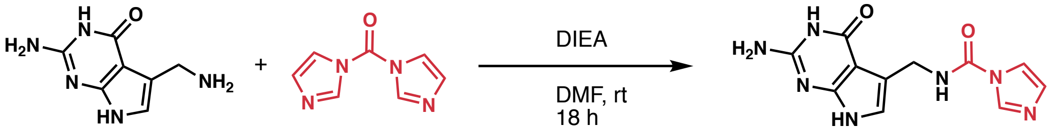

To a solution of $PreQ_1$•2HCl (20 mg, 80 μmol, 1.0 equiv.) in DMF (1.0 mL), carbonyldiimidazole was added and the resulting reaction mixture was stirred at room temperature for 18 h. Then, the reaction mixture was concentrated, triturated with 20% MeOH/DCM, and the resulting precipitate was collected to obtain the titled compound as a white solid. ^{1}H NMR (500 MHz, DMSO): δ 11.03 (s, 1H), 10.58 (s, 1H) 9.39 (t, J = 5.3 Hz, 1H), 8.30 (s, 1H), 7.73 (s, 1H), 7.09 (s, 1H), 6.70 (s, 1H), 6.21 (s, 2H), 4.56 (d, J = 5.3 Hz, 2H); $^{13}C\{^{1}H\}$ NMR (126 MHz, DMSO): 159.88, 152.27, 151.77, 148.42, 135.87, 129.57, 116.44, 114.43, 114.12, 98.32, 36.75; HRMS m/z: calcd. for $C_{11}H_{12}N_7O_2$ $[M+H]^+$ 274.1047, found 274.1040.

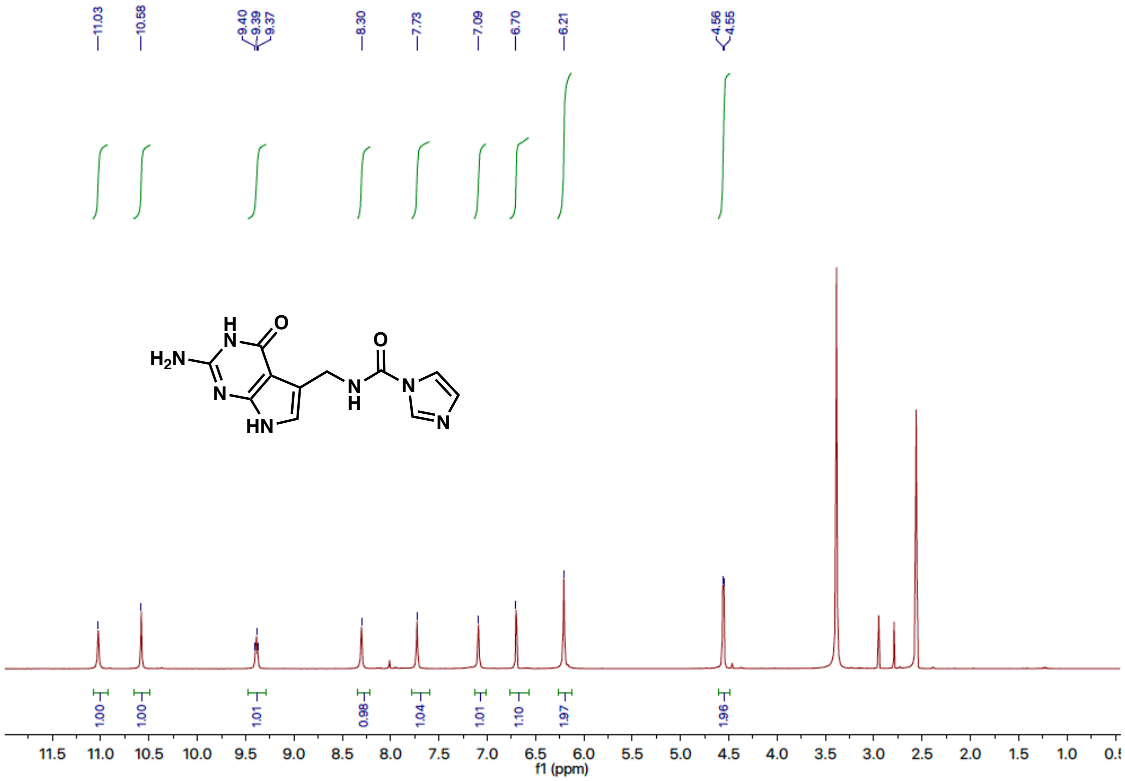
11.03
10.58
9.40
9.39
9.37
8.30
7.73
7.09
6.70
6.21
4.56
4.55
H2N
H
N
O
N
HN
N
H
O
N
N
1.00
1.00
1.01
0.98
1.04
1.01
1.10
1.97
1.96
11.5 11.0 10.5 10.0 9.5 9.0 8.5 8.0 7.5 7.0 6.5 6.0 5.5 5.0 4.5 4.0 3.5 3.0 2.5 2.0 1.5 1.0
f1 (ppm)

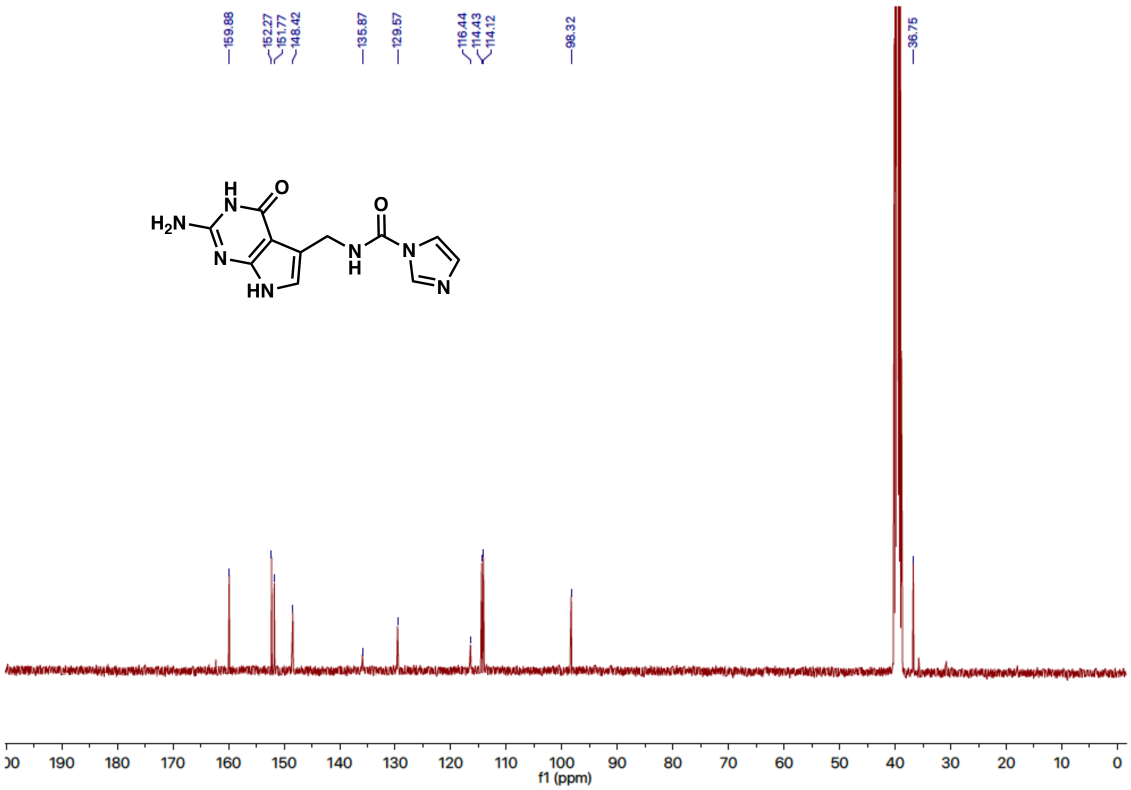
159.88
152.27
151.77
148.42
135.87
129.57
116.44
114.43
114.12
98.32
36.75
H2N
H
N
O
N
HN
N
H
O
N
N
190 180 170 160 150 140 130 120 110 100 90 80 70 60 50 40 30 20 10 0
f1 (ppm)

Compound 9 (3-(((2-amino-4-oxo-4,7-dihydro-3*H*-pyrrolo[2,3-*d*]pyrimidin-5-yl)methyl)amino)-4-methoxycyclobut-3-ene-1,2-dione).

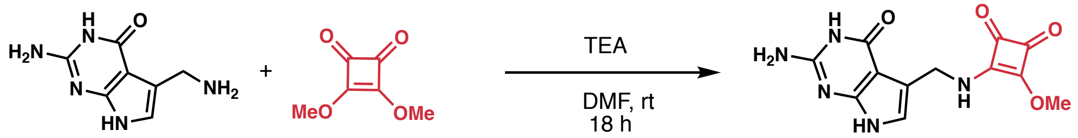

To a solution of PreQ$_1$•2TFA in anhydrous DMF (600 μL) under N_2, dimethyl squarate (7.0 mg, 49 μmol, 1.0 equiv.) and triethylamine (15 μL, 103 μmol, 2.1 equiv.) dissolved in anhydrous DMF (400 μL) was added and stirred at room temperature for 18 h. Then, the reaction mixture was concentrated under reduced pressure, diluted with 1.0 mL of MeOH, and resulting precipitate was collected by filtration to obtain the titled compound as a white solid (57% yield). ^{1}H NMR (500 MHz, DMSO): δ 10.94 (s, 1H), 10.37 (s, 1H), 9.02-8.92 (s, 1H), 6.58 (d, J = 2.1 Hz, 1H), 6.13 (s, 2H), 4.75 (d, J = 5.6 Hz, 1H), 4.54 (d, J = 5.6 Hz, 1H), 4.30-4.27 (s, 3H); ^{13}C{^{1}H} NMR (126 MHz, DMSO): 190.0, 189.7, 172.5, 172.2, 159.9, 159.9, 157.5, 152.9, 152.5, 152.2, 114.9, 114.4, 98.6, 98.6, 60.6, 60.3, 41.1 (Note that the compound exist as a mixture of tautomers and as a result, there are more carbon peaks observed); HRMS m/z: calcd. for $C_{12}H_{12}N_5O_4$ [M+H]$^+$ 290.0884, found 290.0877.

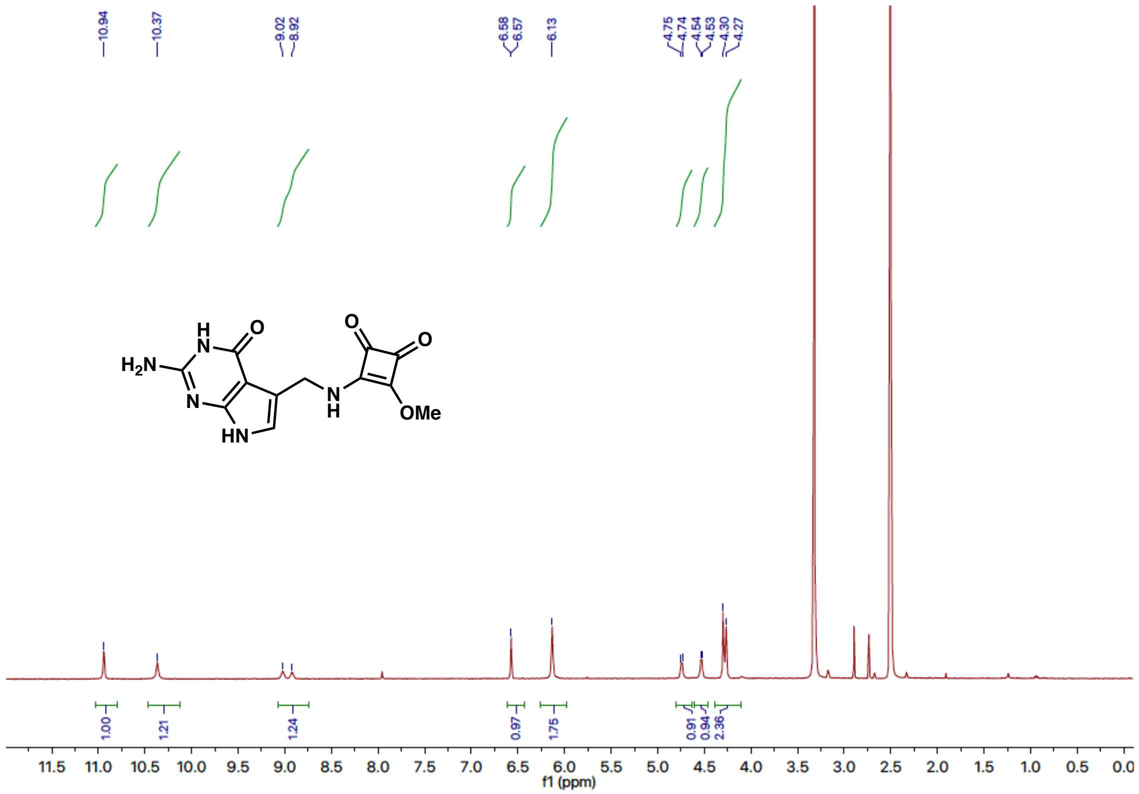

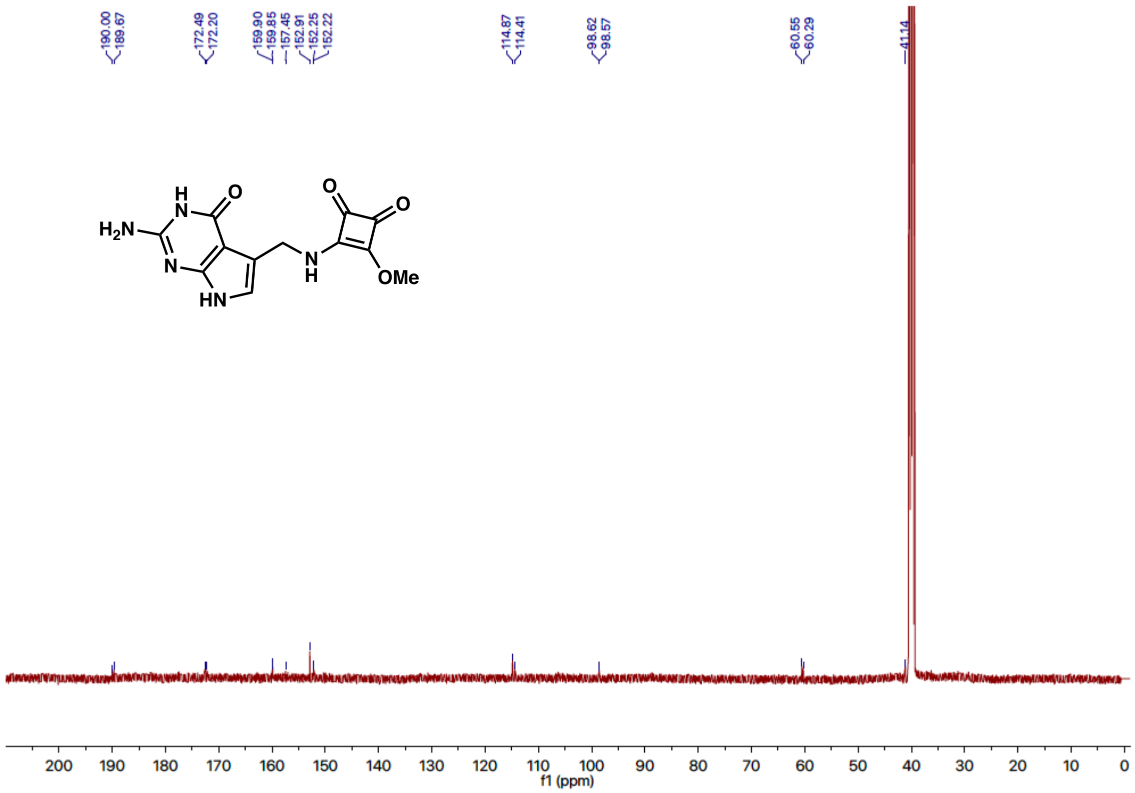

Compound 10 (*N*-((2-amino-4-oxo-4,7-dihydro-3*H*-pyrrolo[2,3-*d*]pyrimidin-5-yl)methyl)-4-(4-(bis(2-chloroethyl)amino)phenyl)butanamide).

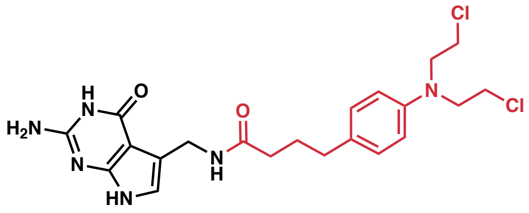

Prepared according to the general procedure **B**. ^{1}H NMR (500 MHz, DMSO): δ 10.89 (s, 1H), 10.44 (s, 1H), 8.24 (t, *J* = 5.4 Hz, 1H), 7.06 (d, *J* = 8.7 Hz, 2H) , 6.71 (d, *J* = 8.7 Hz, 2H) , 6.54 (s, 1H), 6.18 (s, 2H), 4.35 (d, *J* = 5.3 Hz, 2H), 3.79-3.73 (m, 8H), 2.47 (t, *J* = 7.5 Hz, 2H), 2.16 (t, *J* = 7.5 Hz, 2H),1.79 (qt, *J* = 7.5 Hz, 2H); $^{13}C\{^{1}H\}$ NMR (126 MHz, DMSO): 71.7, 160.0, 152.7, 152.0, 144.8, 130.4, 129.8, 116.0, 114.1, 112.3, 98.8, 52.7, 41.6, 35.6, 35.5, 34.1, 27.9; HRMS m/z: calcd. for $C_{21}H_{27}Cl_2N_6O_2$ $[M+H]^+$ 465.1567, found 465.1555.

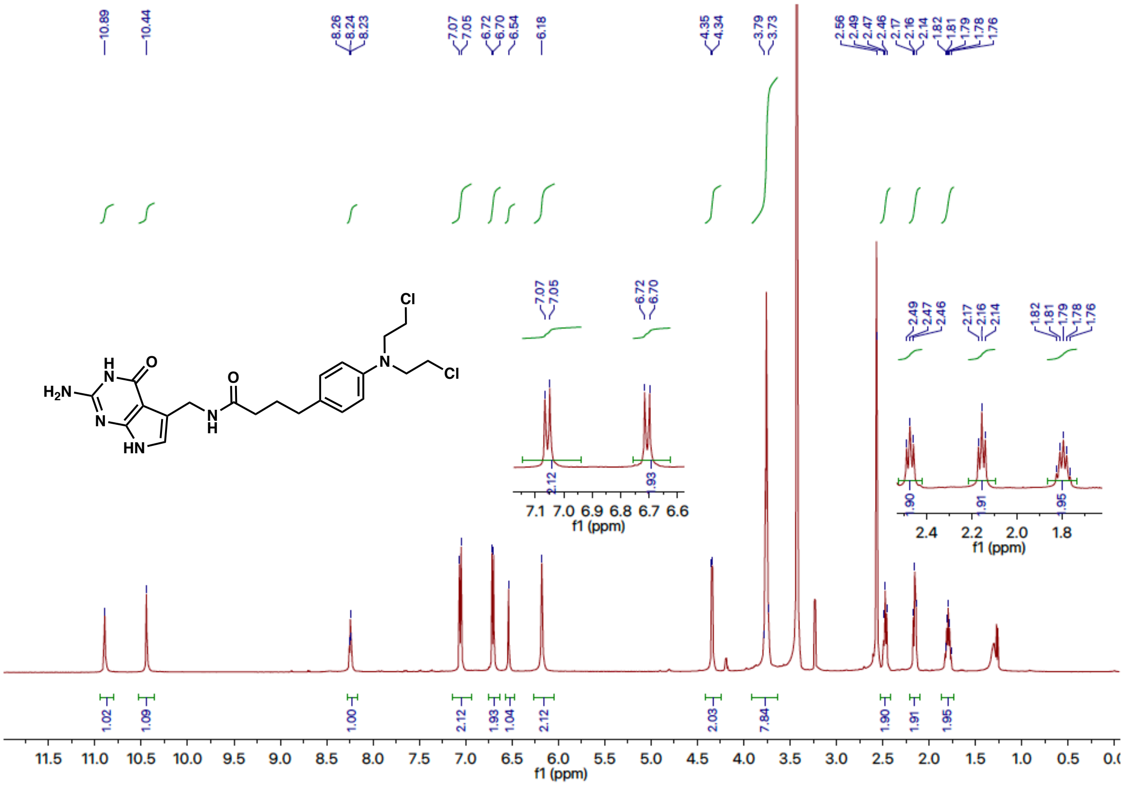

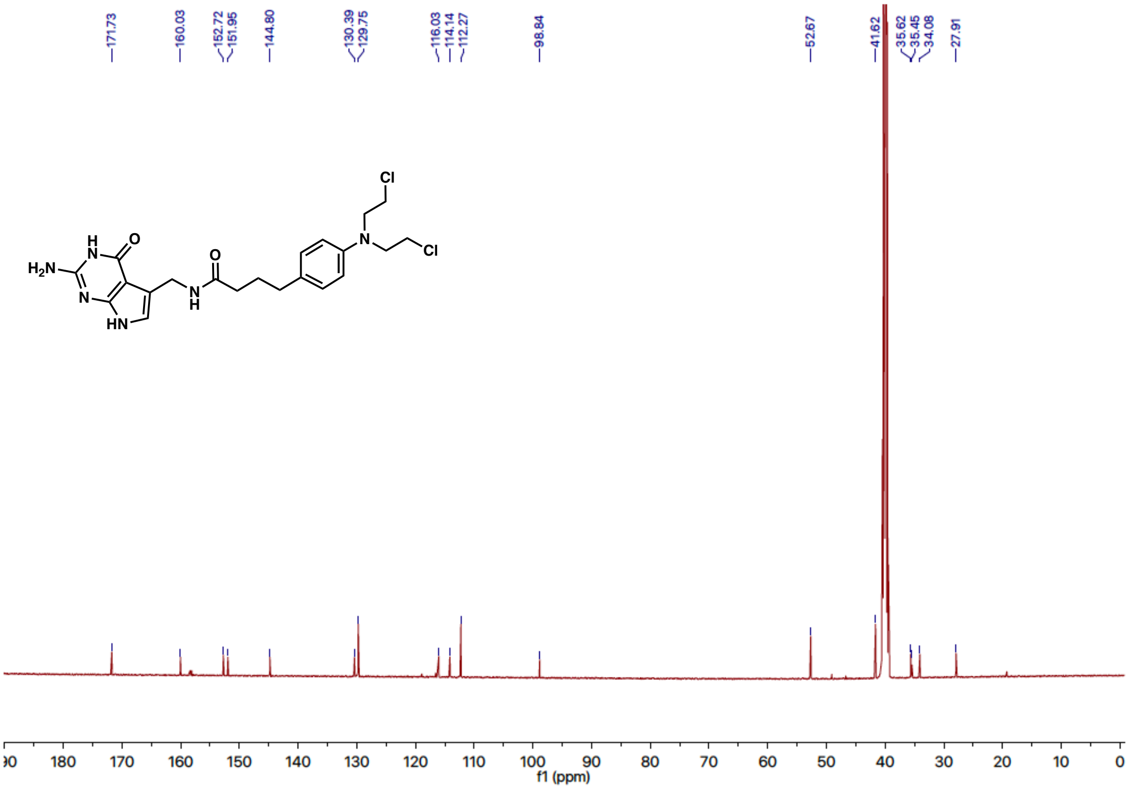

Compound 11 (2-amino-5-(((2-(3-(but-3-yn-1-yl)-3*H*-diazirin-3-yl)ethyl)amino)methyl)-3,7-dihydro-4*H*-pyrrolo[2,3-*d*]pyrimidin-4-one).

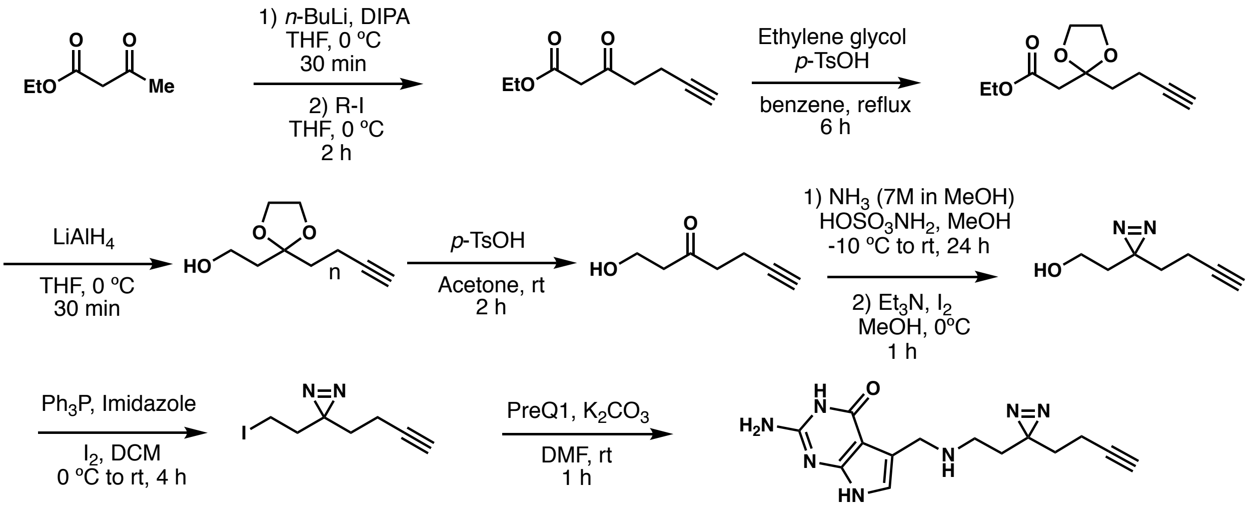

To a solution of PreQ1•2TFA (40 mg, 98 μmol, 1.0 equiv.) and K_2CO_3 (43 mg, 314 μmol, 3.2 equiv.) in DMF (1.0 mL), 3-(but-3-yn-1-yl)-3-(2-iodoethyl)-3*H*-diazirine (prepared according to the literature procedure (25 μL, 98 μmol, 1.0 equiv.) in MeCN (0.5 mL) was slowly added and stirred at room temperature for 1 h in dark. Then, the reaction mixture was concentrated and purified via column chromatography using a silica gel and eluting with gradient based MeOH:DCM to obtain the titled compound as a white solid. ^{1}H NMR (500 MHz, DMSO): δ 11.27 (s, 1H), 10.85 (brs, 1H), 8.87 (brs, 1H), 6.81 (s, 1H), 6.32 (s, 2H), 4.14 (s, 2H), 2.91-2.87 (m, 2H), 2.85 (t, *J* =2.7 Hz, 1H) , 2.00 (td, *J* = 7.3, 2.7 Hz, 2H), 1.75-171 (m, 2H), 1.63 (t, *J* = 7.3 Hz, 2H); ^{13}C{^{1}H} NMR (126 MHz, DMSO): 160.8, 153.2, 152.7, 117.9, 108.7, 98.7, 83.4, 72.4, 43.4, 41.3, 31.2, 29.8, 26.9, 13.1; HRMS m/z: calcd. for $C_{14}H_{18}N_7O$ [M+H]$^+$ 300.1567, found 300.1558.

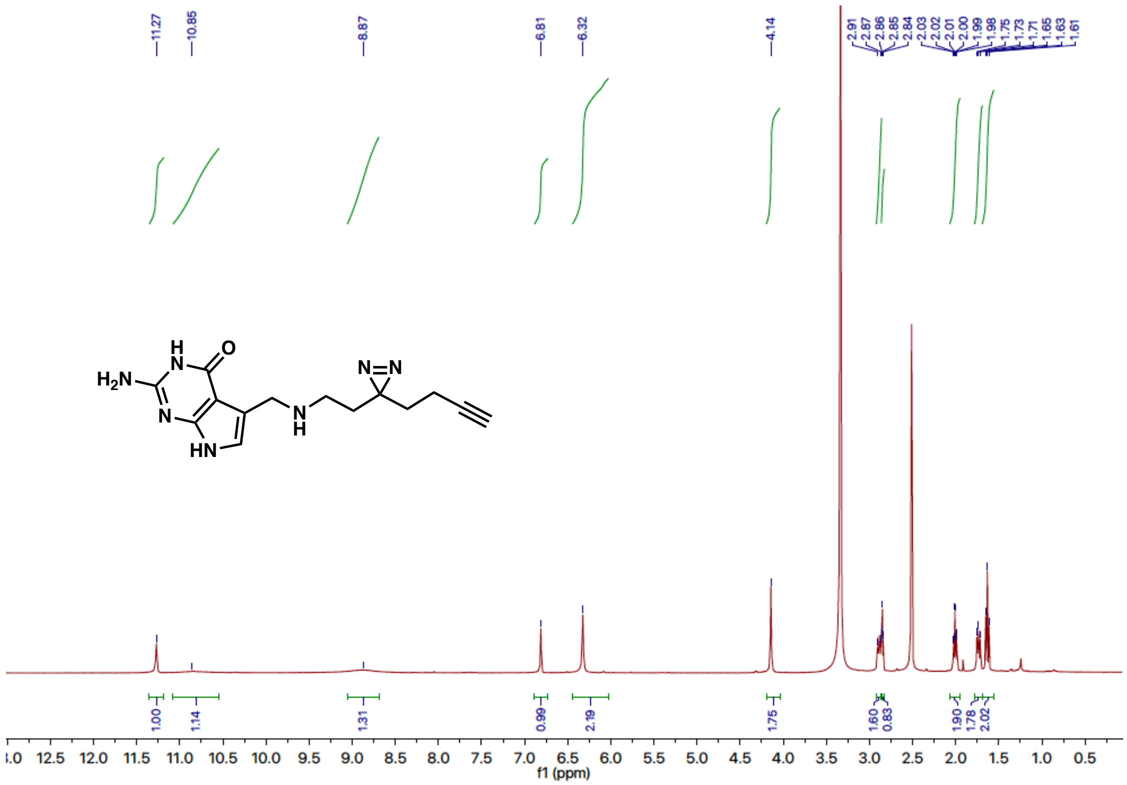
11.27
10.85
8.87
6.81
6.32
4.14
2.91
2.87
2.86
2.85
2.84
2.03
2.02
2.01
2.00
1.99
1.98
1.75
1.73
1.71
1.65
1.63
1.61
H
N
O
H2N
N
N
H
N=N
HN
1.00
1.14
1.31
0.99
2.19
1.75
1.60
0.83
1.90
1.78
2.02
f1 (ppm)

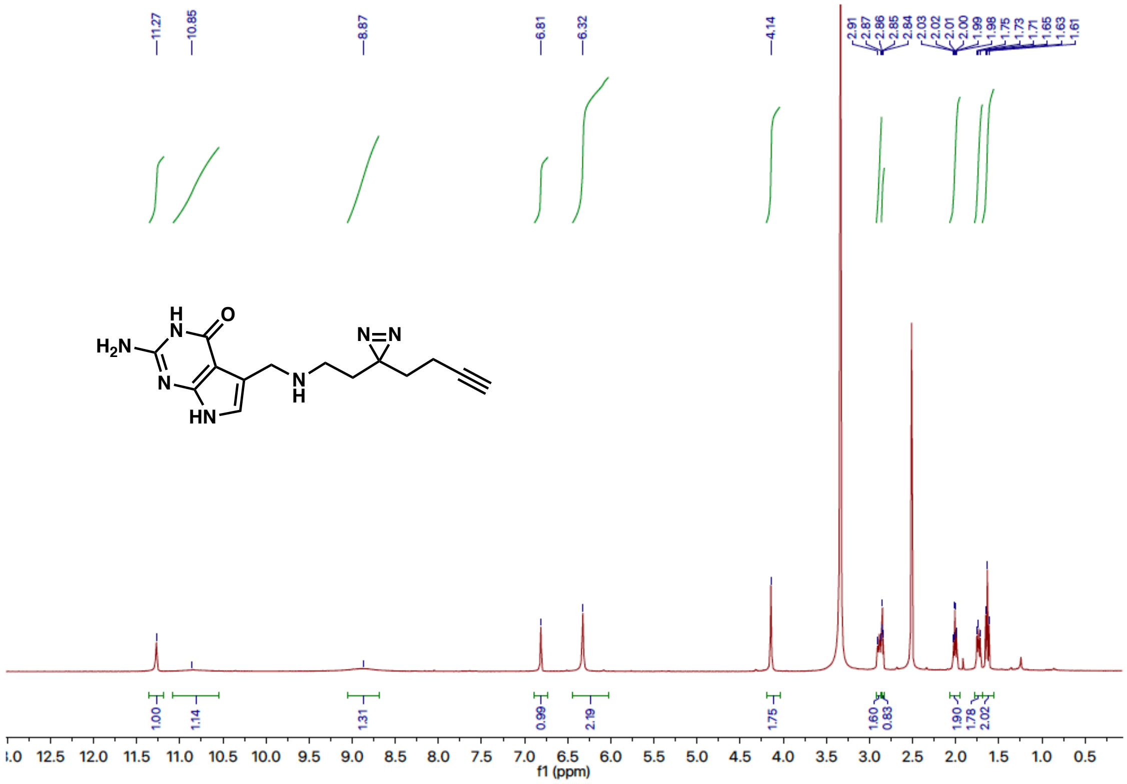
11.27
10.85
8.87
6.81
6.32
4.14
2.91
2.87
2.86
2.85
2.84
2.03
2.02
2.01
2.00
1.99
1.98
1.75
1.73
1.71
1.65
1.63
1.61
H
N
O
H2N
N
N
H
N=N
HN
1.00
1.14
1.31
0.99
2.19
1.75
1.60
0.83
1.90
1.78
2.02
f1 (ppm)

Compound 12 (*N*-((2-amino-4-oxo-4,7-dihydro-3*H*-pyrrolo[2,3-*d*]pyrimidin-5-yl)methyl)-3-(3-methyl-3*H*-diazirin-3-yl)propenamide).

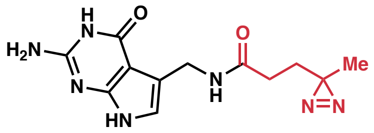

The 3-(3-methyl-3*H*-diazirin-3-yl)propanoic acid was prepared according the literature protocol. Then, compound 20 was prepared according to the general procedure **B**. White solid (12.0 mg, 85% yield). ^{1}H NMR (500 MHz, DMSO): δ 10.82, 10.32, 8.22 (t, *J* = 5.3 Hz, 1H) , 6.50 (s, 1H), 6.08 (s, 1H), 4.29 (d, *J* = 5.3 Hz, 2H) , 2.02-1.99 (m, 2H), 1.60-1.57 (m, 2H), 0.99 (s, 3H); ^{13}C{^{1}H} NMR (126 MHz, DMSO): 170.5, 160.0, 152.72, 151.91, 115.84, 114.25, 98.82, 35.58, 30.34, 30.28, 26.30, 19.78; HRMS m/z: calcd. for $C_{12}H_{16}N_7O_2$ [M+H]$^+$ 290.1360, found 290.1352.

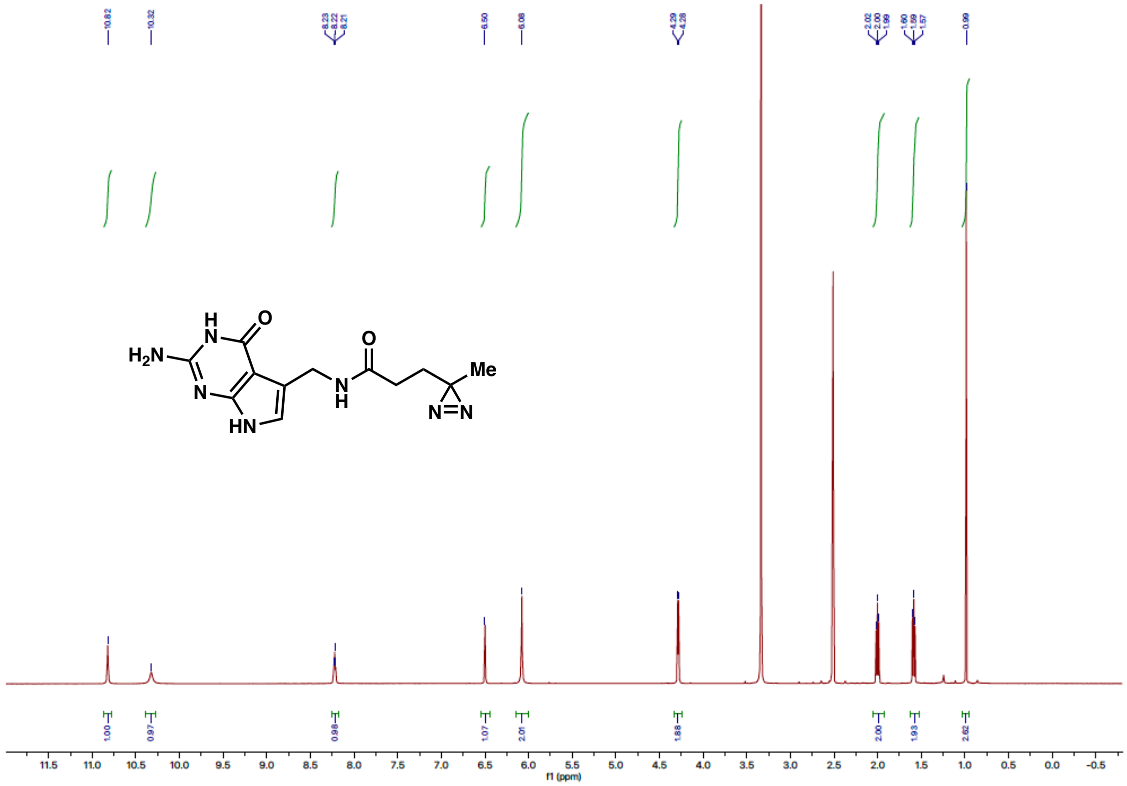

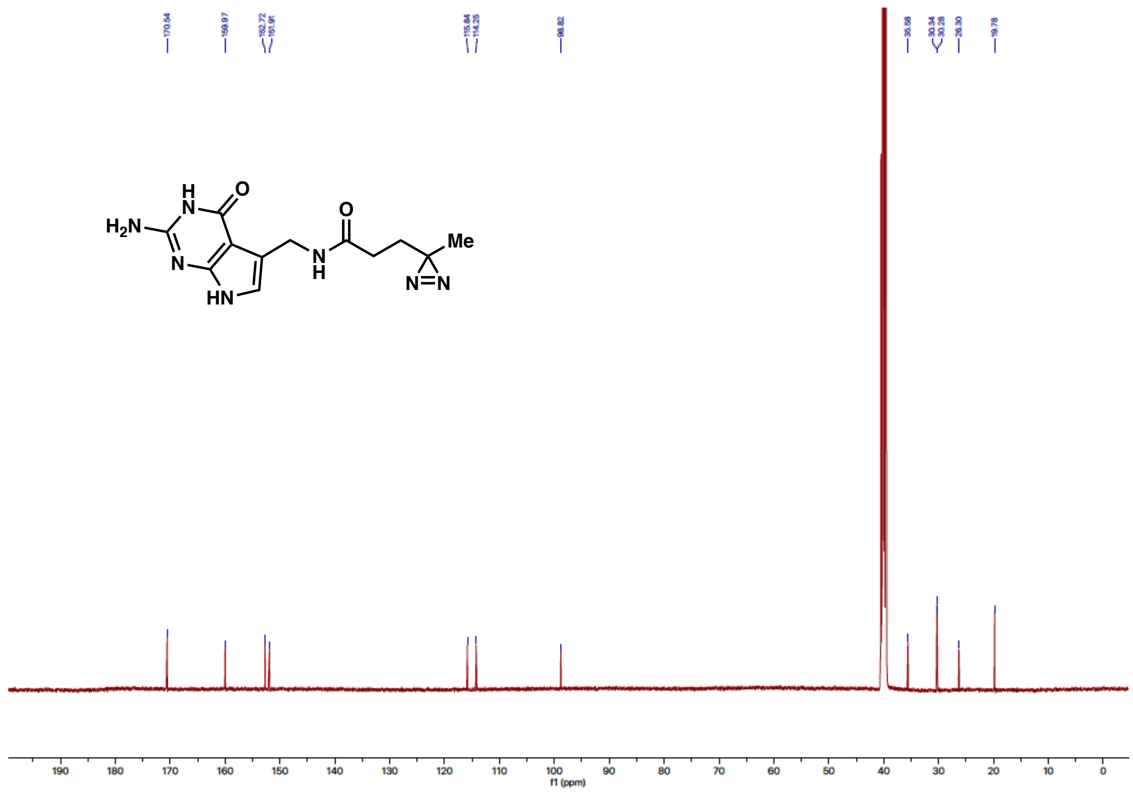

Compound 13 (*N*-((2-amino-4-oxo-4,7-dihydro-3*H*-pyrrolo[2,3-*d*]pyrimidin-5-yl)methyl)-3-(3-(but-3-yn-1-yl)-3*H*-diazirin-3-yl)propenamide).

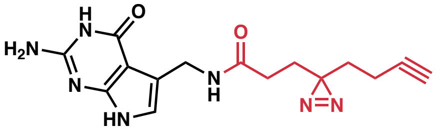

Prepared according to the general procedure **B** (Note that the reaction was performed in dark). White solid. ^{1}H NMR (500 MHz, DMSO): δ 10.83 (s, 1H), 10.34 (s, 1H), 8.21 (t, *J* = 5.4 Hz, 1H) , 6.50 (s, 1H), 6.08 (s, 2H), 4.28 (d, *J* = 5.4 Hz, 2H), 2.82 (t, *J* = 2.6 Hz, 1H), 2.00 (ddd, *J* = 7.5, 4.8, 2.6 Hz, 2H), 1.97-1.92 (m, 2H) 1.68-1.65 (m, 2H), 1.57 (t, *J* = 7.4 Hz, 2H). ^{13}C{^{1}H} NMR (126 MHz, DMSO): 170.5, 160.0, 152.7, 151.9, 115.8, 114.3, 98.8, 83.7, 72.2, 35.6, 31.9, 30.1, 28.8, 28.6, 13.1; HRMS m/z: calcd. for $C_{15}H_{18}N_7O_2$ $[M+H]^+$ 328.1516, found 328.1511.

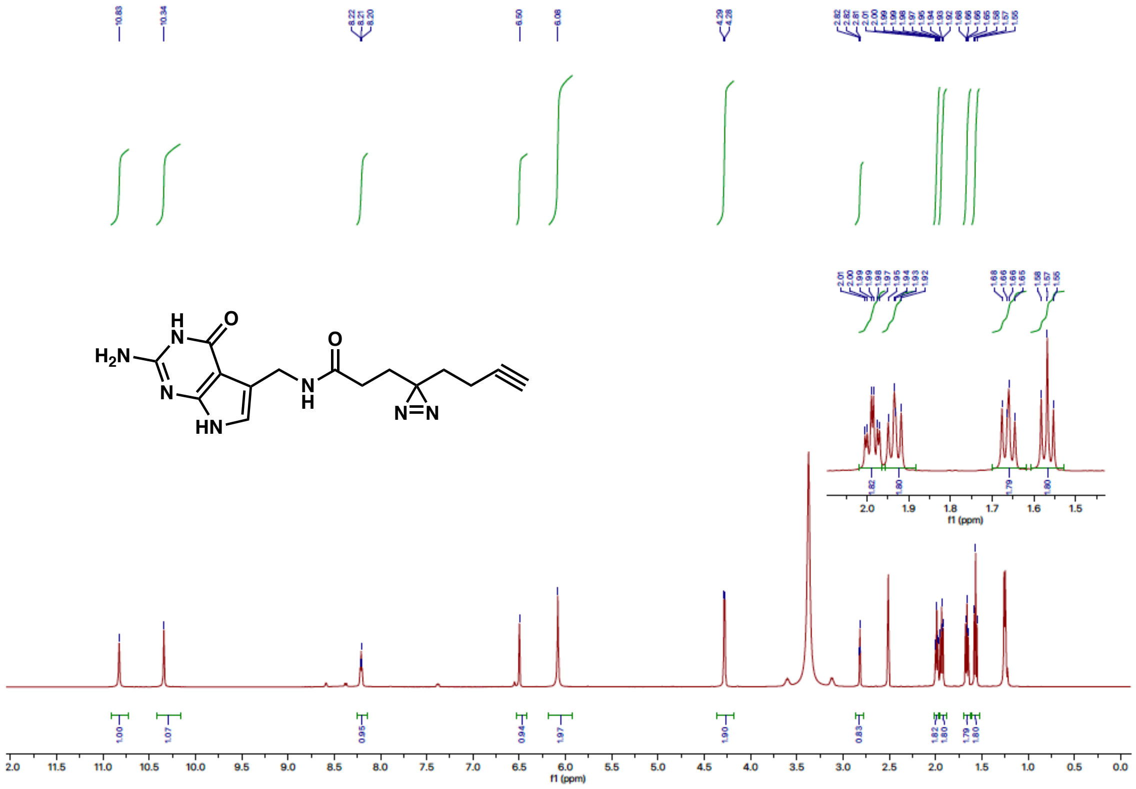

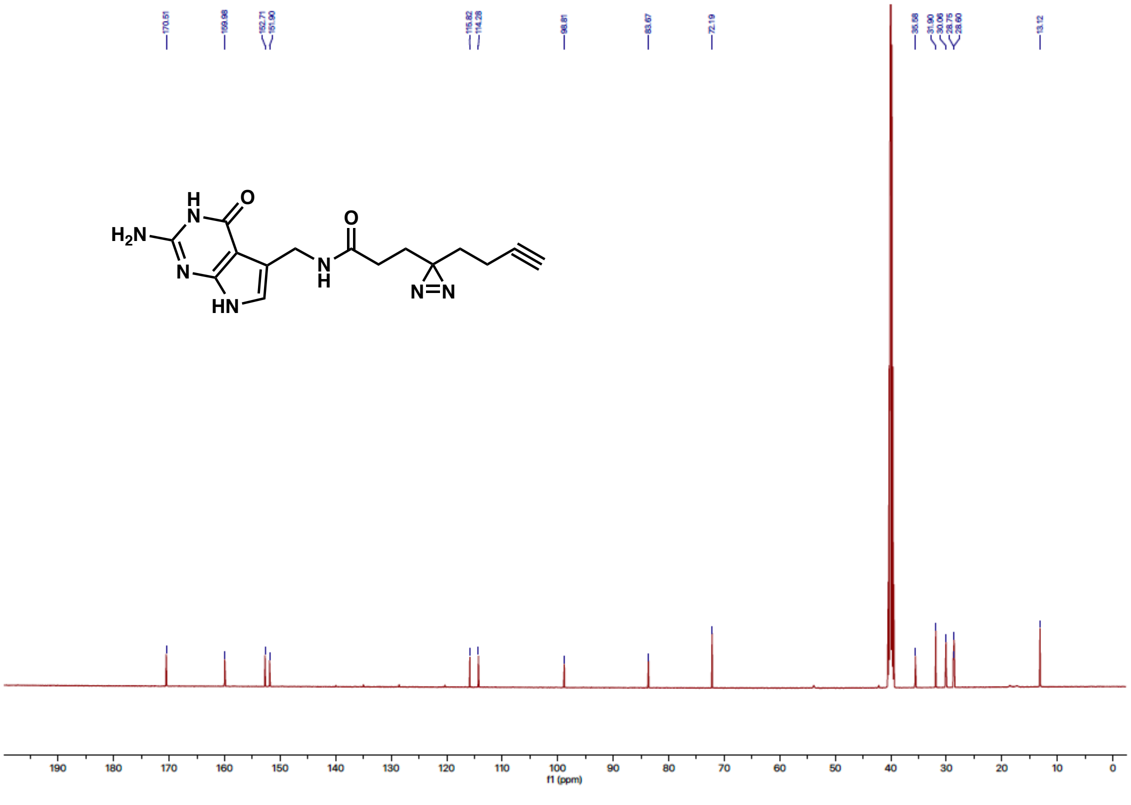

Compound 14 (2-amino-5-(((4-(3-(trifluoromethyl)-3*H*-diazirin-3-yl)benzyl)amino)methyl)-3,7-dihydro-4*H*-pyrrolo[2,3-*d*]pyrimidin-4-one).

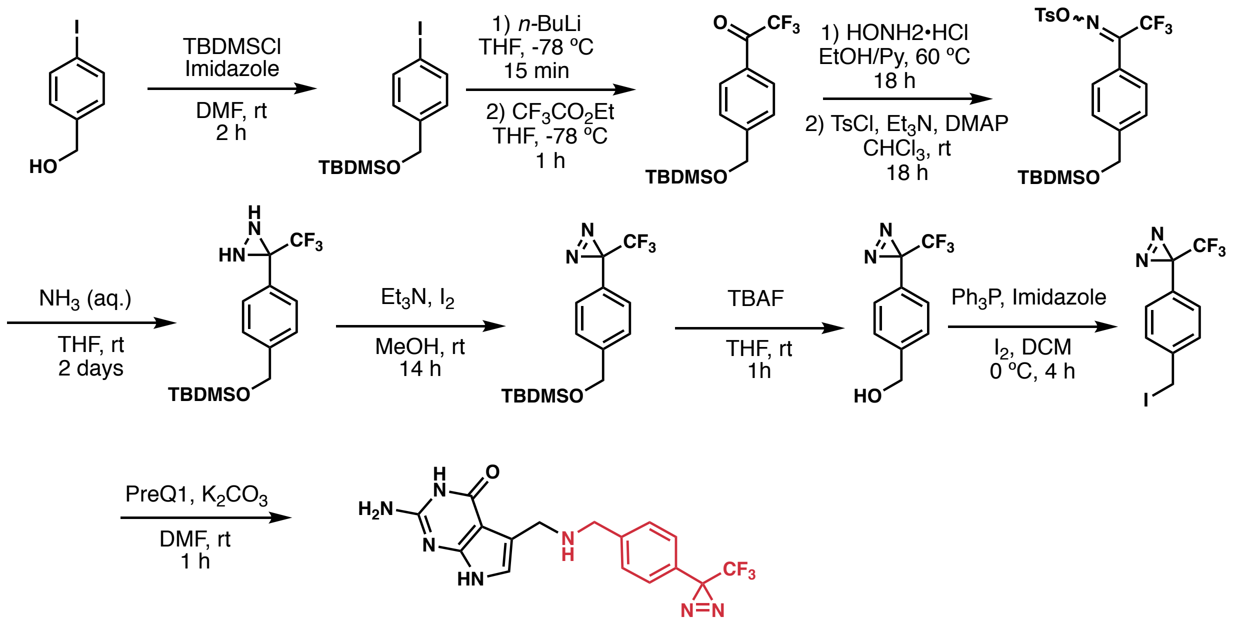

The 3-(4-(iodomethyl)phenyl)-3-(trifluoromethyl)-3*H*-diazirine was prepared according to the literature procedure. Then, compound **15** was prepared according the procedure for compound **14**. White solid. ^{1}H NMR (500 MHz, DMSO): δ 11.28 (s, 1H), 10.86 (s, 1H), 9.37 (s, 1H), 7.61 (d, J = 8.5 Hz, 2H) , 7.37 (d, J = 8.1 Hz, 2H) , 6.84 (d, J = 2.2 Hz, 1H) 6.35 (s, 2H), 4.23 (s, 2H), 4.16 (s, 2H); ^{13}C{^{1}H} NMR (126 MHz, DMSO): δ 160.9, 153.1, 152.8, 131.1, 128.4, 127.3, 123.4 (q, J = 276 Hz) , 117.7, 98.7, 49.3, 43.6, 40.9, 28.3 (q, J = 40.0 Hz); ^{19}F NMR (471 MHz, DMSO) δ -64.6; HRMS m/z: calcd. for $C_{16}H_{15}F_3N_7O$ $[M+H]^+$ 378.1285, found 378.1271.

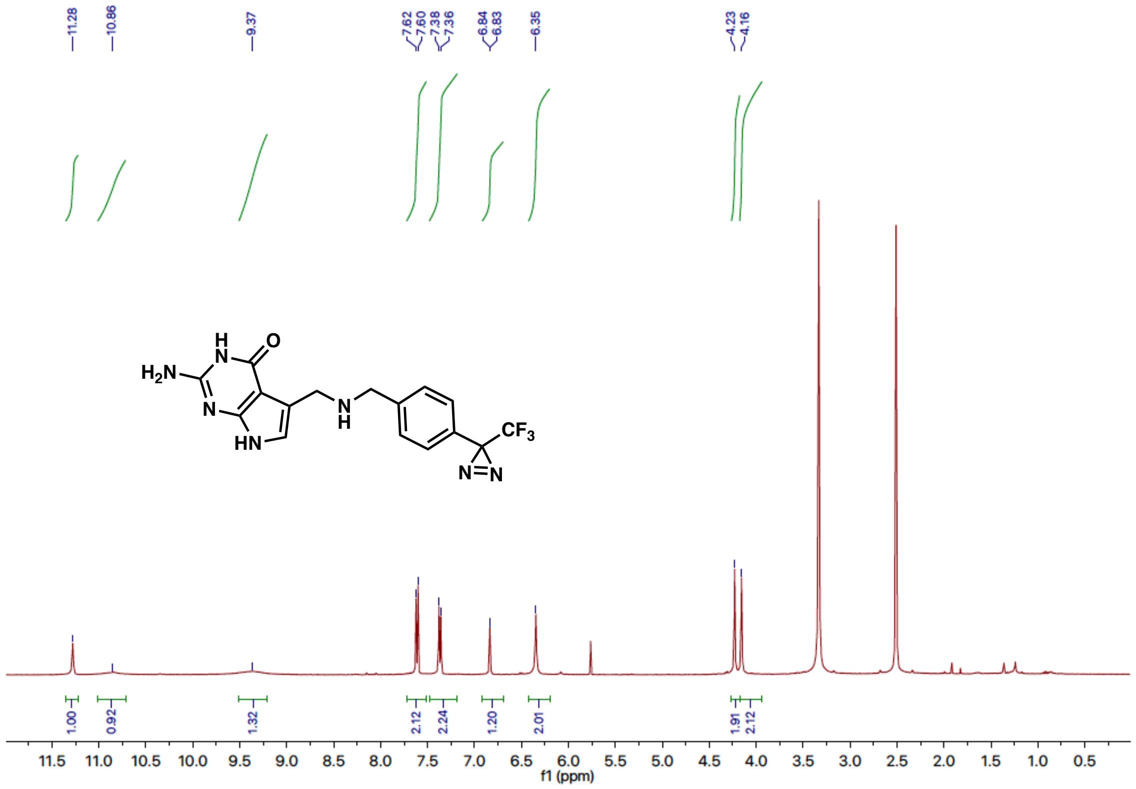
11.28
10.86
9.37
7.62
7.60
7.38
7.36
6.84
6.83
6.35
4.23
4.16
1.00
0.92
1.32
2.12
2.24
1.20
2.01
1.91
2.12
11.5 11.0 10.5 10.0 9.5 9.0 8.5 8.0 7.5 7.0 6.5 6.0 5.5 5.0 4.5 4.0 3.5 3.0 2.5 2.0 1.5 1.0 0.5
f1 (ppm)
H_2N
H
N
O
N
HN
N
H
CF_3
N=N

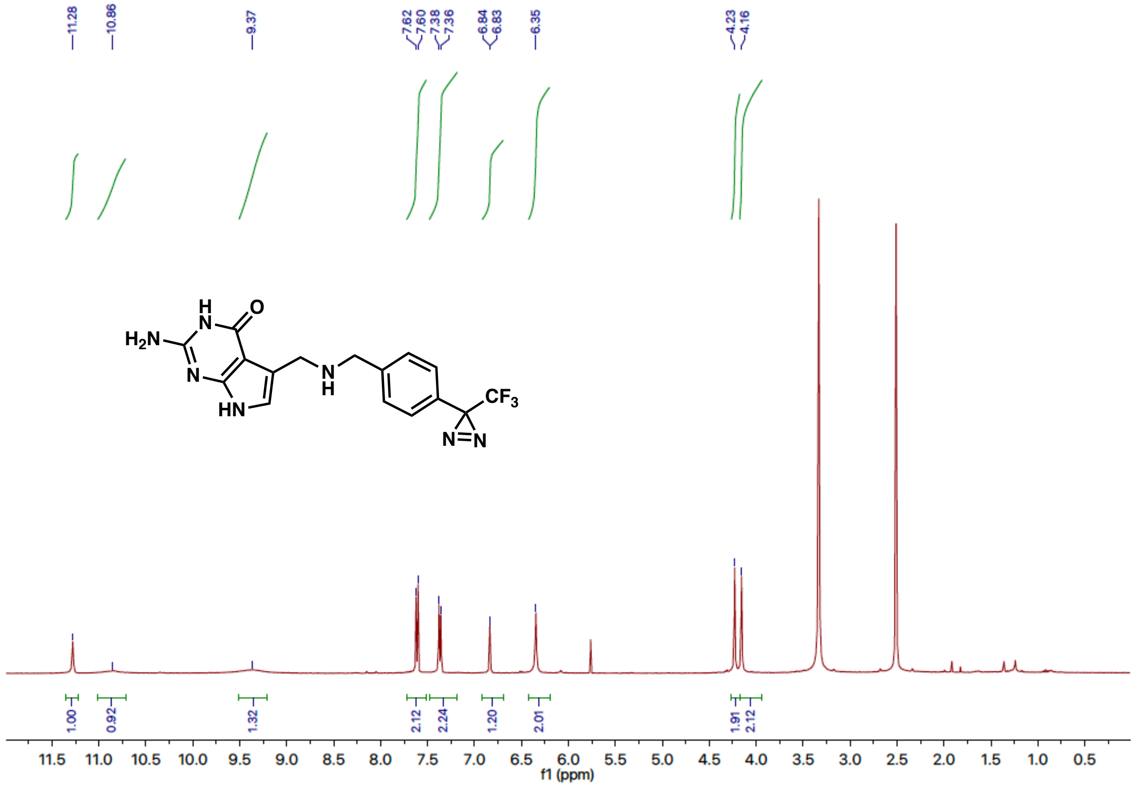
11.28
10.86
9.37
7.62
7.60
7.38
7.36
6.84
6.83
6.35
4.23
4.16
1.00
0.92
1.32
2.12
2.24
1.20
2.01
1.91
2.12
11.5 11.0 10.5 10.0 9.5 9.0 8.5 8.0 7.5 7.0 6.5 6.0 5.5 5.0 4.5 4.0 3.5 3.0 2.5 2.0 1.5 1.0 0.5
f1 (ppm)
H_2N
H
N
O
N
HN
N
H
CF_3
N=N

Compound 15 (2-amino-5-(12-(4-(3-(trifluoromethyl)-3*H*-diazirin-3-yl)phenyl)-5,8-dioxa-2,11-diazadodecyl)-3,7-dihydro-4*H*-pyrrolo[2,3-*d*]pyrimidin-4-one).

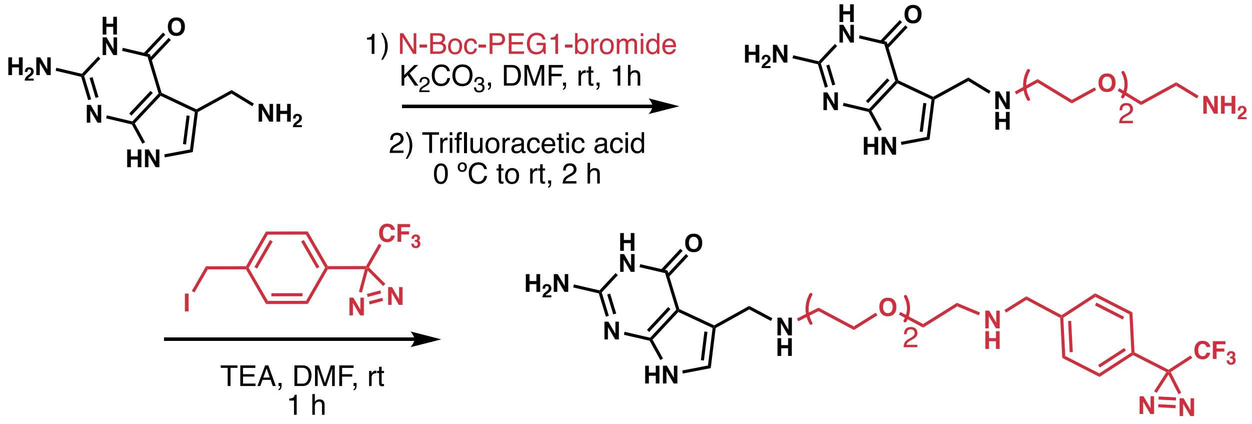

White solid. ^{1}H NMR (500 MHz, DMSO): δ 11.44 (s, 1H), 11.14 (s, 1H), 10.47 (s, 1H), 7.81 (brs, 3H), 7.64 (d, J = 8.1 Hz, 2H), 7.37 (d, J = 8.1 Hz, 2H), 6.92 (s, 1H), 6.54 (s, 2H), 4.58-4.31(m, 4H), 3.73 (m, 2H), 3.57-3.46 (m, 6H), 3.34-3.18 (m, 2H), 3.00-2.90 (m, 2H); ^{13}C{^{1}H} NMR (126 MHz, DMSO): 161.2, 158.6 (q, J = 34.6 Hz) , 153.3, 153.0, 132.9, 132.5, 129.2, 127.3, 119.4, 116.7 (q, J = 293.6 Hz), 107.3, 98.6, 69.9, 69.8, 67.1, 64.8, 56.2, 51.9, 51.1, 39.0; HRMS m/z: calcd. for $C_{22}H_{28}F_3N_8O_3$ [M+H]$^+$ 509.2231; found 509.2224.

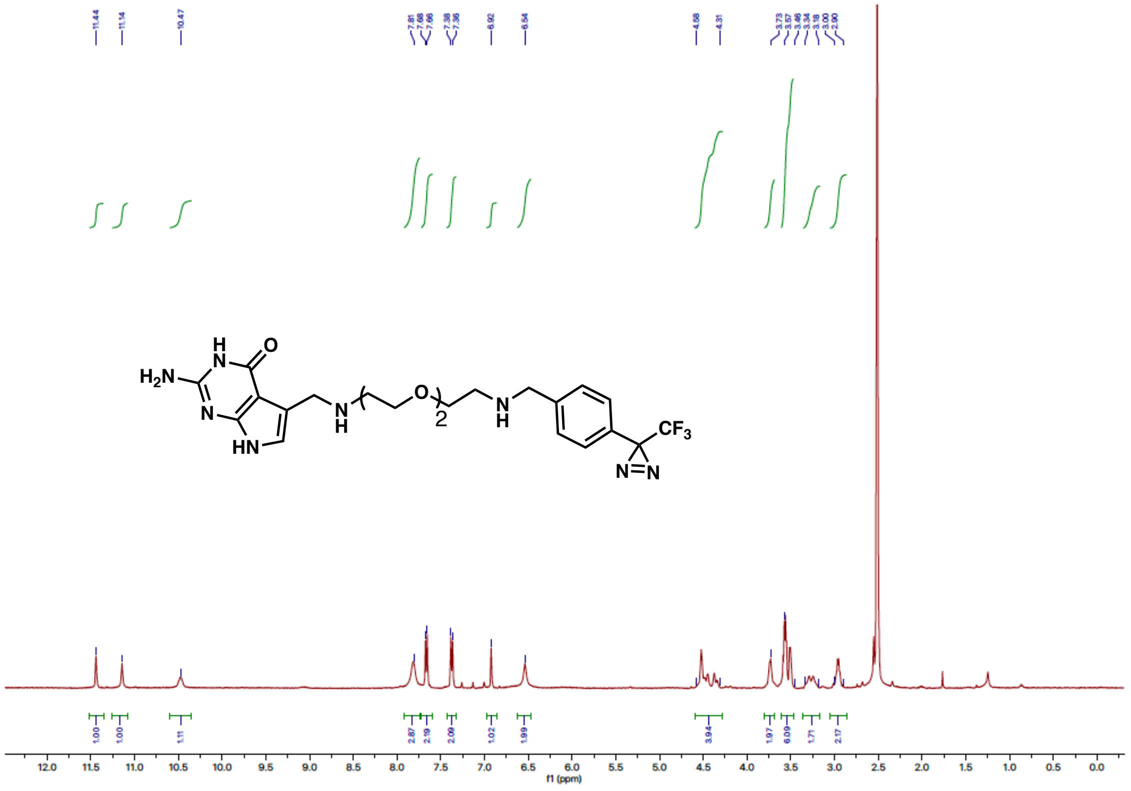

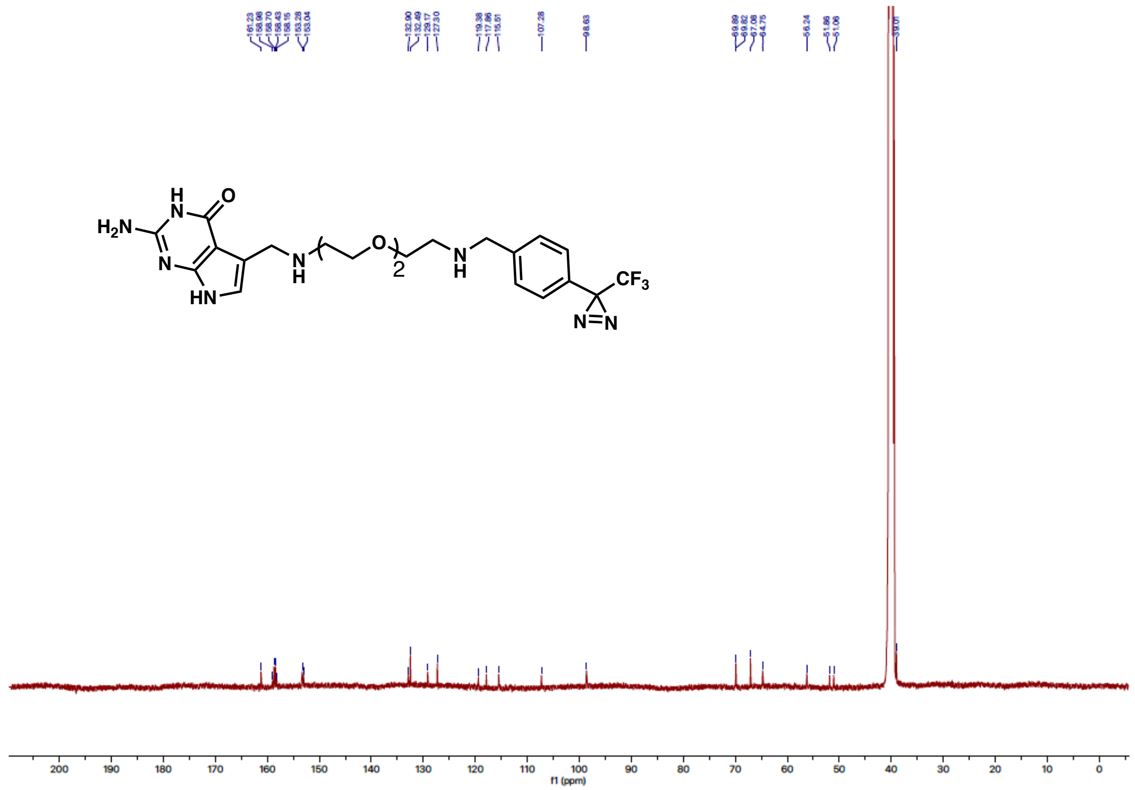

Compound 16 (2-amino-5-(15-(4-(3-(trifluoromethyl)-3*H*-diazirin-3-yl)phenyl)-5,8,11-trioxa-2,14-diazapentadecyl)-3,7-dihydro-4*H*-pyrrolo[2,3-*d*]pyrimidin-4-one).

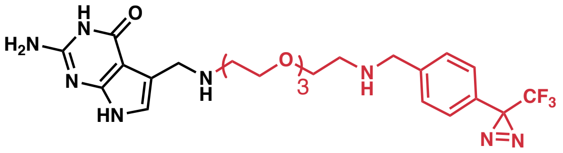

Prepared according to the procedure for compound **15**. White solid. ^{1}H NMR (500 MHz, DMSO): δ 11.43 (s, 1H), 11.11 (s, 1H), 10.48 (s, 1H) 7.78 (brs, 3H), 7.67 (d, *J* = 8.1 Hz, 2H), 7.37 (d, *J* = 8.1 Hz, 2H), 6.92 (s, 1H), 6.49 (s, 2H), 4.57-4.17 (m, 4H), 3.72 (m, 2H), 3.59-3.45 (m, 10H), 3.34-3.18 (m, 2H), 3.00-2.91 (m, 2H); ^{13}C{^{1}H} NMR (126 MHz, DMSO): 161.2, 158.6 (q, *J* = 36.0 Hz), 153.3, 153.0, 132.9, 132.5, 129.2, 127.3, 121.1, 119.4, 116.4 (q, *J* = 293.0 Hz), 107.3, 98.7, 70.0, 69.9, 67.2, 64.8, 56.3, 51.8, 51.1, 39.0; HRMS m/z: calcd. for $C_{24}H_{32}F_3N_8O_4$ $[M+H]^+$ 553.2493; found 553.2484.

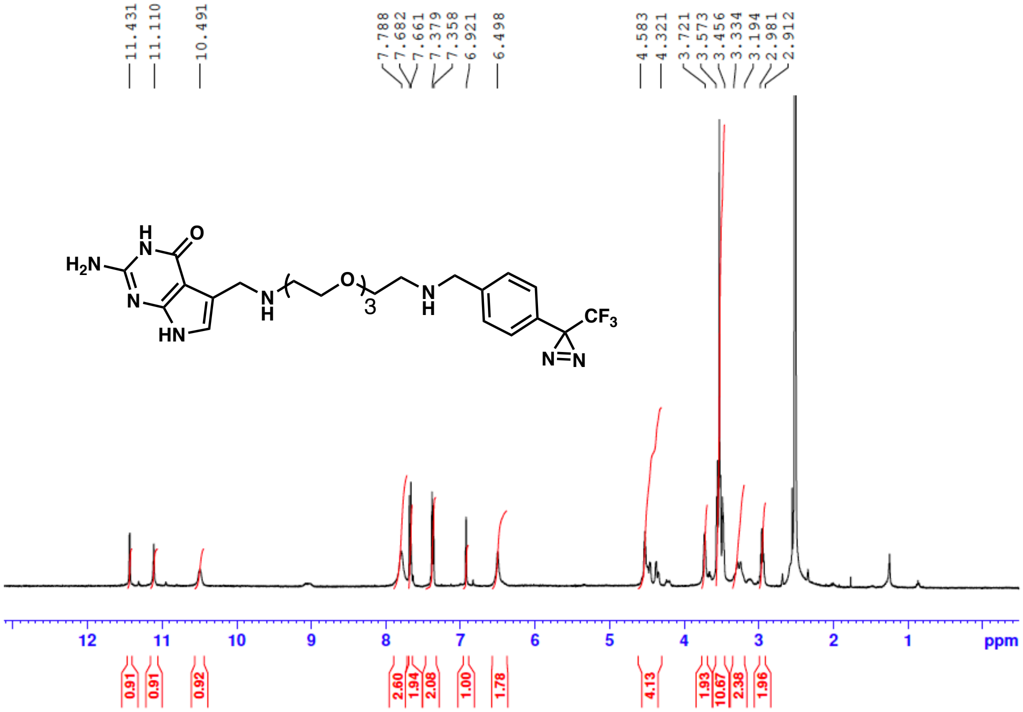

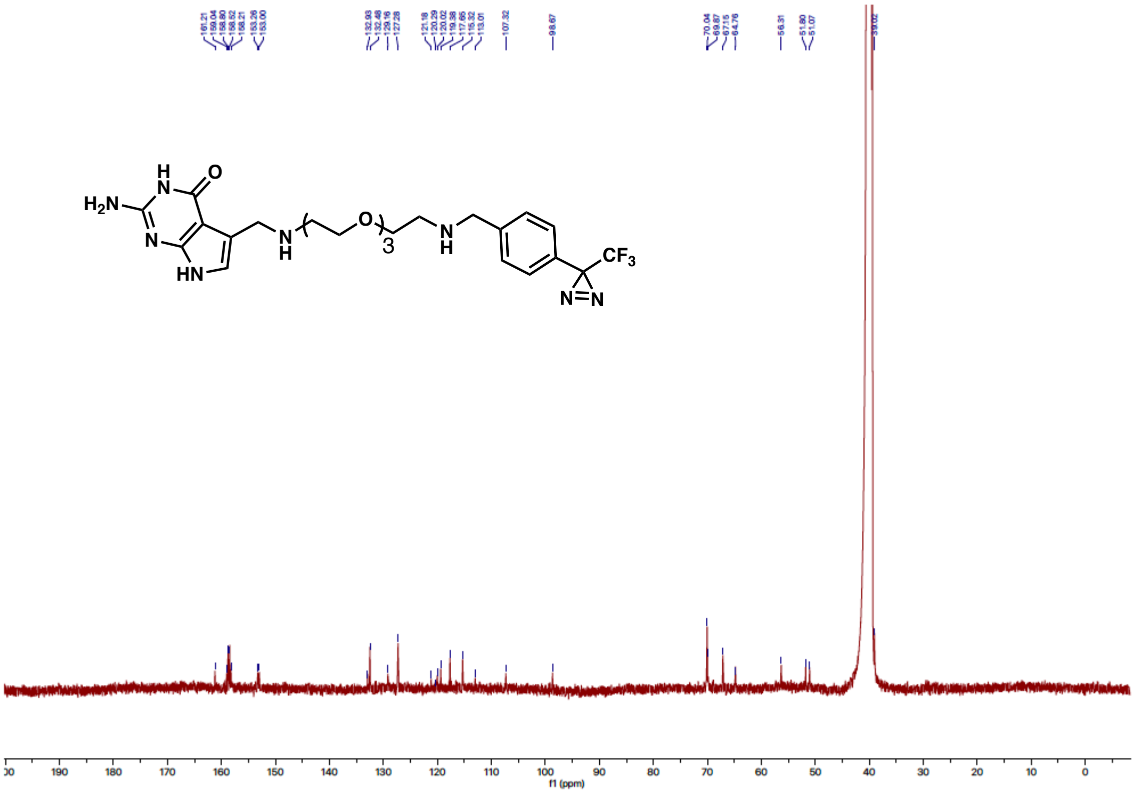

Compound 17 (*N*-((2-amino-4-oxo-4,7-dihydro-3*H*-pyrrolo[2,3-*d*]pyrimidin-5-yl)methyl)-3-(2-(2-((4-(3-(trifluoromethyl)-3*H*-diazirin-3-yl)benzyl)amino)ethoxy)ethoxy) propenamide).

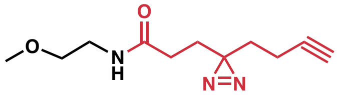

This compound was synthesized using the general procedure **B** for the coupling reaction. White solid. ^{1}H NMR (500 MHz, DMSO): δ 7.94 (s, 1H), 3.31 (d, *J* = 5.8 Hz, 2H), 3.23 (s, 3H), 3.21-3.14 (m, 2H), 2.82 (t, *J* = 2.7 Hz, 1H), 1.98 (td, *J* = 7.4, 2H), 1.94-1.86 (m, 2H), 1.62 (t, 2H), 1.56 (t, *J* = 7.4 Hz, 2H); ^{13}C{^{1}H} NMR (126 MHz, DMSO): δ 170.72, 83.18, 71.74, 70.62, 57.88, 38.36, 31.40, 29.37, 28.15, 12.65; HRMS m/z calculated for $C_{10}H_{17}N_3O$ [M+H]+ 196.2619, found 196.2599.

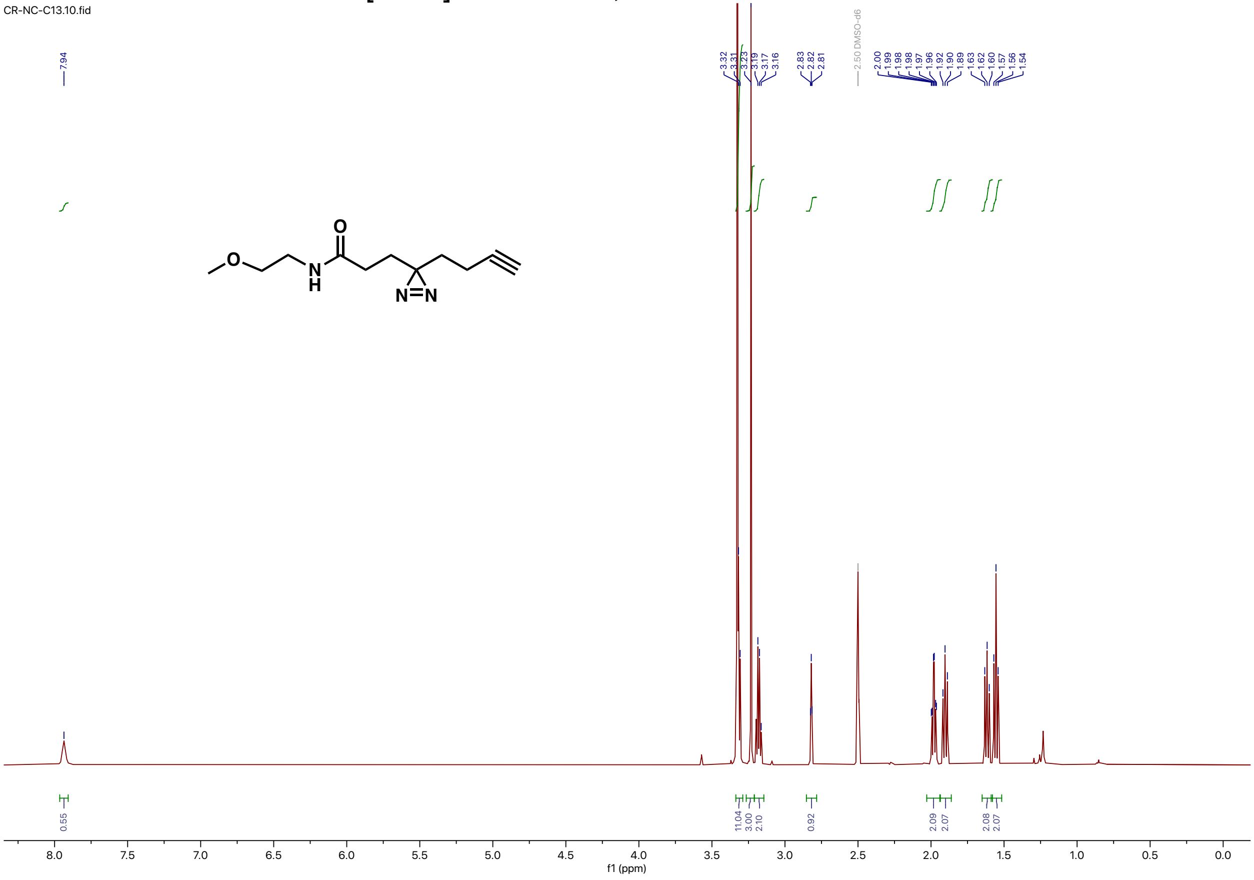

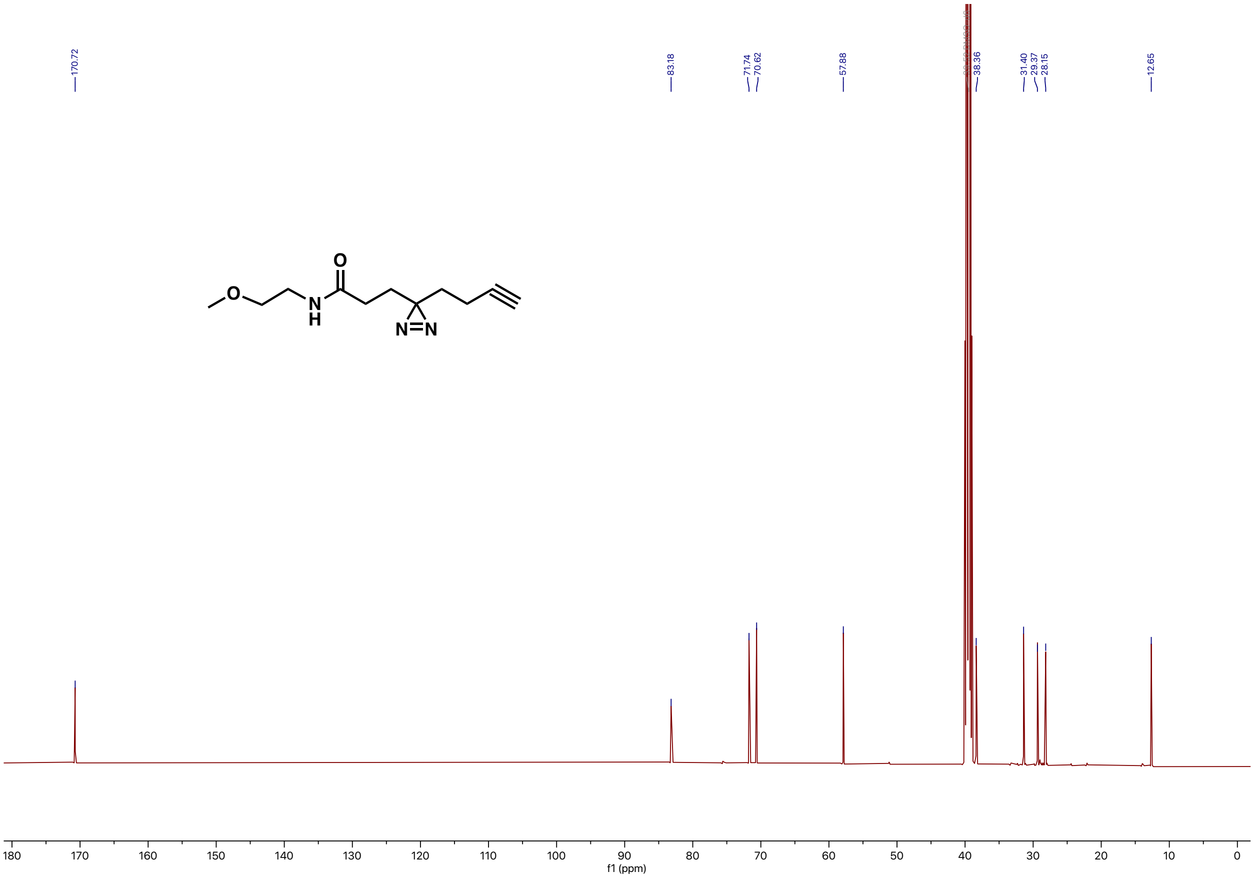

Compound Bio-11. (*N*-(2-(2-(2-(4-(2-(3-(2-(((2-amino-4-oxo-4,7-dihydro-3*H*-pyrrolo[2,3-*d*]pyrimidin-5-yl)methyl)amino)ethyl)-3*H*-diazirin-3-yl)ethyl)-1*H*-1,2,3-triazol-1-yl)ethoxy)ethoxy)ethyl)-5-((3a*R*,4*R*,6a*S*)-2-oxohexahydro-1*H*-thieno[3,4-*d*]imidazol-4-yl)pentanamide).

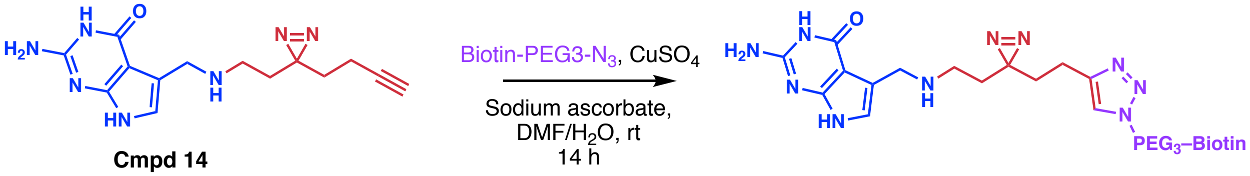

To a solution of compound **14** (30.0 mg, 100 μmol, 1.0 equiv.) and Biotin-PEG-N_3 (45 mg, 100 μmol, 1.0 equiv.) in anhydrous DMF (2.0 mL), sodium ascorbate (100 μl of 0.2 M, 20 μmol, 0.2 equiv.) and $CuSO_4$ (100 μL of 0.2M, 20 μmol, 0.2 equiv.) and the reaction mixture was stirred at room temperature for 14 hr under N_2 atmosphere. Then, the reaction mixture was concentrated under reduced pressure and purified by preparative HPLC eluting with H_2O:MeCN containing 0.1% TFA to obtain the titled compound as white solid (40.0 mg, 54% yield). ^{1}H NMR (500 MHz, DMSO): δ 11.34 (s, 1H), 11.0 (s, 1H), 9.04 (s, 2H), 7.85-7.82 (m, 2H), 6.82 (s, 1H), 6.44 (brs, 3H), 4.48 (t, J = 5.2 Hz, 2H) , 4.32 (dd, J = 7.6, 4.8 Hz, 1H), 4.18-4.12 (m, 3H), 3.79 (t, J = 5.2 Hz, 2H), 3.52-3.47 (m, 8H), 3.38 (t, J = 6.0 Hz, 2H), 3.18 (q, J = 6.0 Hz, 2H), 3.12-3.07

(m, 1H), 2.93-2.86 (m, 2H), 2.82 (dd, J = 12.4, 5.0 Hz, 1H), 2.59 (d, J = 12.4 Hz, 1H), 2.44-2.40 (m 2H), 2.06 (t, J = 7.4 Hz, 2H), 1.82-1.24 (m, 10H); $^{13}C\{^{1}H\}$ NMR (126 MHz, DMSO): 172.7, 163.3, 160.7, 153.2, 152.1, 118.0, 108.7, 98.7, 70.2, 70.1, 70.02, 70.00, 69.6, 69.2, 61.5, 59.7, 55.9, 49.8, 43.3, 41.3, 38.9, 35.6, 31.6, 29.9, 28.6, 28.5, 27.1, 25.7, 20.0; HRMS m/z: calcd. for $C_{32}H_{50}N_{13}O_6S$ $[M+H]^+$ 744.3722, found 744.3716.

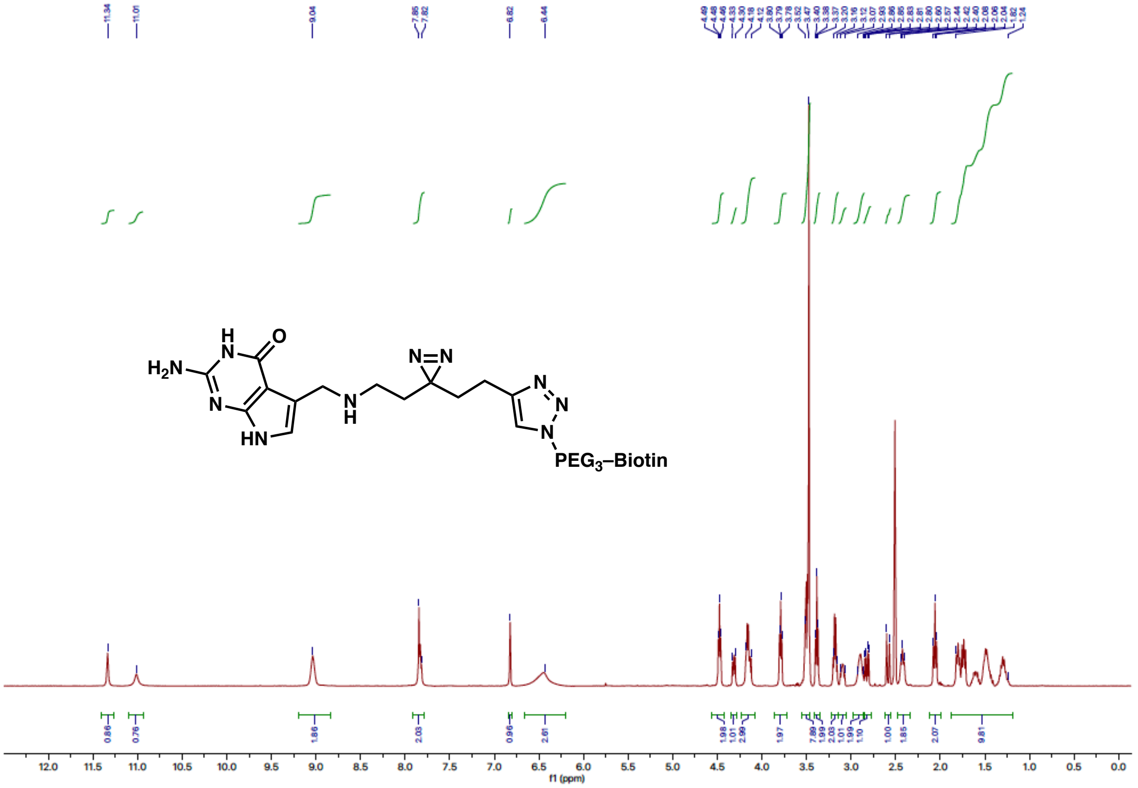

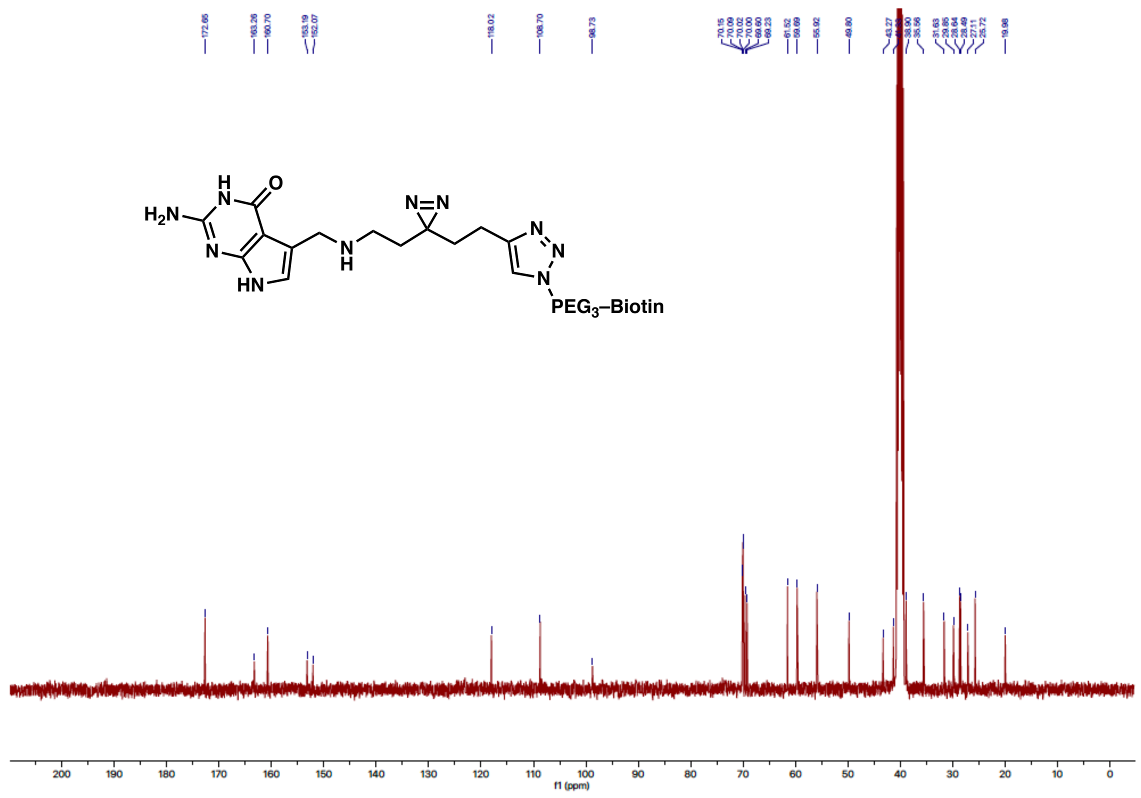

References:

1. Weiss, M. S. Global indicators of X-ray data quality. *J Appl Crystallogr* **34**, 130-135 (2001).

2. Karplus, P. A. & Diederichs, K. Linking crystallographic model and data quality. *Science* **336**, 1030-1033 (2012).
