# Supplementary material for: A chemical probe based on the PreQ1 metabolite enables transcriptome-wide mapping of binding sites
Source: Nat Commun. 2021 Oct 6;12:5856. doi: 10.1038/s41467-021-25973-x (PMC8494917; doi:10.1038/s41467-021-25973-x)
Supplement: Supplementary file 2 — Reporting Summary [file 41467_2021_25973_MOESM2_ESM.pdf]

nature portfolio

Corresponding author(s): John S. Schneekloth Jr.

Last updated by author(s): Aug 13, 2021

Reporting Summary

Nature Portfolio wishes to improve the reproducibility of the work that we publish. This form provides structure for consistency and transparency in reporting. For further information on Nature Portfolio policies, see our Editorial Policies and the Editorial Policy Checklist.

Statistics

For all statistical analyses, confirm that the following items are present in the figure legend, table legend, main text, or Methods section.

n/a	Confirmed	
☐	☒	The exact sample size (*n*) for each experimental group/condition, given as a discrete number and unit of measurement
☐	☒	A statement on whether measurements were taken from distinct samples or whether the same sample was measured repeatedly
☐	☒	The statistical test(s) used AND whether they are one- or two-sided<br>*Only common tests should be described solely by name; describe more complex techniques in the Methods section.*
☒	☐	A description of all covariates tested
☒	☐	A description of any assumptions or corrections, such as tests of normality and adjustment for multiple comparisons
☐	☒	A full description of the statistical parameters including central tendency (e.g. means) or other basic estimates (e.g. regression coefficient) AND variation (e.g. standard deviation) or associated estimates of uncertainty (e.g. confidence intervals)
☒	☐	For null hypothesis testing, the test statistic (e.g. *F*, *t*, *r*) with confidence intervals, effect sizes, degrees of freedom and *P* value noted<br>*Give P values as exact values whenever suitable.*
☒	☐	For Bayesian analysis, information on the choice of priors and Markov chain Monte Carlo settings
☒	☐	For hierarchical and complex designs, identification of the appropriate level for tests and full reporting of outcomes
☐	☒	Estimates of effect sizes (e.g. Cohen's *d*, Pearson's *r*), indicating how they were calculated

Our web collection on statistics for biologists contains articles on many of the points above.

Software and code

Policy information about availability of computer code

Data collection	Information about the specific commercial software/s used to collect data is included in the manuscript (methods section). ZOO
Data analysis	Information about the specific commercial software/s used to analyze data is included in the manuscript (methods section). ImageJ, Prism8, DeSeq2, Xtract, KAMO, XDS, PHASER, PHENIX, COOT, ImageQuant, MestReNova

For manuscripts utilizing custom algorithms or software that are central to the research but not yet described in published literature, software must be made available to editors and reviewers. We strongly encourage code deposition in a community repository (e.g. GitHub). See the Nature Portfolio guidelines for submitting code & software for further information.

Data

Policy information about availability of data

All manuscripts must include a data availability statement. This statement should provide the following information, where applicable:

- Accession codes, unique identifiers, or web links for publicly available datasets
- A description of any restrictions on data availability
- For clinical datasets or third party data, please ensure that the statement adheres to our policy

For RNA sequencing data, raw FASTQ files along with processed alignment and count files have been deposited on the NCBI's gene expression omnibus (GEO) database under the accession number GSE168353. Atomic coordinates for the X-ray structures obtained here have been uploaded to the protein data base (PDB) under the codes 7E9E and 7E9I. Previously published crystals (PDB IDs: 3Q50, 3K1V and 6E1W) were also used for reference in this project.

Field-specific reporting

Please select the one below that is the best fit for your research. If you are not sure, read the appropriate sections before making your selection.

☒ Life sciences ☐ Behavioural & social sciences ☐ Ecological, evolutionary & environmental sciences

For a reference copy of the document with all sections, see nature.com/documents/nr-reporting-summary-flat.pdf

Life sciences study design

All studies must disclose on these points even when the disclosure is negative.

Sample size	Data are reported as the average of at least 3 independent replicates. For the RNA sequencing four replicates were used to increase the statistical power of the analysis. No calculations were performed to pre-determine sample size however, statistical analysis between replicates showed reproducibly significant differences between treatment groups that suggest that sample size was sufficient (t-test etc.).
Data exclusions	No data were excluded.
Replication	Experiments were repeated successfully at least 3 times. All attempts to replicate were successful
Randomization	Samples were randomly allocated into treatment or control groups.
Blinding	Blinding was not relevant to this study because none of the experiments conducted produce information that may influence the researchers conducting the experiments and it was necessary for the researchers to know the previous conclusions to continue on the project.

Reporting for specific materials, systems and methods

We require information from authors about some types of materials, experimental systems and methods used in many studies. Here, indicate whether each material, system or method listed is relevant to your study. If you are not sure if a list item applies to your research, read the appropriate section before selecting a response.

Materials & experimental systems

n/a	Involved in the study	
☒	☐	Antibodies
☐	☒	Eukaryotic cell lines
☒	☐	Palaeontology and archaeology
☒	☐	Animals and other organisms
☒	☐	Human research participants
☒	☐	Clinical data
☒	☐	Dual use research of concern

Methods

n/a	Involved in the study	
☒	☐	ChIP-seq
☒	☐	Flow cytometry
☒	☐	MRI-based neuroimaging

Eukaryotic cell lines

Policy information about cell lines

Cell line source(s)	MCF-7 Cells were acquired from ATCC.
Authentication	Authentication data were provided by ATCC at the time of purchase. However, no additional authentication was performed prior to use in this study.
Mycoplasma contamination	These cells tested negative for mycoplasma.
Commonly misidentified lines (See ICLAC register)	No commonly misidentified cell lines were used in this study.

March 2021
